# Supplementary material for: Sugar-based bactericides targeting phosphatidylethanolamine-enriched membranes
Source: Nat Commun. 2018 Nov 19;9:4857. doi: 10.1038/s41467-018-06488-4 (PMC6242839; doi:10.1038/s41467-018-06488-4)
Supplement: Supplementary file 1 — Supplementary Information [file 41467_2018_6488_MOESM1_ESM.pdf]

Supplementary Information

# Sugar-Based Bactericides Targeting Phosphatidylethanolamine-Enriched Membranes

Catarina Dias, João P. Pais, Rafael Nunes, Maria-Teresa Blázquez-Sánchez, Joaquim T. Marquês, Andreia F. Almeida, Patrícia Serra, Nuno M. Xavier, Diogo Vila-Viçosa, Miguel Machuqueiro, Ana S. Viana, Alice Martins, Maria S. Santos, Ana Pelerito, Ricardo Dias, Rogério Tenreiro, Maria C. Oliveira, Marialessandra Contino, Nicola A. Colabufo, Rodrigo F. M. de Almeida, Amélia P. Rauter*

*Correspondence to: aprauter@fc.ul.pt

This file includes:

| | Page |
|---|---|
| **1. Supplementary Figures** | 3 |
| **2. Supplementary Tables** | 42 |
| **3. Supplementary Methods** | 51 |
| 3.1 Synthesis | 51 |
| 3.2 Biological Studies | 71 |
| 3.3 Computational Studies | 74 |
| **4. Supplementary Discussion** | 77 |
| 4.1 Conformational analysis of *threo*-pentopyranosides | 77 |
| **5. Supplementary References** | 78 |

# 1 | Supplementary Figures

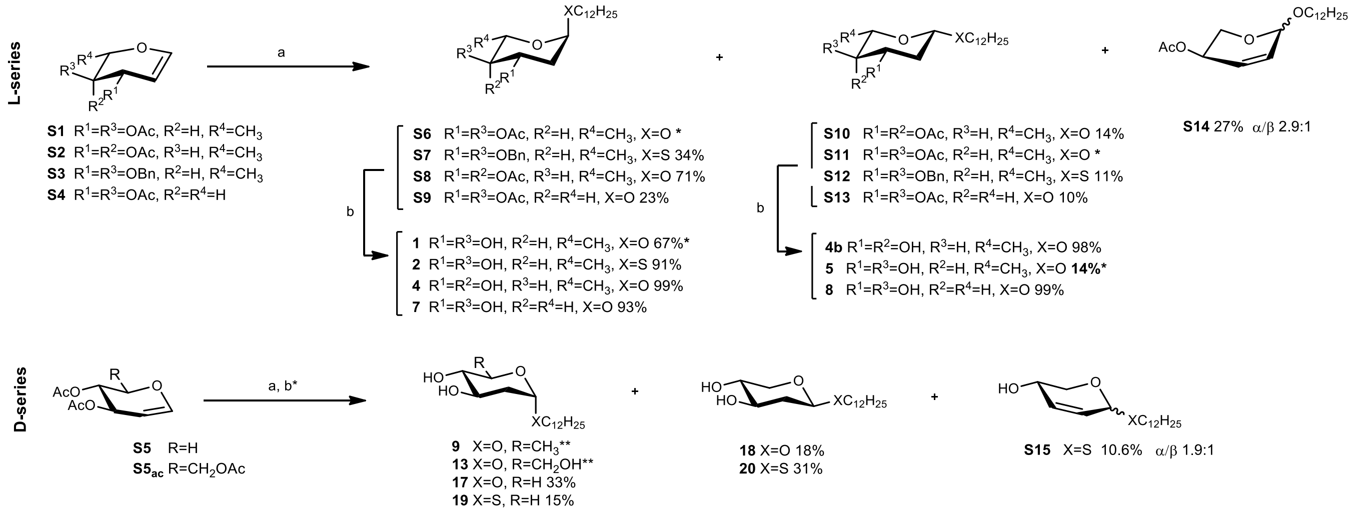


**Supplementary Fig. 1. Synthesis of dodecyl 2-deoxy glycosides.** Reagents and conditions: a) $C_{12}H_{25}XH$, TPHB, DCM; b) NaOMe/MeOH (for compounds **1**,**5**, **4**, **4b**, **7**, **8**, **17-20**) or $H_2$, Pd/C, MeOH (for compounds **S7**, **S12**); *one-pot procedure; **synthesized starting from **S5**[1].

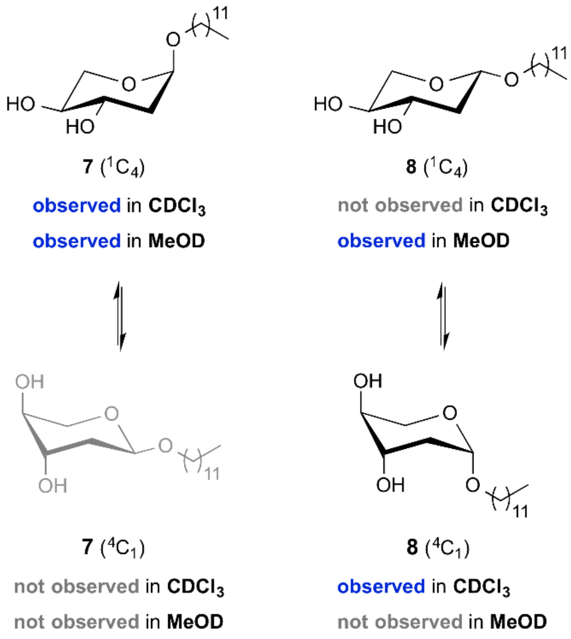


**Supplementary Fig. 2. Conformational preferences of dodecyl 2-deoxy-*threo*-pentopyranosides.** The conformers detected by NMR in chloroform-*d* and methanol-$d_4$ for dodecyl 2-deoxy-α-L-*threo*-pentopyranoside **7** (left) and dodecyl 2-deoxy-β-L-*threo*-pentopyranoside **8** (right).

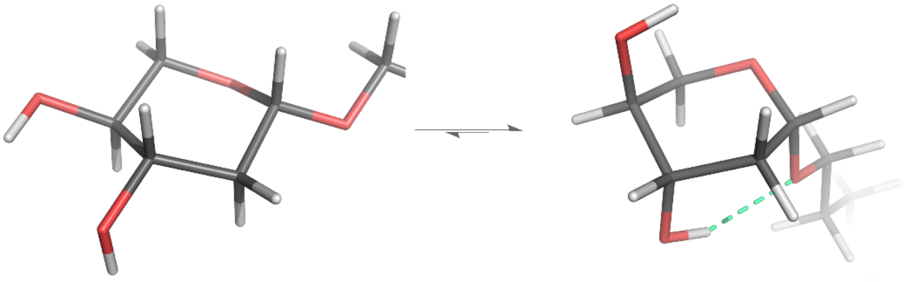

$^{4}C_{1}$–**8** *(0.0 kcal mol$^{-1}$)* $^{1}C_{4}$–**8** *(–1.0 kcal mol$^{-1}$)*

**Supplementary Fig. 3. DFT-optimized geometry for the two chair conformers of dodecyl 2-deoxy-β-L-*threo*-pentopyranoside 8 in chloroform.** The intramolecular hydrogen bond observed in the lower energy conformation is highlighted as light green dashes.

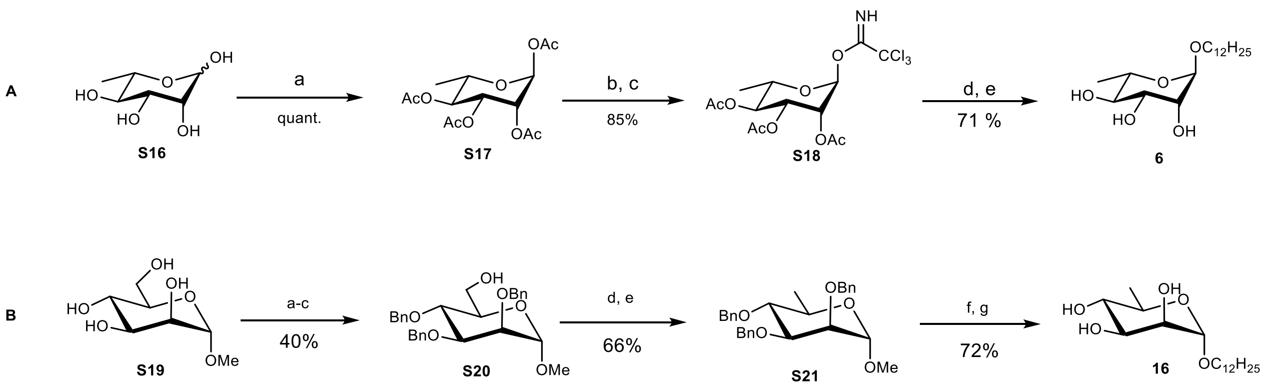


**Supplementary Fig. 4. A) Synthesis of dodecyl 6-deoxy-α-L-mannopyranoside**. Reagents and conditions: a) $Ac_2O$, py., DMAP; b) $NH_2NH_2$.AcOH, DMF; c) $Cl_3CCN$, DBU, DCM; d) $C_{12}H_{25}OH$, TMSOTf, DCM, 4Å MS, 0 ºC; e) NaOMe, MeOH. **B) Synthesis of dodecyl 6-deoxy-α-D-mannopyranoside**. Reagents and conditions: a) TrCl, DMAP, py; b) BnBr, NaH, DMF (71% over two steps); c) $I_2$, MeOH (57%); d) $I_2/Ph_3P$/imidazole, toluene; e) $LiAlH_4$, THF (66% over two steps); f) $C_{12}H_{25}OH$, A-15, reflux, 10 h; g) $H_2$, Pd/C.

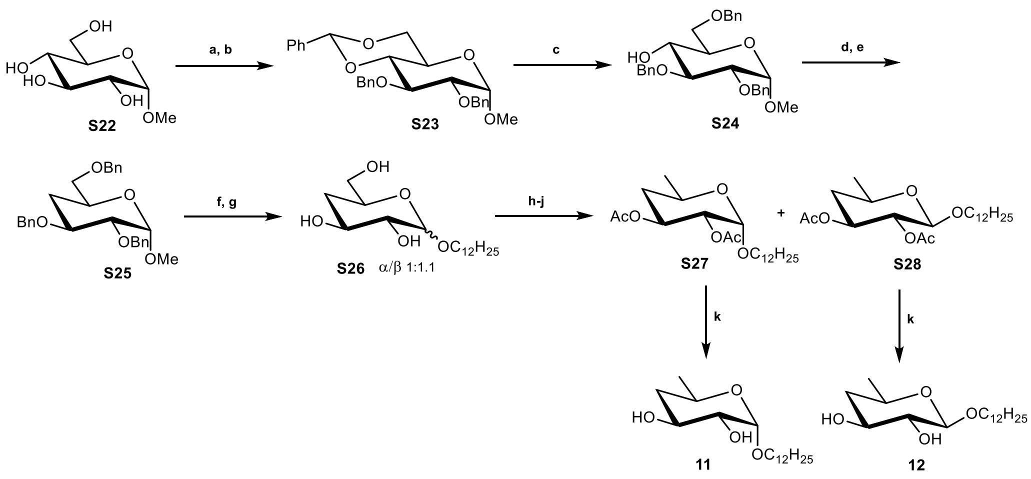


**Supplementary Fig. 5. Synthesis of dodecyl 4-deoxy- and 4,6-dideoxy-D-*xylo*-hexopyranosides.** Reagents and conditions: a) $PhCH(OMe)_2$, *p*-TsOH, DMF, 60 ºC, 240 mbar, 94%; b) BnBr, NaH, DMF, 82%; c) $NaBH_3CN$, $I_2$, MeCN, 77%; d) $Tf_2O$, py., DCM, -10 ºC; e) *n*-$Bu_4NBH_4$, THF, 83% (over two steps); f) $C_{12}H_{25}OH$, A-15; g) $Et_3SiH$, 10% Pd/C, EtOAc, 80% (over two-steps); h) $Tf_2O$, py., DCM, -10 ºC; i) $LiAlH_4$, THF; j) $Ac_2O$, py., DMAP; (41% over 3 steps); k) NaOMe, MeOH (94-96%).

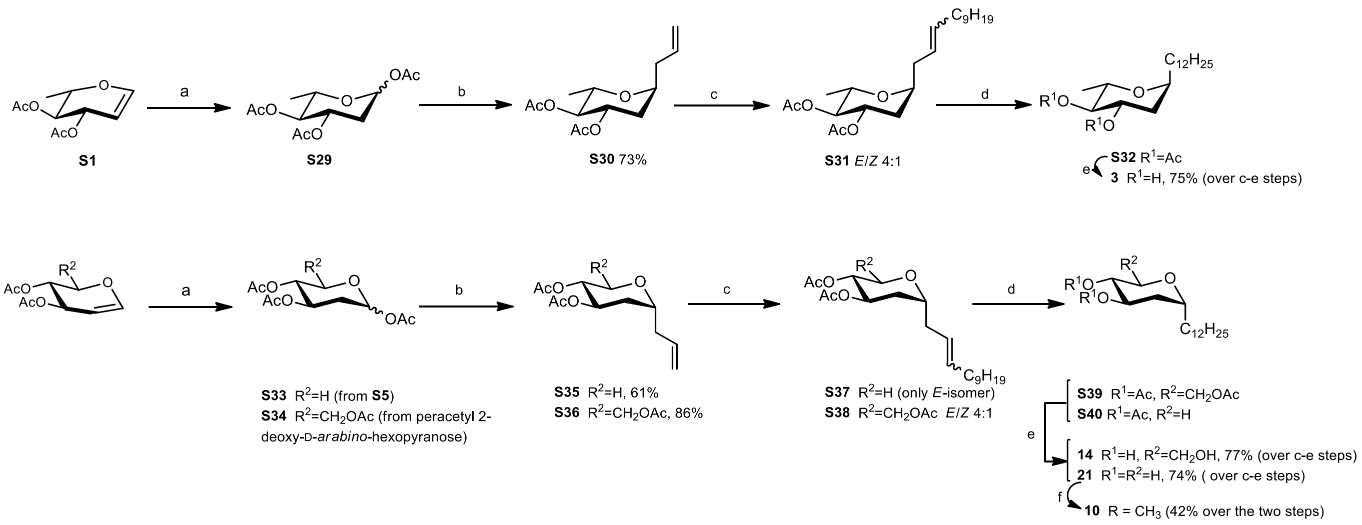


**Supplementary Fig. 6. Synthetic pathway for *C*-glycosides.** Reagents and conditions: a) $PPh_3$•HBr, AcOH, DCM; b) $H_2C{=}CHCH_2Si(CH_3)_3$, $BF_3$•$Et_2O$, ACN, 0 ºC; c) undec-1-ene, 2$^{nd}$ generation Grubbs-Hoveyda catalyst (5%), d) $H_2$, Pd/C, EtOAc; e) NaOMe, MeOH; f) i. TsCl, Py.; ii: $LiAlH_4$, THF.

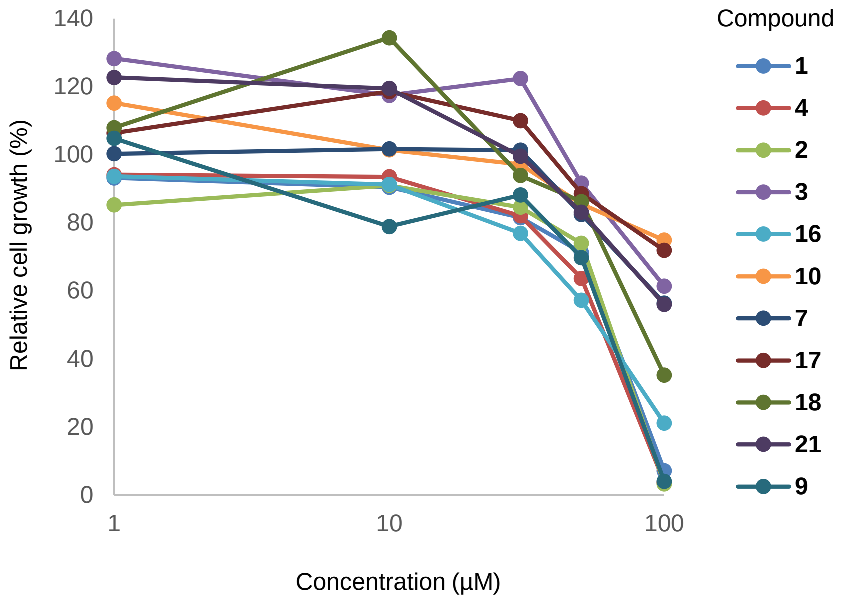


**Supplementary Fig. 7. Cytotoxicity evaluation by MTT assay.** Graphical representation of the vitality variation of Caco-2 cells with different concentrations of dodecyl *O*-/*S*- and *C*-glycosides.

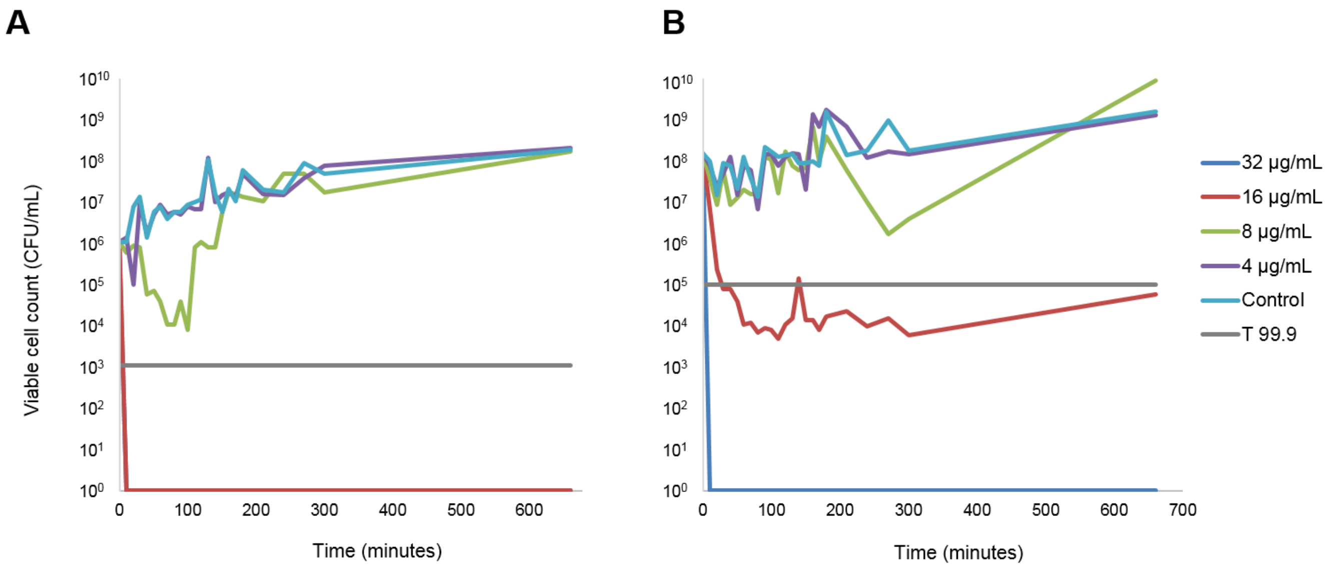


**Supplementary Fig. 8. Time-killing curve of compound 1**. Grafical representation of the time-kill assay performed with *B. cereus* ATCC 14579 strain in the presence of compound **1** at a starting inoculum of (A) $10^6$ CFU $mL^{-1}$ (B) $10^8$ CFU $mL^{-1}$.

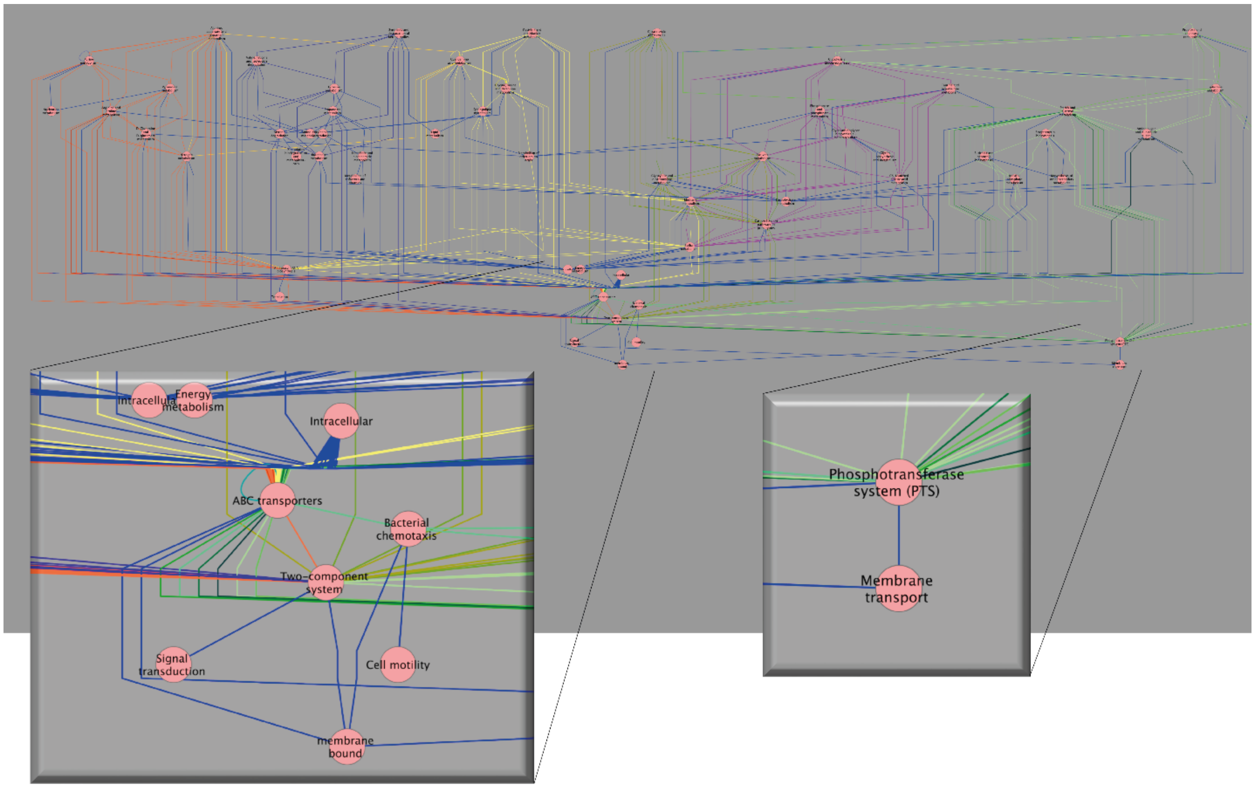


**Supplementary Fig. 9. Details of the metabolic reconstruction of the model strain *B. cereus* ATCC 14579 affected by compound 1**. Functional analysis was established from the hierarchical graph theoretic network reconstruction based on the bivariate matrix of filtered nonparametric association coefficients, between the different analysed carbon sources obtained from phenotypic microarray assay (n = 92); eigenvalues were converted to edges (n = 512). Information enrichment was performed by superimposition of the pathways available for *Bacillus cereus strain ATCC 14579* at Kyoto Encyclopedia of Genes and Genomes database (KEGG). The attributes were assigned according Gene Ontology Classification (biological process, cellular component, molecular function) for each molecular identity found to be associated to action of compound **1**. The nodes represent metabolic pathways and/or components found to be impaired by the action of compound **1**. Coloured lines represent functional correlations between the nodes, namely: (from left to right) i) orange: amino acid metabolism (asparagine and proline derivatives) ii) violet: monocarboxylic acids metabolism iii) yellow: amino acid metabolism (glycine, serine and threonine derivatives) iv) dark green: malic acid and derivatives metabolism v) purple: pyruvate metabolism vi) light green: glycoside metabolism. Lines in blue represent cellular localization (i.e: intracellular or membrane-bound components) or a biological process (e.g.: membrane transport; cell motility or signal transduction). The background image presents the overview of the entire metabolic reconstruction map. The boxes highlight the main functional aggregation hubs in the network, namely: Phosphotransferase systems (PTS), ATP-binding cassette (ABC) transporter systems as well as two-component systems. All the identities associated to these functional hubs are classified as cytoplasmic membrane bound components (blue lines).

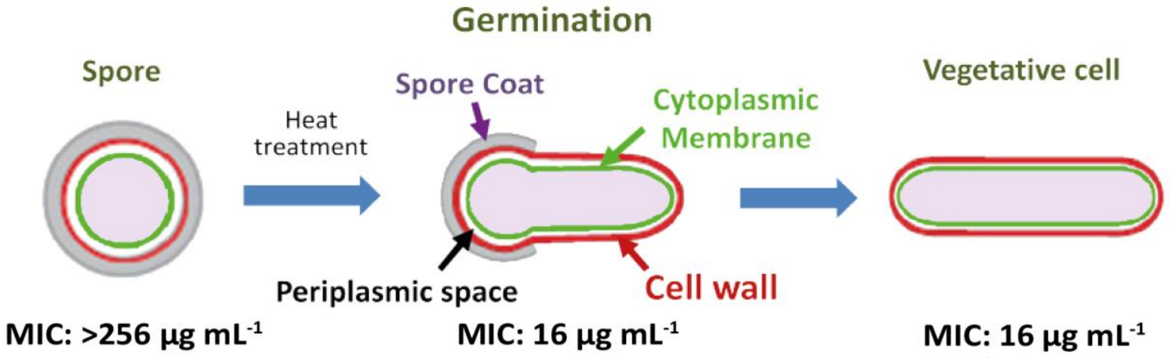


**Supplementary Fig. 10. Depiction of compound 1 activity after spore germination**. Bacterial spores of *B. cereus* ATCC 14579 were produced in sporulation media CCY, purified. Germination was induced by heat shock treatment in germination AGFK buffer. In each of these steps the bacterial spores were submitted to the action of compound **1**, yielding the MIC values shown above and on Supplementary Table 7. No bioactivity was found in the presence of completely formed spore coat, but, upon heat treatment, fissures in the coat uncover the cytoplasmic membrane to the action of compound **1**, resulting in MIC values equal to those observed for vegetative cells.

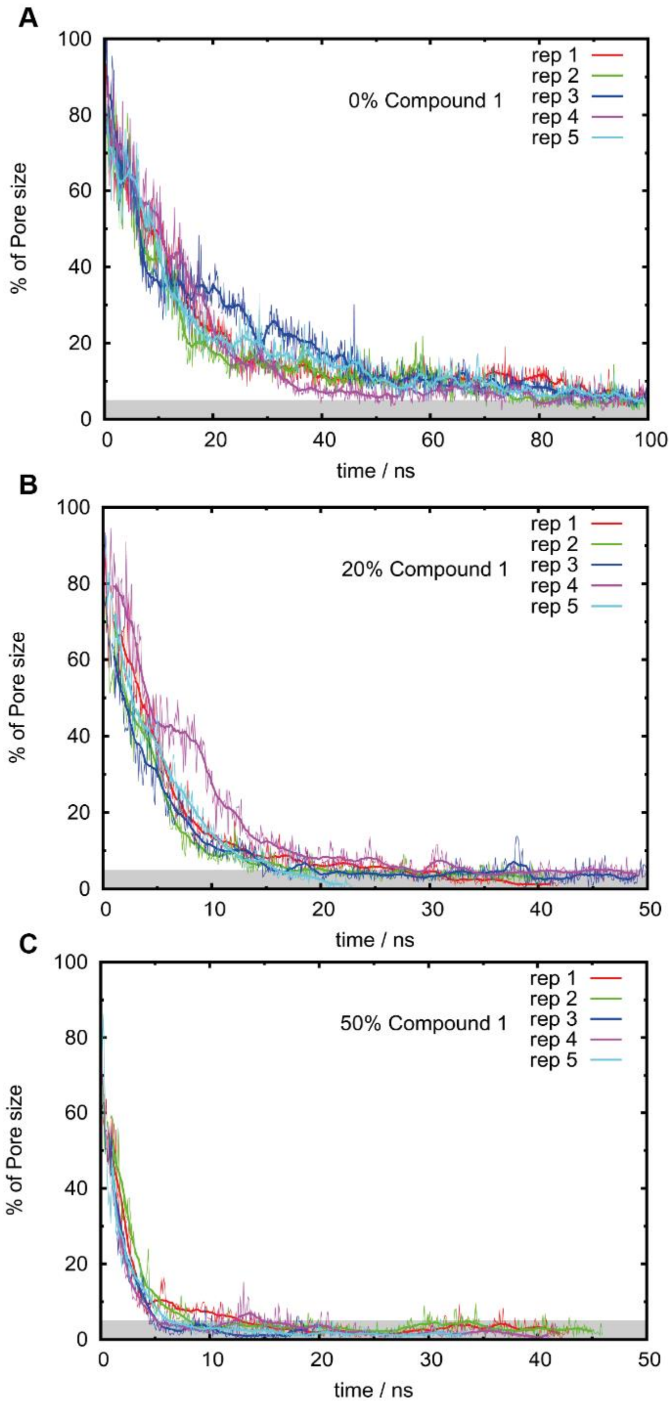


**Supplementary Fig. 11. Estimated size of several pre-formed membrane pores over simulation time without external surface tension.** (A) Pure DMPC (control); (B) A mixture containing compound **1** (20%); (C) A mixture containing compound **1** (50%). The graphic shows the results of five replicates for each system. Pore size was estimated using the number of water molecules in the interior of the transmembrane cavity. A full pore corresponds to a region with ~1800 water molecules between leaflets. The gray region represents very small pore sizes and, in most cases, complete closure of the pore. A floating window average (2 ns) is also shown for clarity.

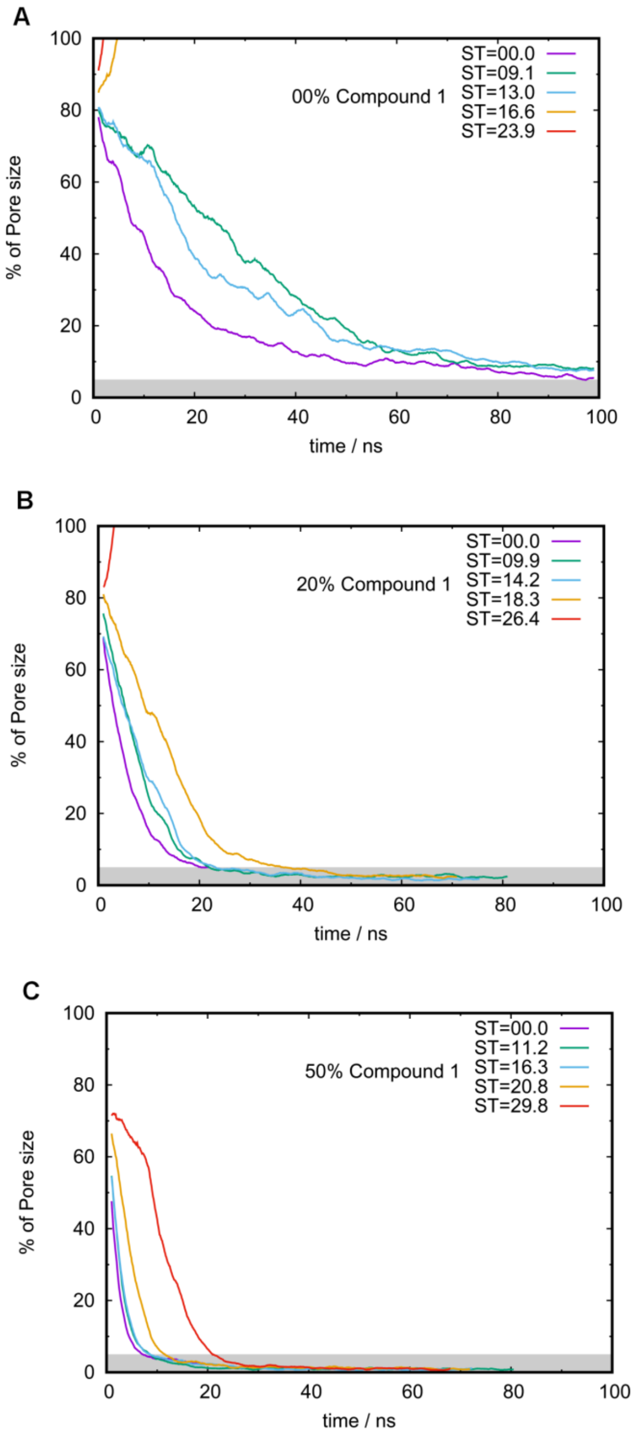


**Supplementary Fig. 12. Estimated size of several pre-formed membrane pores over simulation time and at different applied surface tension (ST) values (0 – ~30 dyn cm$^{-1}$).** (A) Pure DMPC (control); (B) A mixture containing compound **1** (20%); (C) A mixture containing compound **1** (50%). Pore size was estimated using the number of water molecules in the interior of the transmembrane cavity. A full pore corresponds to a region with ~1800 water molecules between leaflets. The gray region represents very small pore sizes and, in most cases, complete closure of the pore. At higher ST values and low compound **1** concentrations, the pores size increases and the lipid bilayer becomes too unstable. The data is shown as a floating window average (2 ns) for clarity.

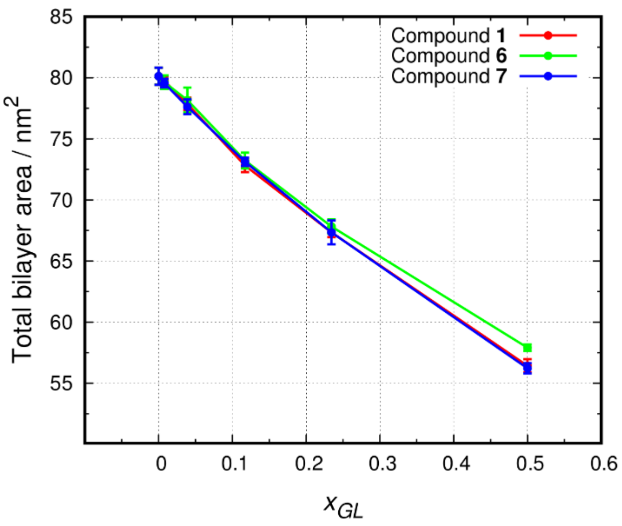


**Supplementary Fig. 13. Total cross-sectional area of the bilayer as a function of the glycoside molar fraction for the simulated binary mixture systems.** Error bars were computed as the correlation-corrected standard error in the mean averaging over three independent replicates.

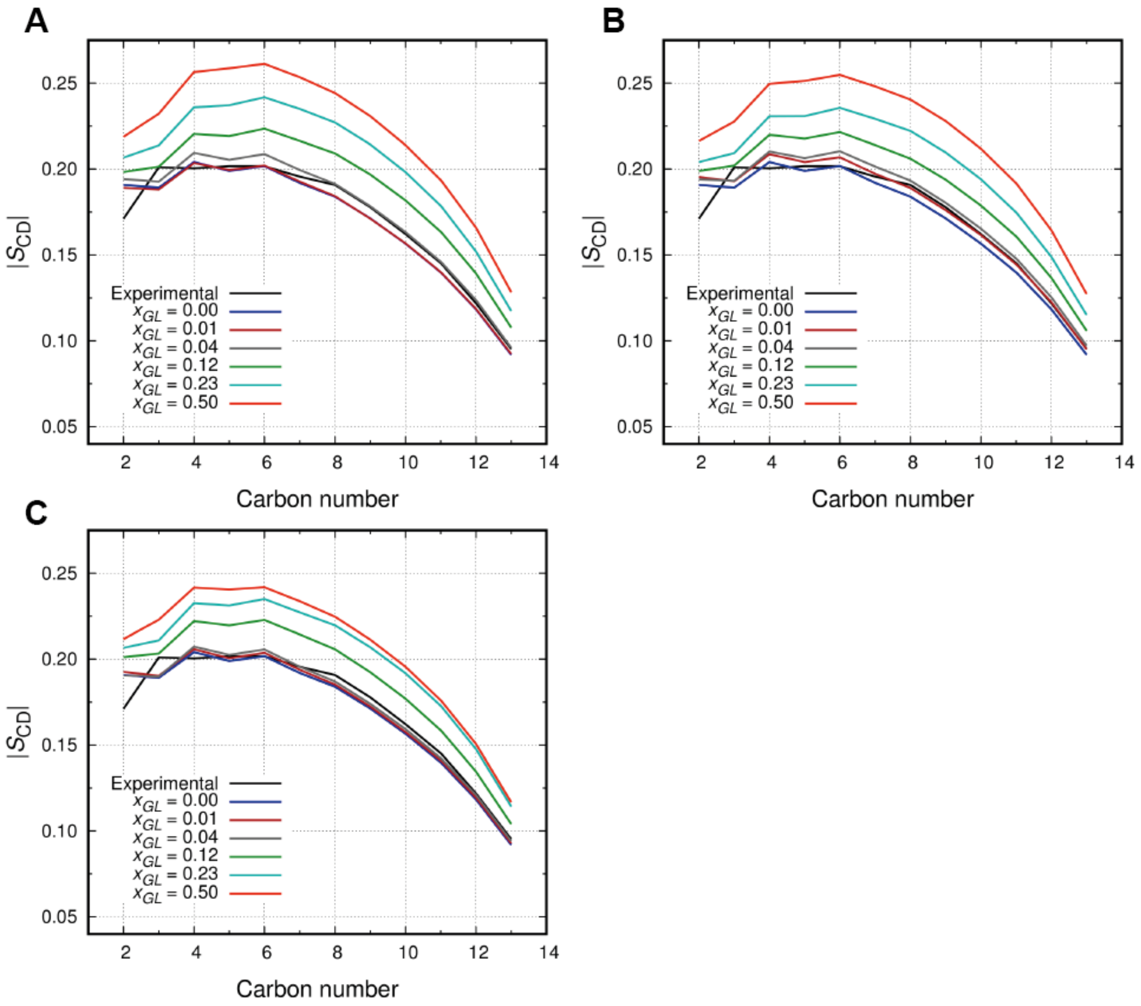


**Supplementary Fig. 14. Deuterium order parameter profiles averaged over both *sn*-1 and *sn*-2 acyl chains of DMPC for the simulated binary mixtures at different glycoside molar fractions.** (A) GL = compound **1**; (B) GL = compound **7**; (C) GL = compound **6**. Note that the $|S_{CD}|$ values computed for the control simulation ($x_{GL}$ = 0) greatly reproduce experimental results reported in the literature for pure DMPC (when linearly interpolated for $T$=310K)[2].

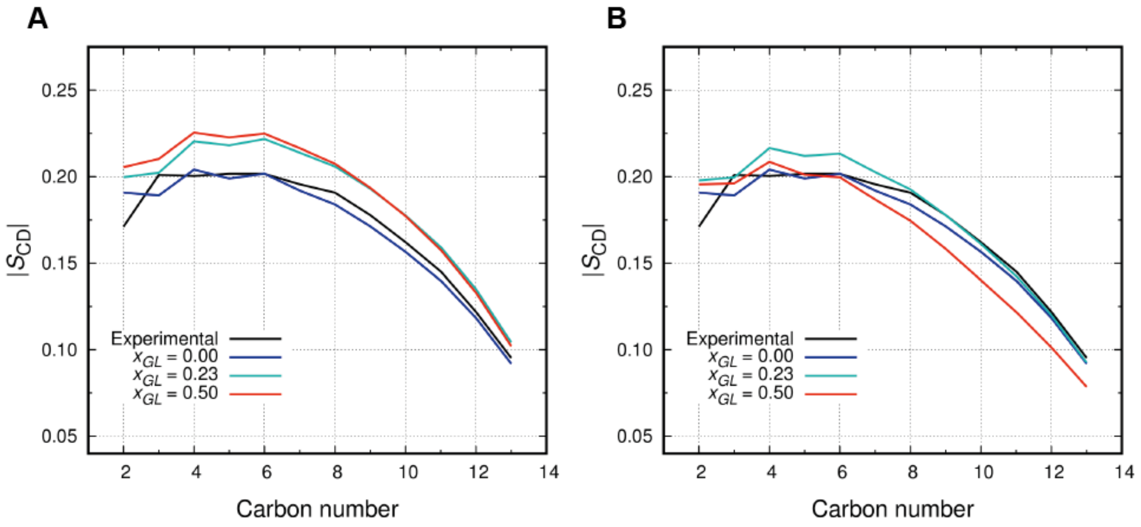


**Supplementary Fig. 15. Deuterium order parameter profiles averaged over both *sn*-1 and *sn*-2 acyl chains of DMPC for the simulated binary mixtures at different glycoside molar fractions.** A) GL = dodecyl β-D-glucopyranoside (DG); B) GL = octyl β-D-glucopyranoside (OG). Note that the $|S_{CD}|$ values computed for the control simulation ($x_{GL}$ = 0) greatly reproduce experimental results reported in the literature for pure DMPC (when linearly interpolated for $T$=310K)[2].

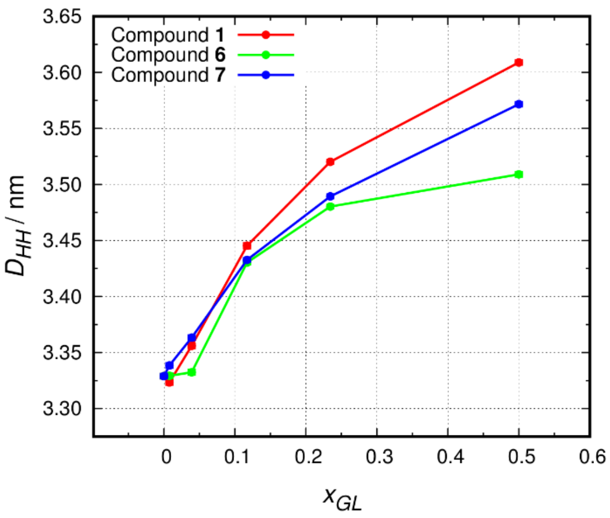


**Supplementary Fig. 16. Bilayer thickness, $D_{HH}$.** It is defined as the average distance between headgroup phosphorus atoms in the *z*-component, both as a function of the glycoside molar fraction. A value of 3.329 ± 0.002 nm was obtained for pure DMPC bilayer simulation which is also in excellent agreement with experimental data (3.49 ± 0.07 nm at 30 ºC and 3.22 ± 0.07 nm at 50 ºC)[3]. The average bilayer thickness increases when the glycoside molar fraction is increased, similarly to the results obtained for the order parameter. Error bars were computed as the correlation-corrected standard error in the mean averaging over three independent replicates.

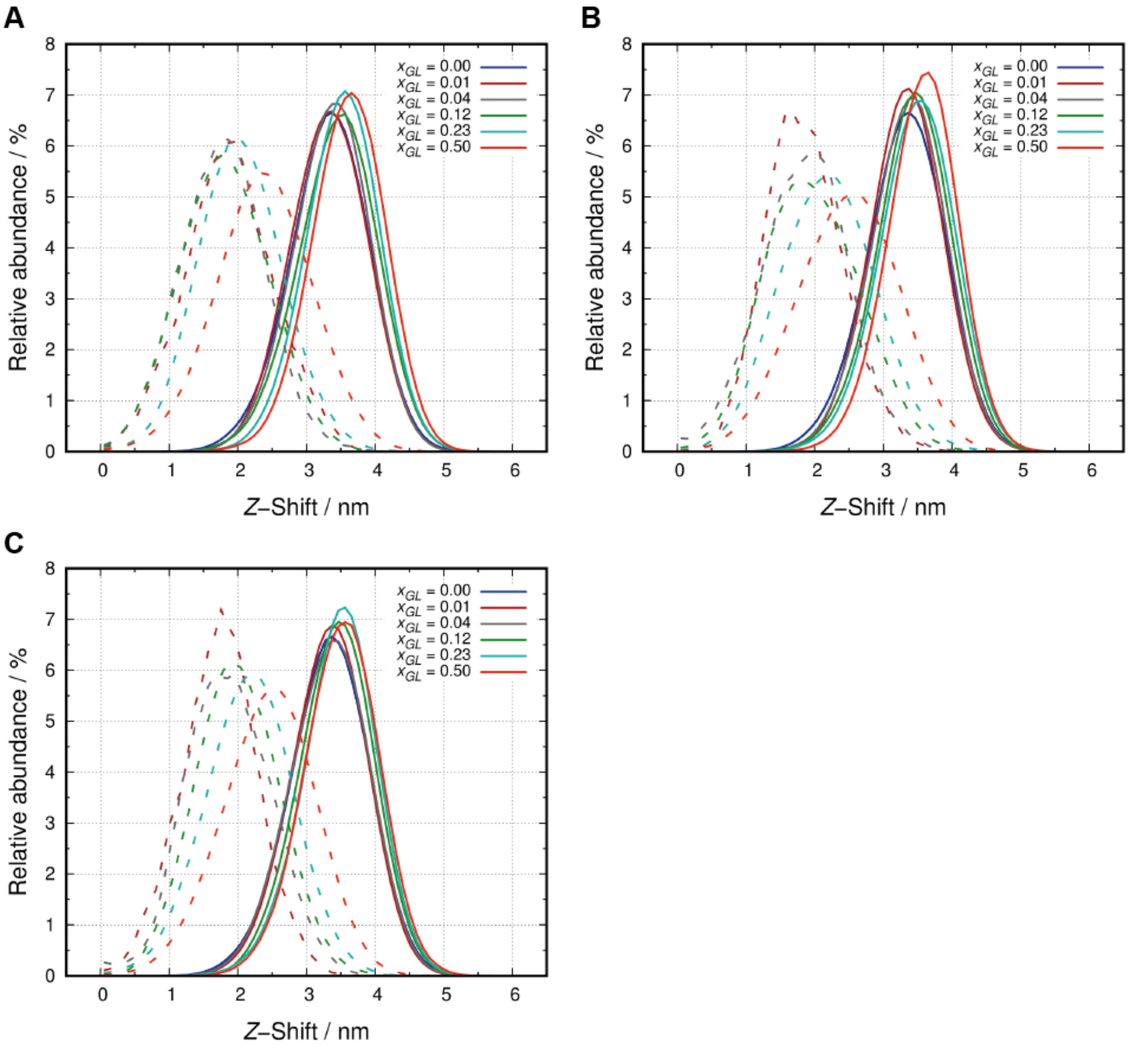


**Supplementary Fig. 17. Histograms of twice the distance to the bilayer center.** For DMPC, all phospholipid phosphorus atoms were used (solid lines) and these distances correspond to $D_{HH}$, while for glycosides, the endocyclic oxygen atoms were used (dashed lines), and the distances correspond to their preferred regions**.** A) GL = compound **1**; B) GL = compound **7**; C) GL = compound **6**. The distribution of glycoside headgroups along the bilayer normal is also dependent on glycoside molar fraction: at higher molar fractions, glycoside headgroups populate outer regions of the bilayer, particularly in the case of more polar headgroups, indicating that the loading capacity of the bilayer is limited.

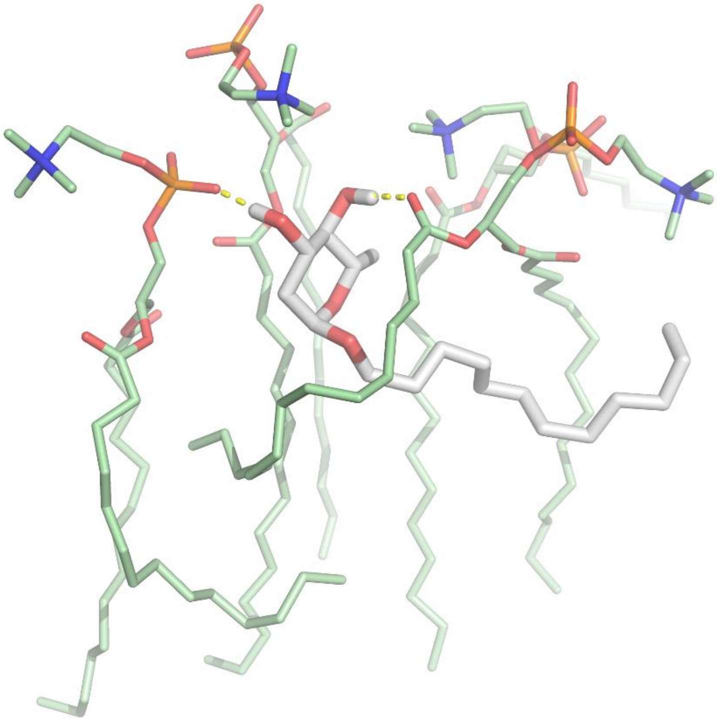

**Supplementary Fig. 18. Representative snapshot of compound 1 surrounded by neighboring DMPC molecules in a bilayer environment**. Conformation taken from one of the simulations ($x_{GL}$ = 0.23) where the glycoside molecule is typically inserted below phospholipid phosphates, interacting with neighboring phospholipids by establishing hydrogen bonds with different acceptors. The glycoside molecule is shown in light gray sticks (carbon atoms), red sticks (oxygen atoms) and white sticks (hydroxyl group hydrogens), while phospholipid molecules are represented as light green sticks (carbon atoms), red sticks (oxygen atoms), orange sticks (phosphorus) and blue sticks (nitrogen). Hydrogen bond interactions are highlighted in dashed lines. Non-polar hydrogen atoms, water molecules and the remaining lipids have been omitted for clarity.

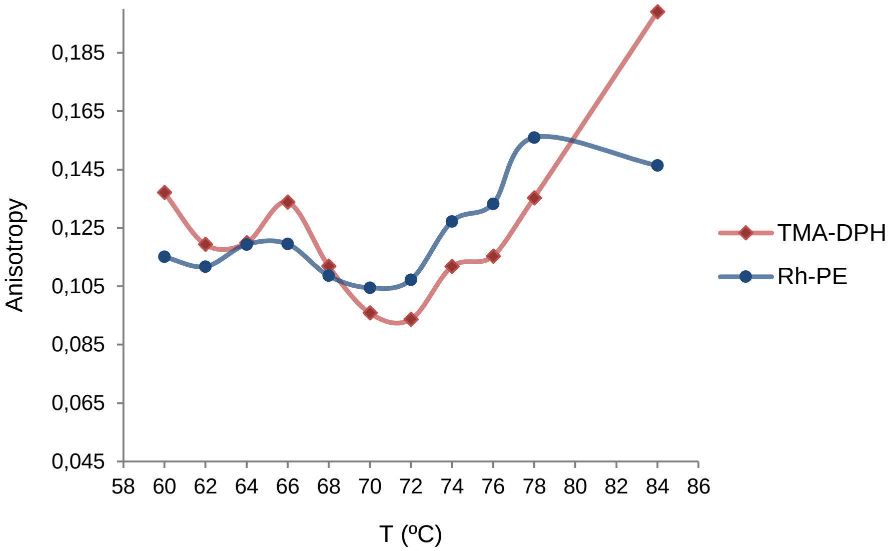


**Supplementary Fig. 19. Thermotropic behaviour of POPE**. Evaluation by steady-state fluorescence anisotropy of TMA-DPH and by Rh-PE.

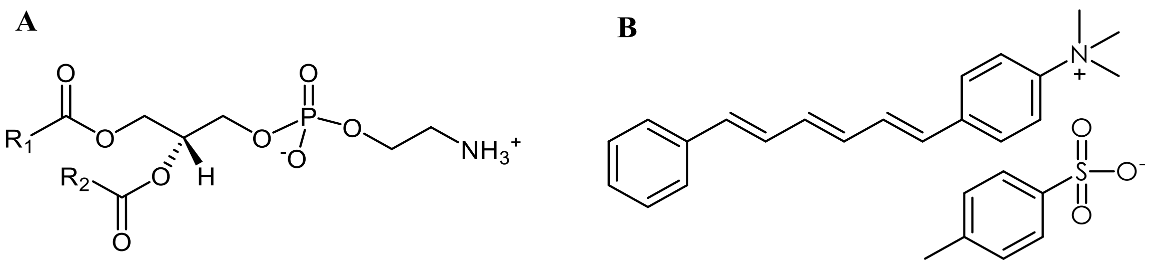


**Supplementary Fig. 20. Structures of lipids and probe used for fluorescence anisotropy measurements.** (A) General structure of phosphatidylethanolamine (PE) lipids; (B) 1-(4-trimethylammoniumphenyl)-6-phenyl-1,3,5-hexatriene *p*-toluenesulfonate (TMA-DPH).

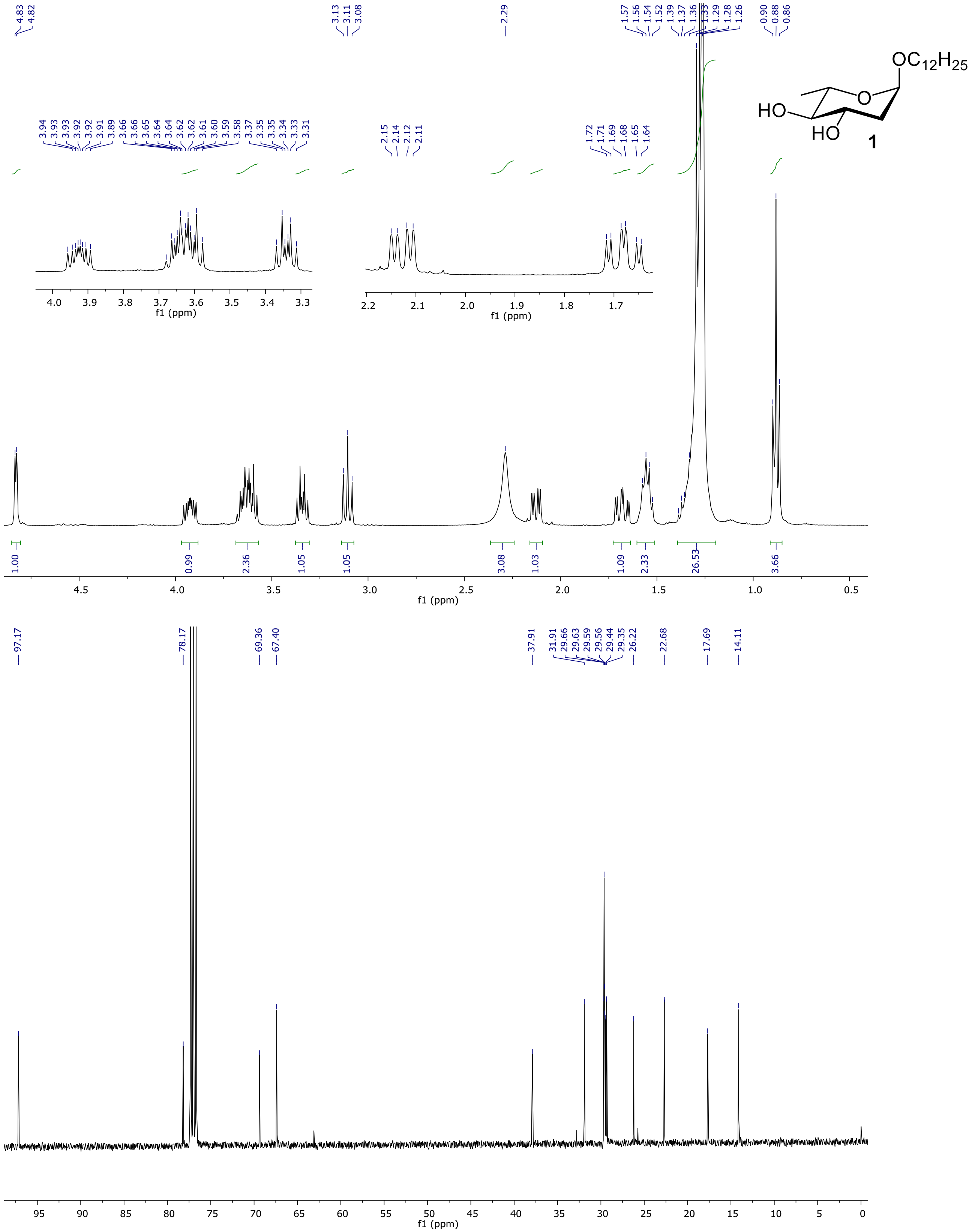


**Supplementary Fig. 21.** $^{1}H$ NMR and $^{13}C$ NMR spectra of compound **1**.

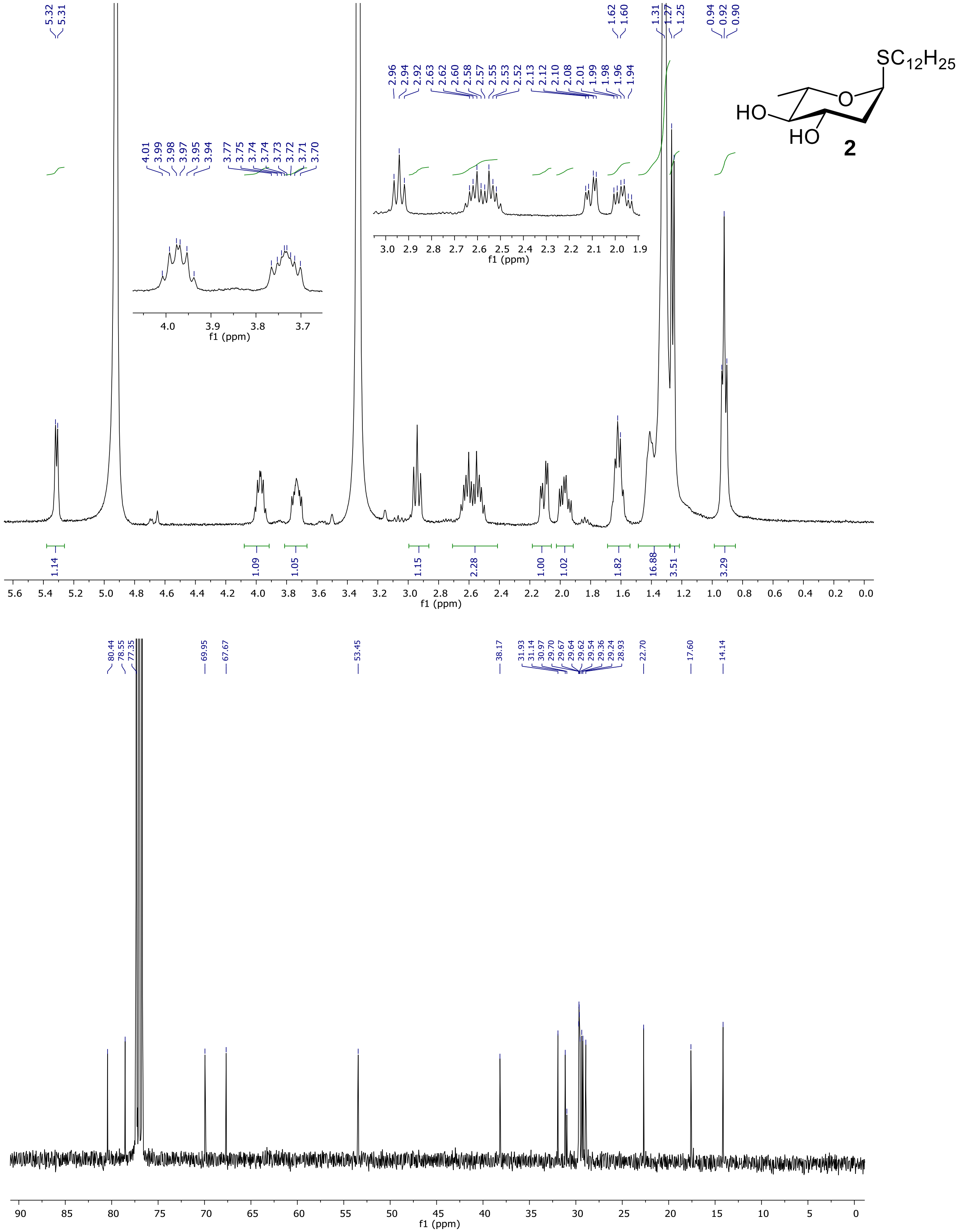


**Supplementary Fig. 22.** $^1$H NMR and $^{13}$C NMR spectra of compound **2**.

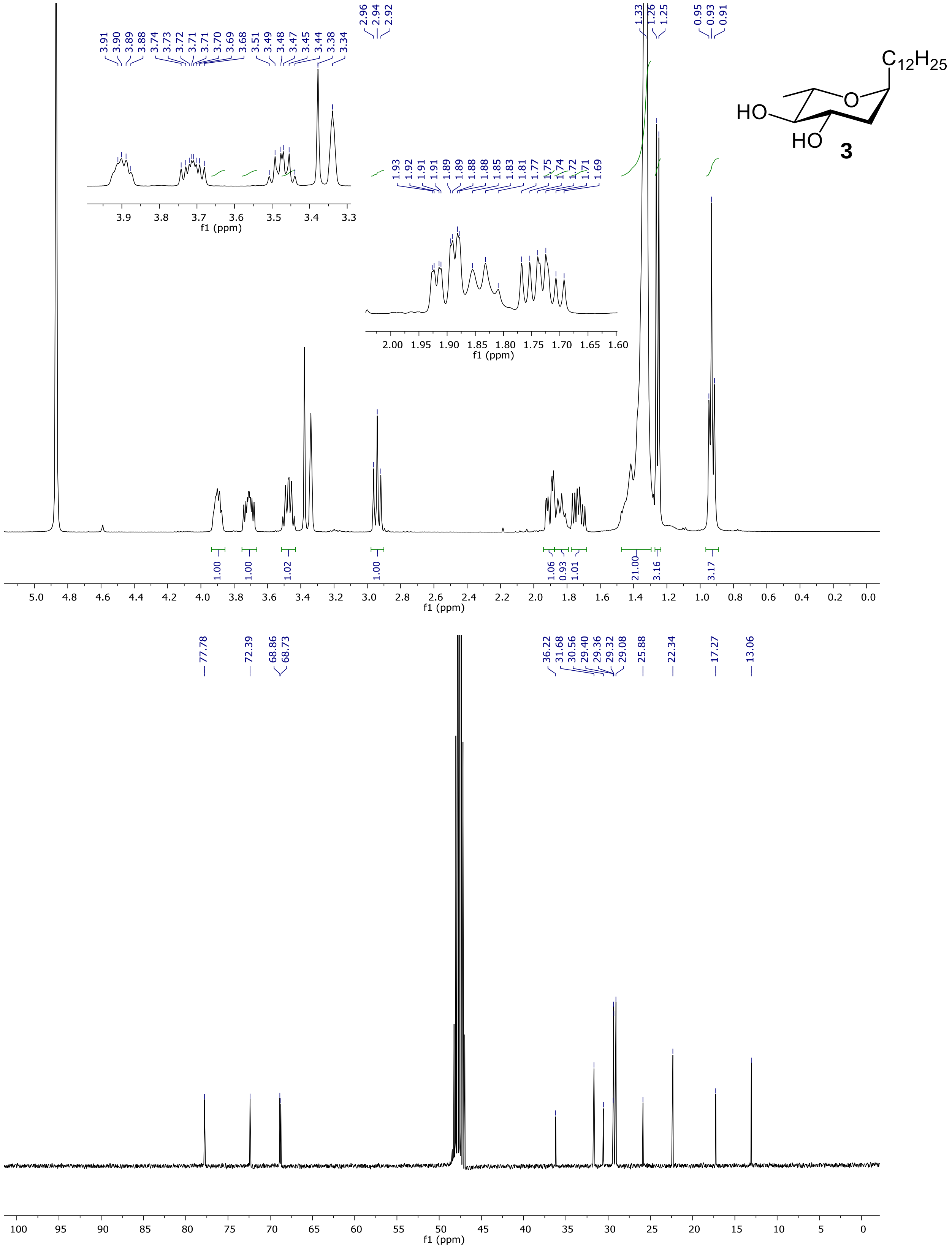


**Supplementary Fig. 23.** $^{1}$H NMR and $^{13}$C NMR spectra of compound **3**.

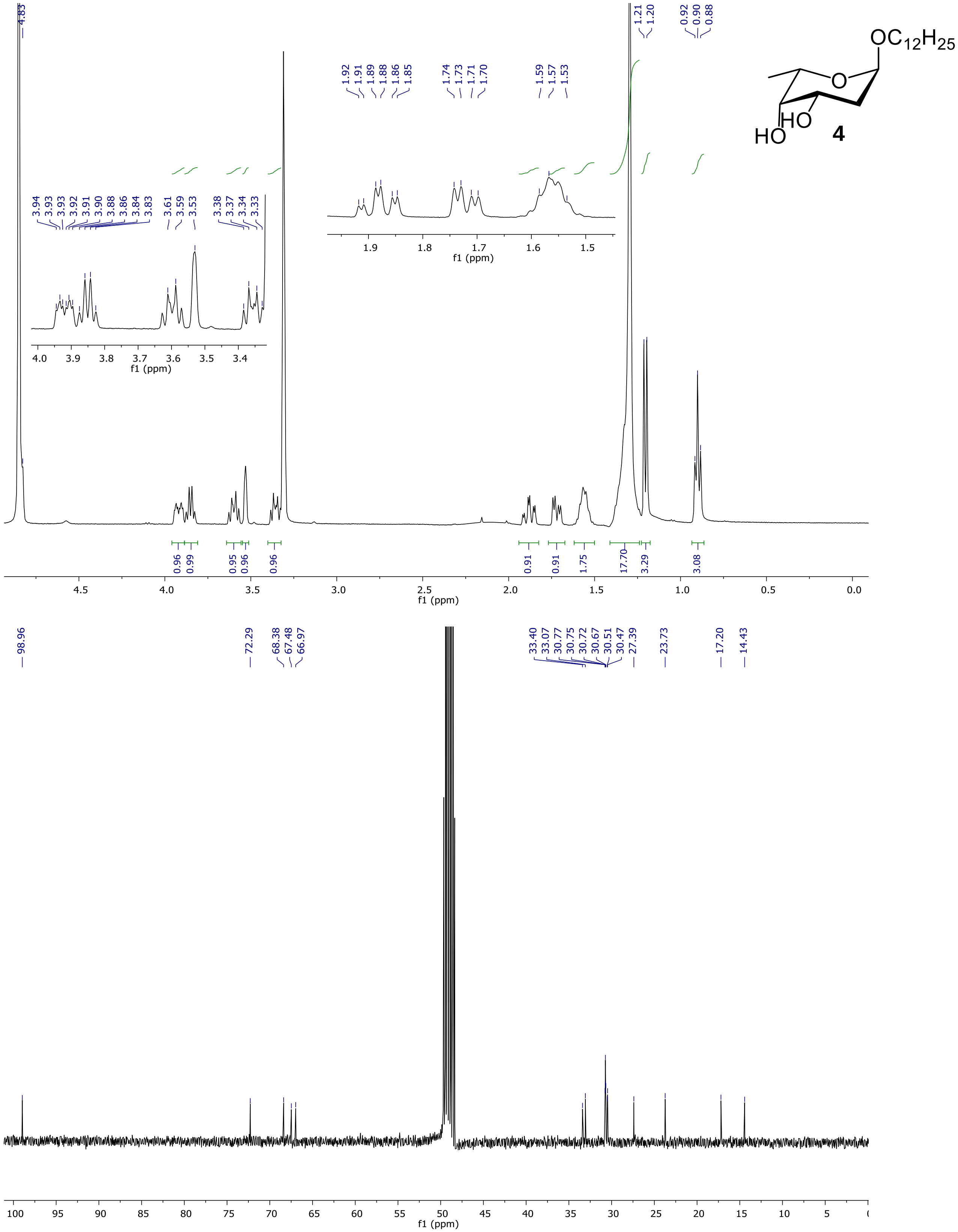


**Supplementary Fig. 24.** $^{1}$H NMR and $^{13}$C NMR spectra of compound **4**.

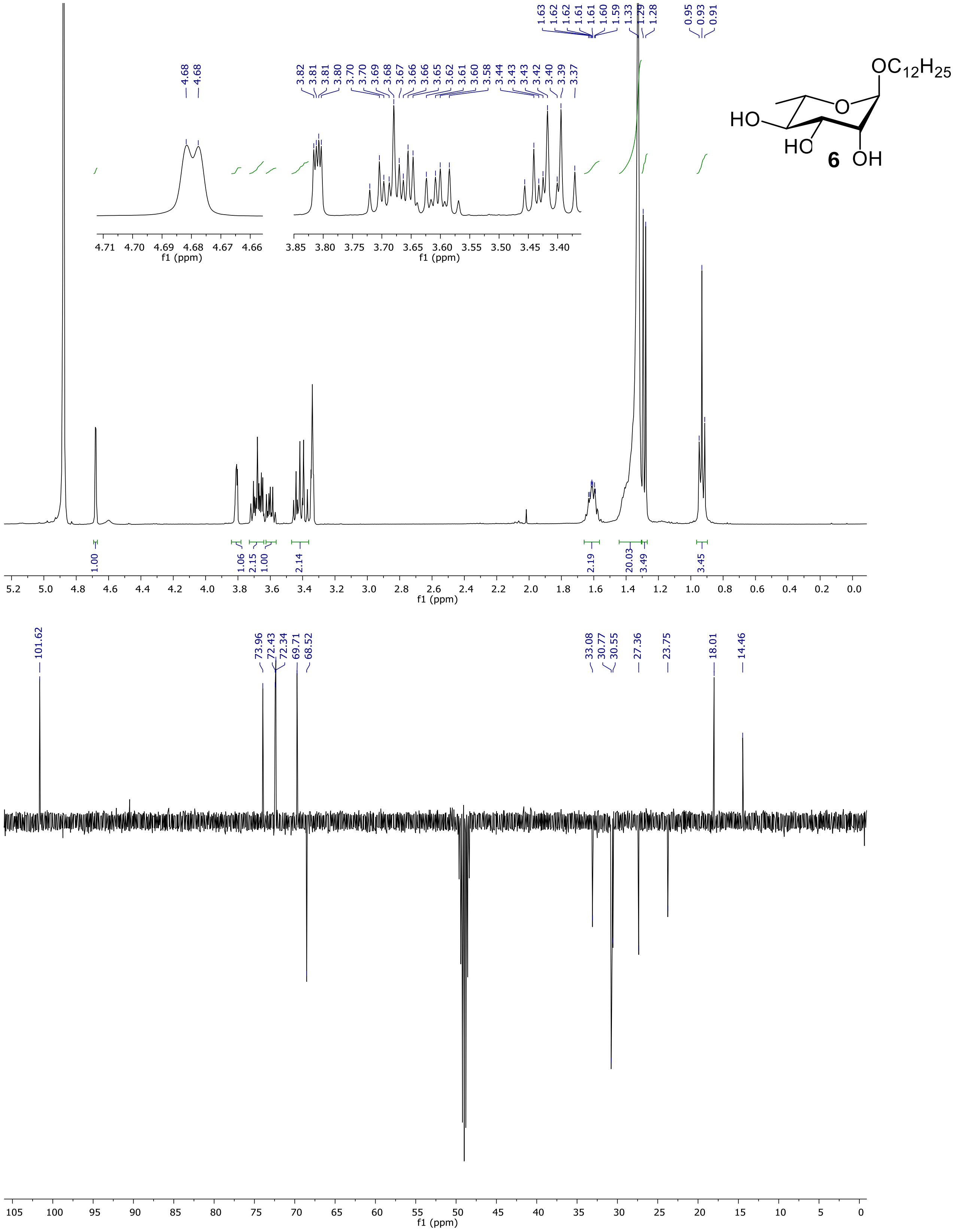


**Supplementary Fig. 25.** $^{1}$H NMR and $^{13}$C NMR spectra of compound **6**.

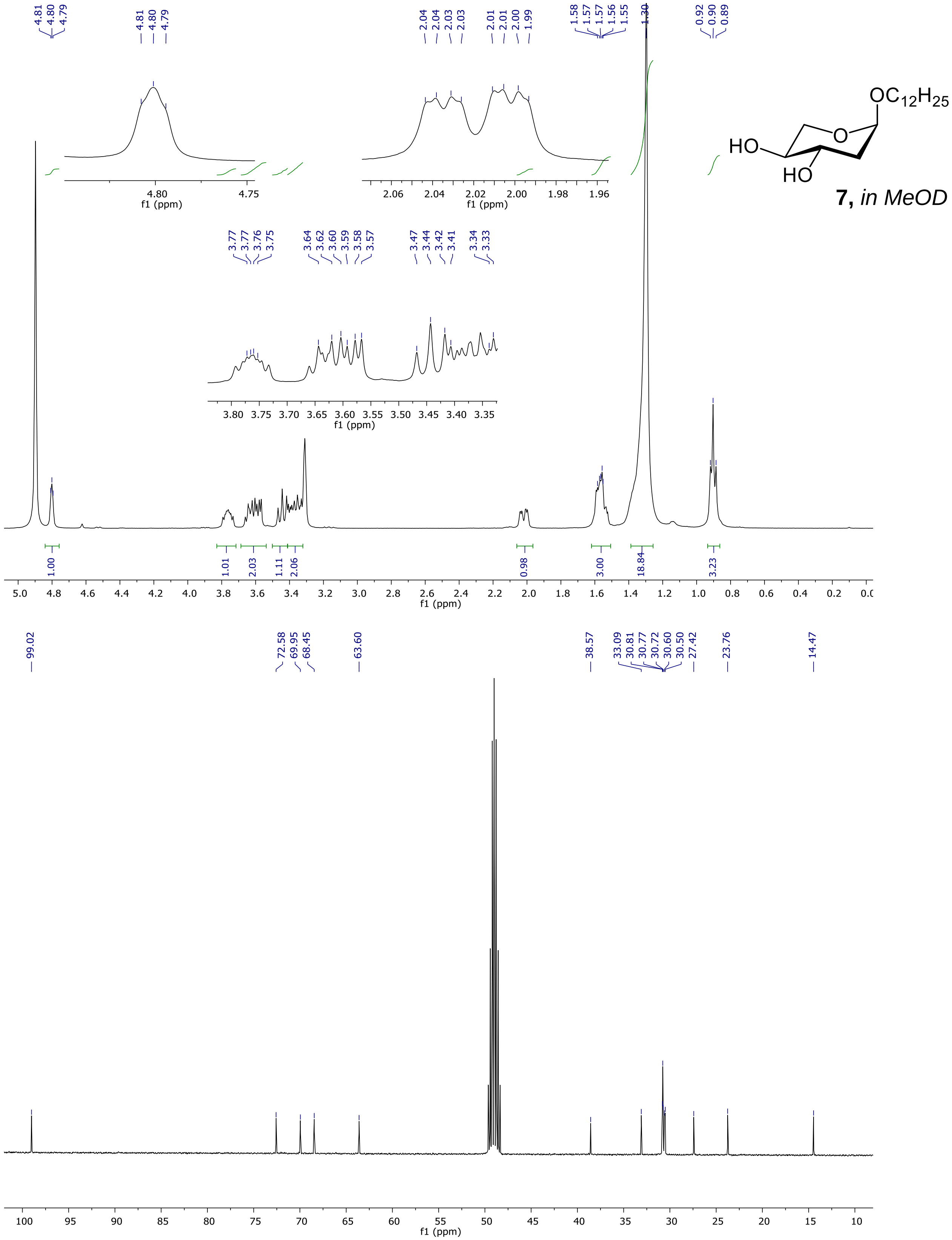


**Supplementary Fig. 26.** $^{1}H$ NMR and $^{13}C$ NMR spectra of compound **7** in MeOD.

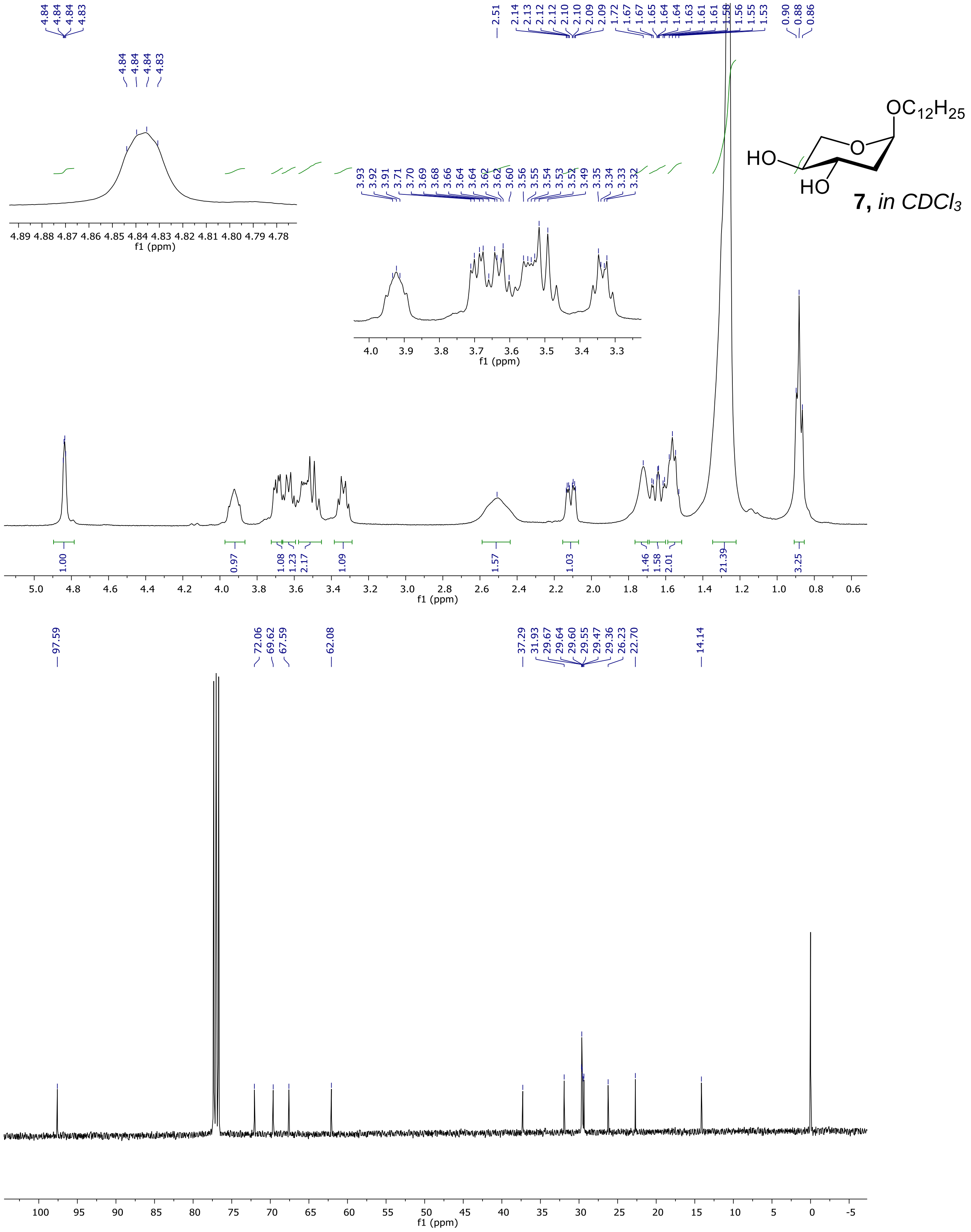


**Supplementary Fig. 27.** $^{1}H$ NMR and $^{13}C$ NMR spectra of compound **7** in $CDCl_3$.

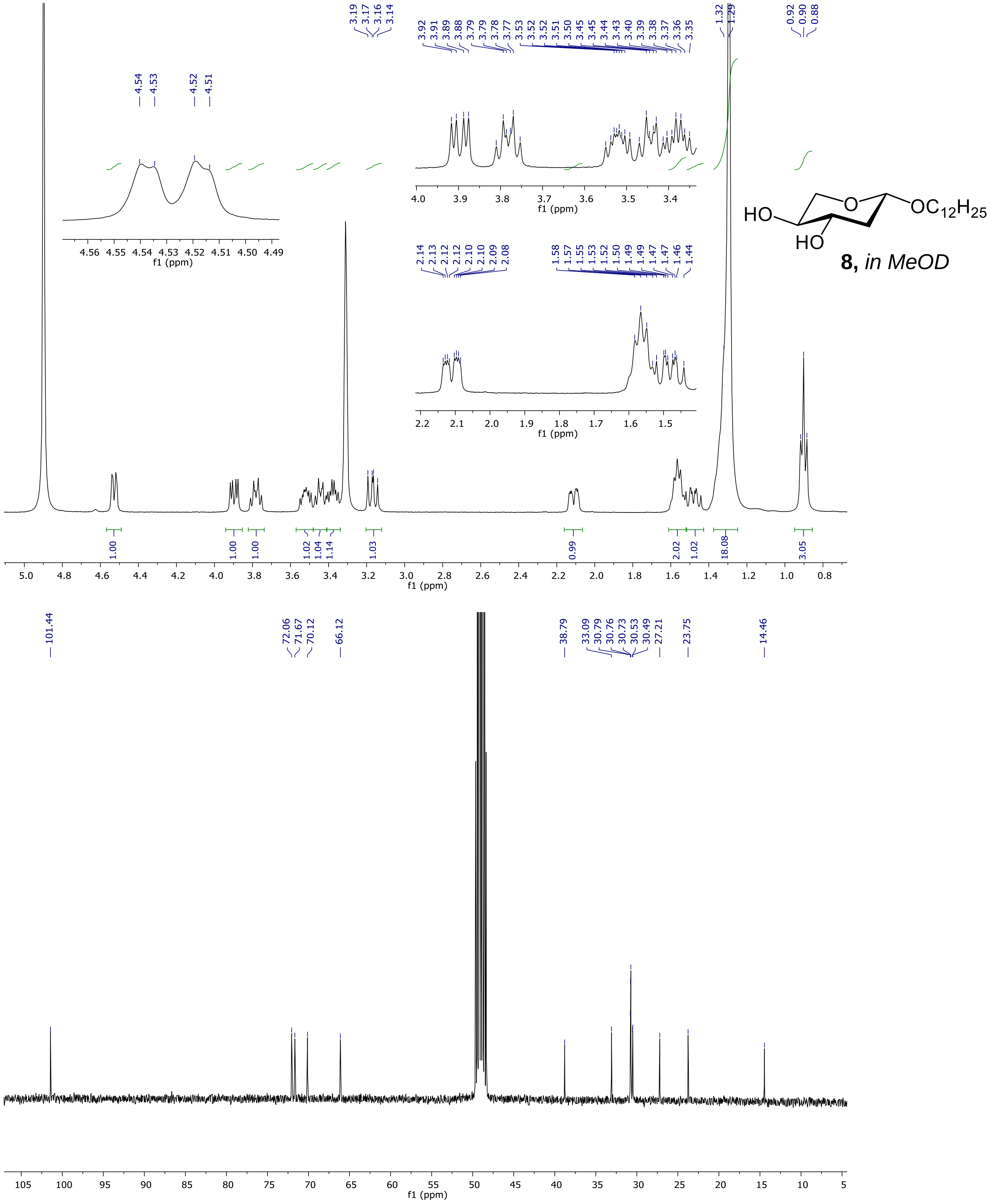


**Supplementary Fig. 28.** $^{1}H$ NMR and $^{13}C$ NMR spectra of compound **8** in MeOD.

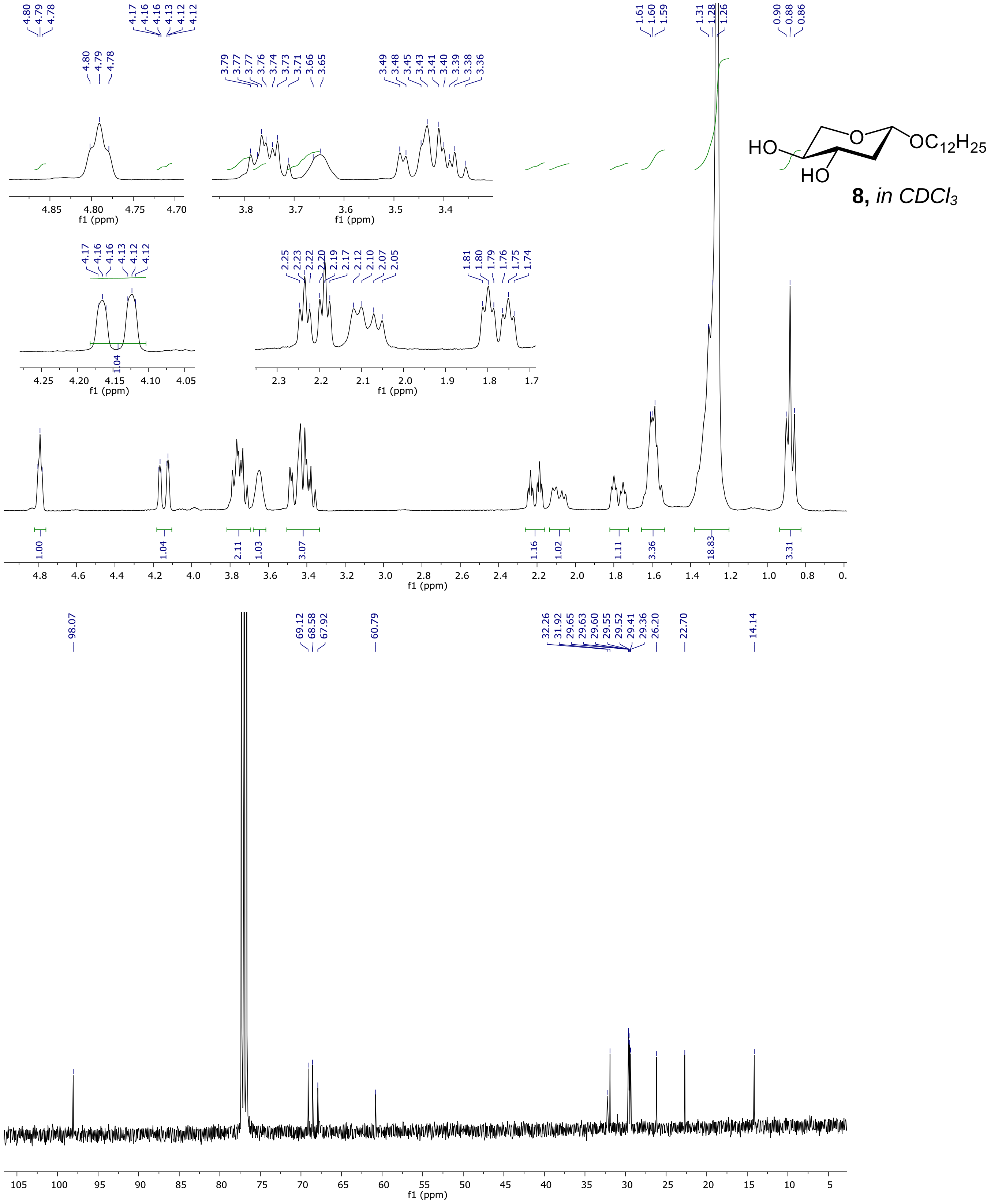


**Supplementary Fig. 29.** $^{1}H$ NMR and $^{13}C$ NMR spectra of compound **8** in $CDCl_3$.

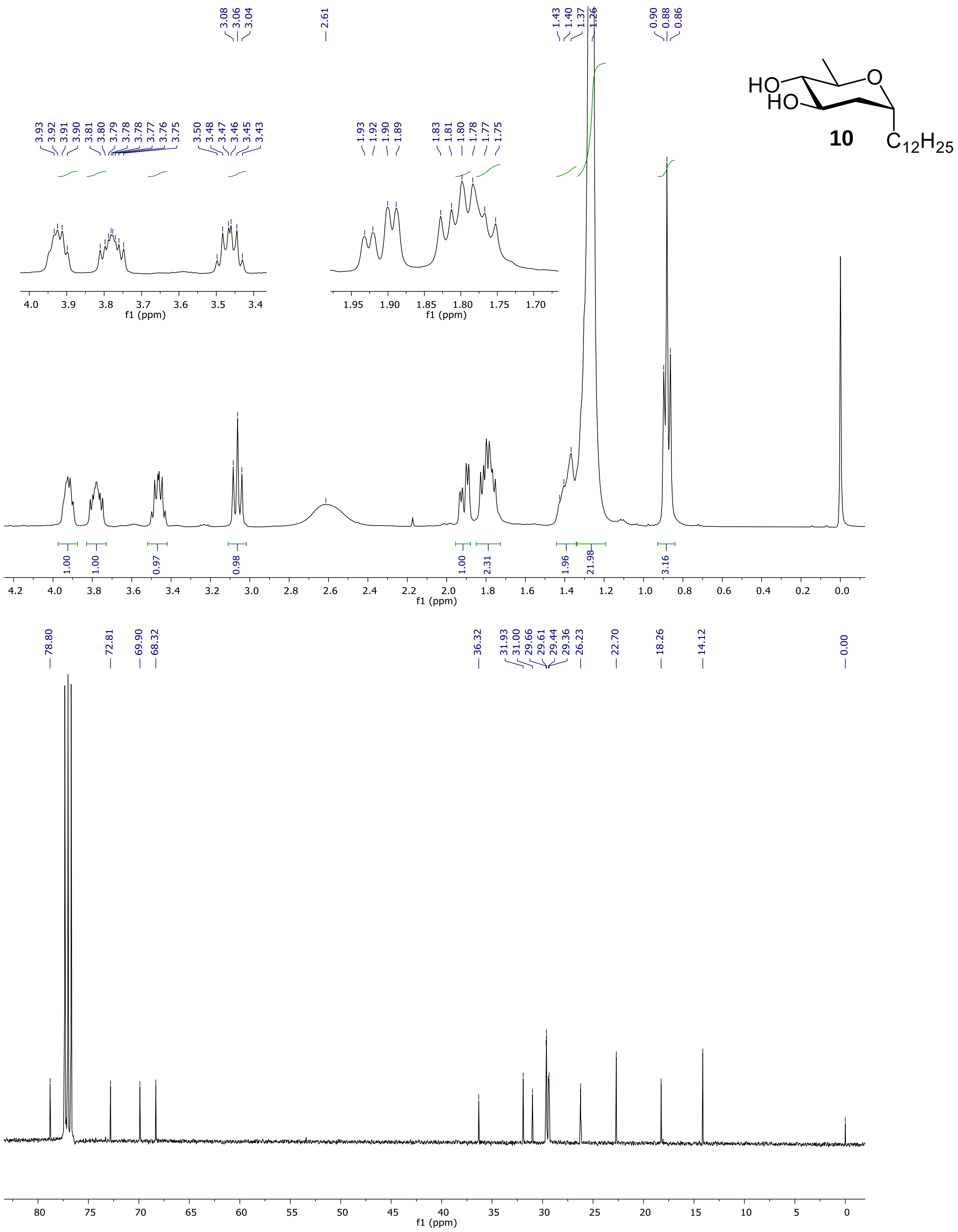

**Supplementary Fig. 30.** $^{1}H$ NMR and $^{13}C$ NMR spectra of compound **10**.

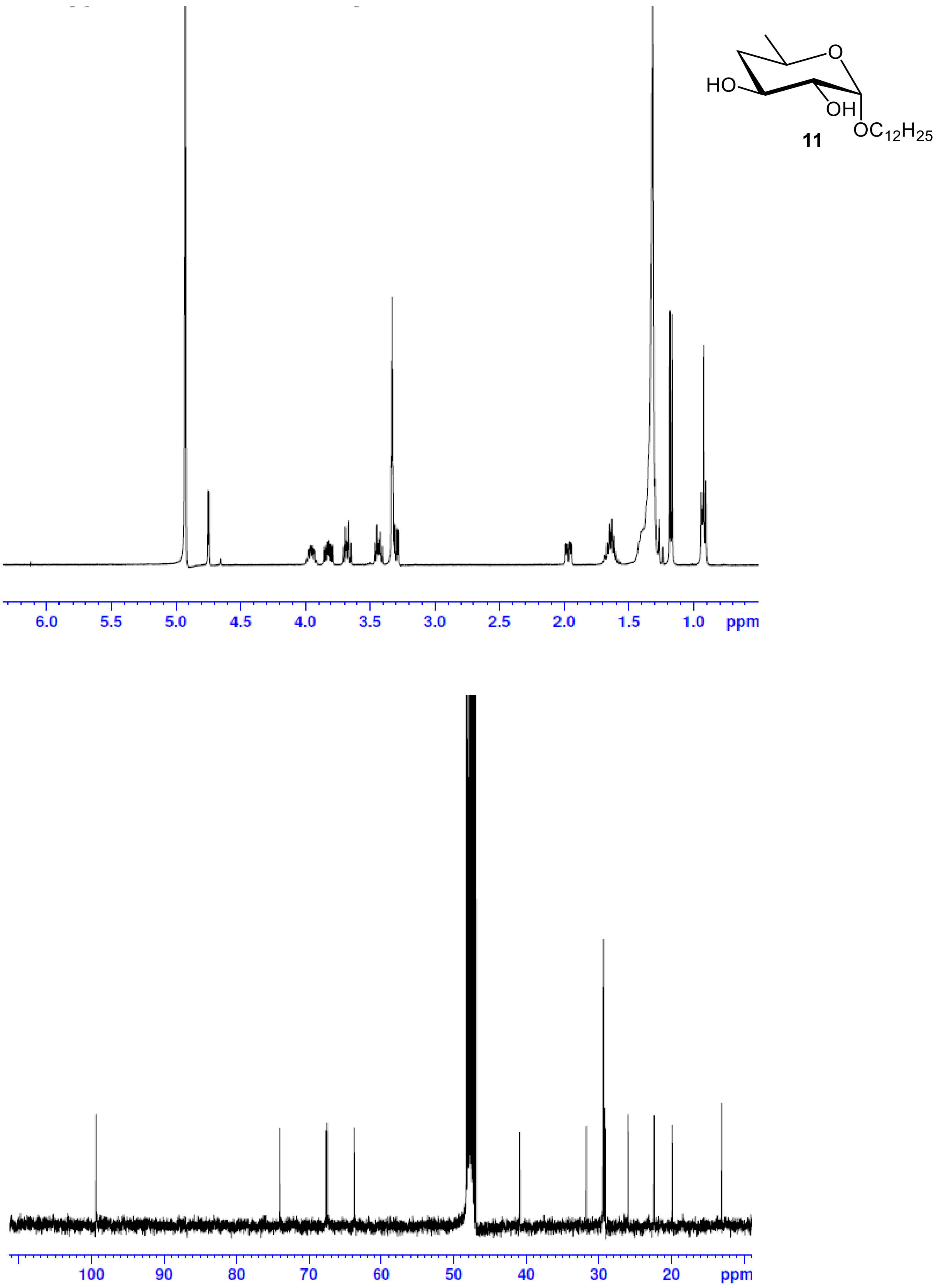


**Supplementary Fig. 31.** $^{1}$H NMR and $^{13}$C NMR spectra of compound **11**.

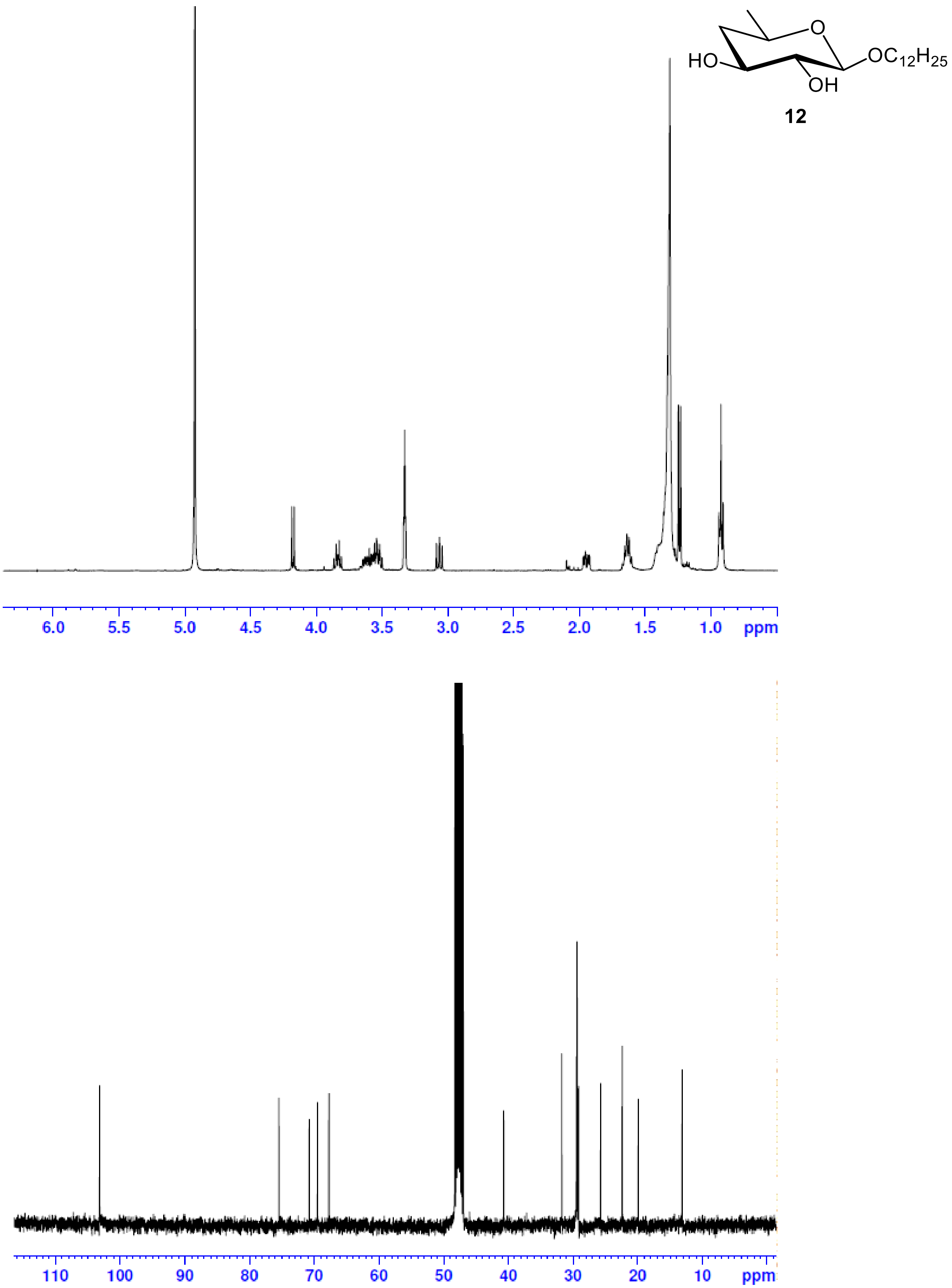


**Supplementary Fig. 32.** $^{1}H$ NMR and $^{13}C$ NMR spectra of compound **12**.

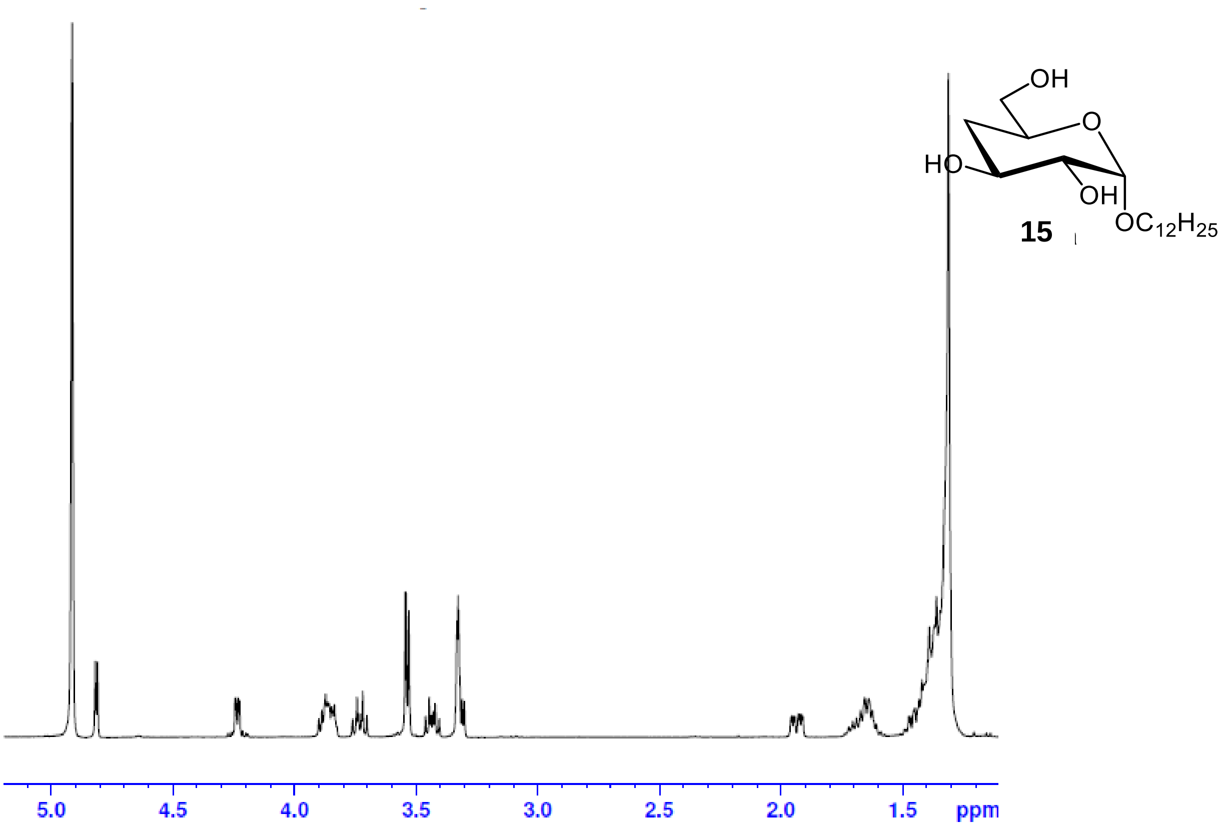


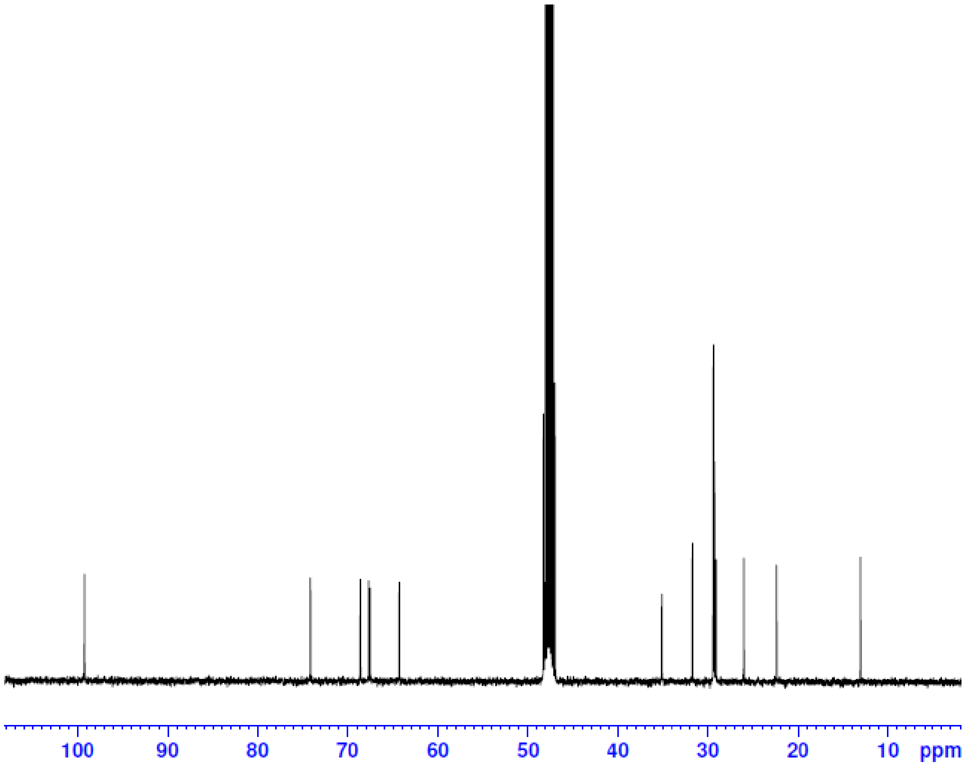


**Supplementary Fig. 33.** $^{1}$H NMR and $^{13}$C NMR spectra of compound **15**.

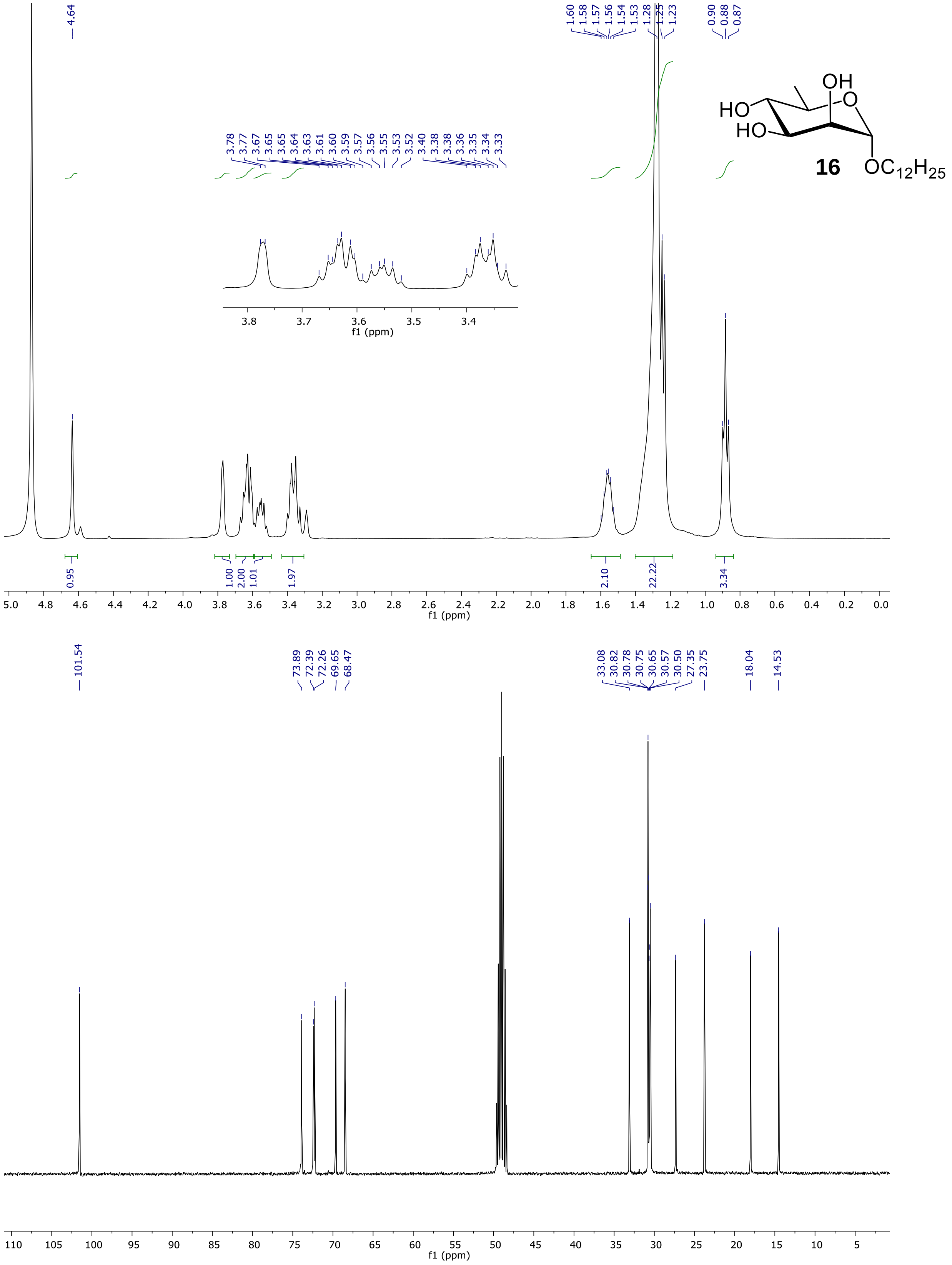


**Supplementary Fig. 34.** $^{1}H$ NMR and $^{13}C$ NMR spectra of compound **16**.

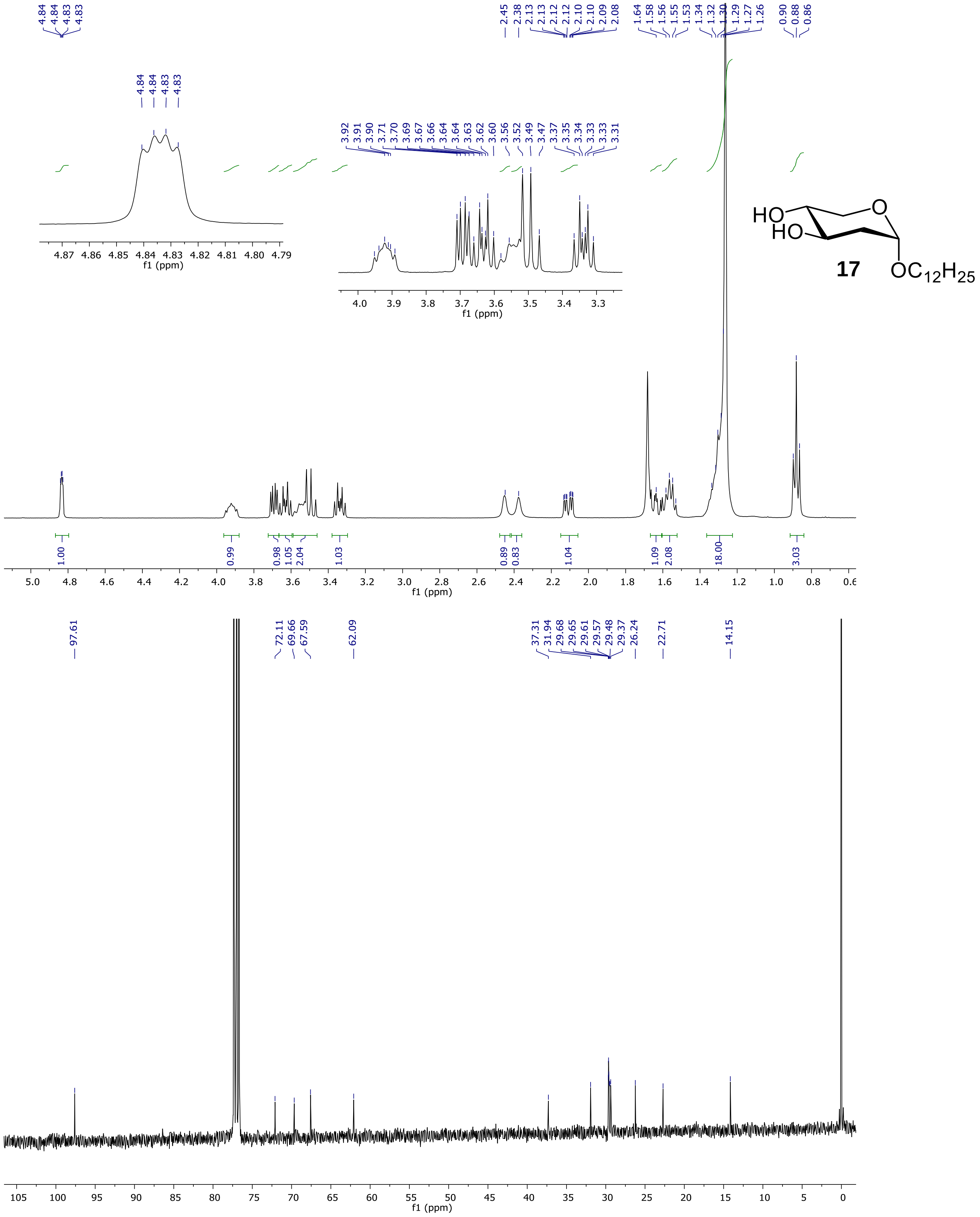


**Supplementary Fig. 35.** $^{1}H$ NMR and $^{13}C$ NMR spectra of compound **17**.

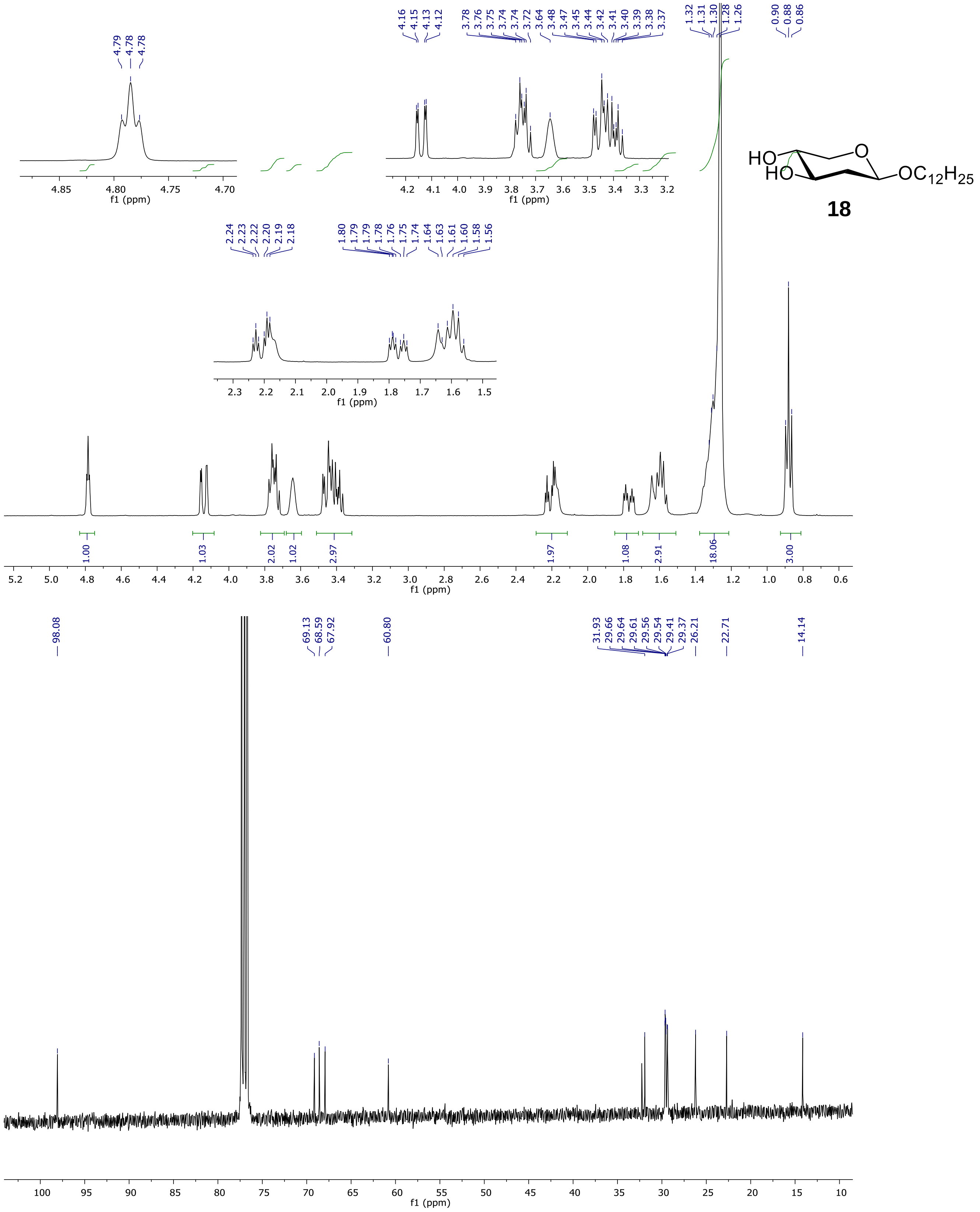


**Supplementary Fig. 36.** $^{1}$H NMR and $^{13}$C NMR spectra of compound **18**.

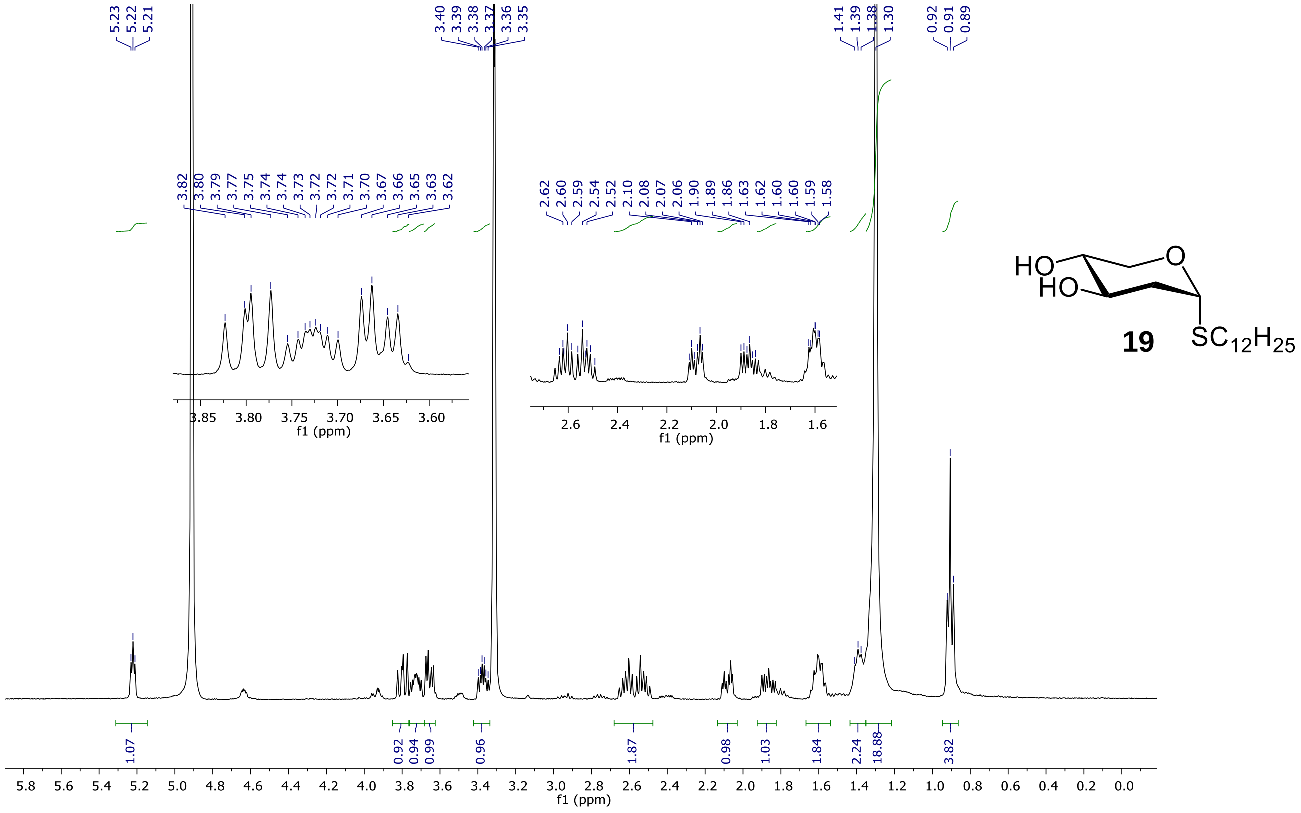

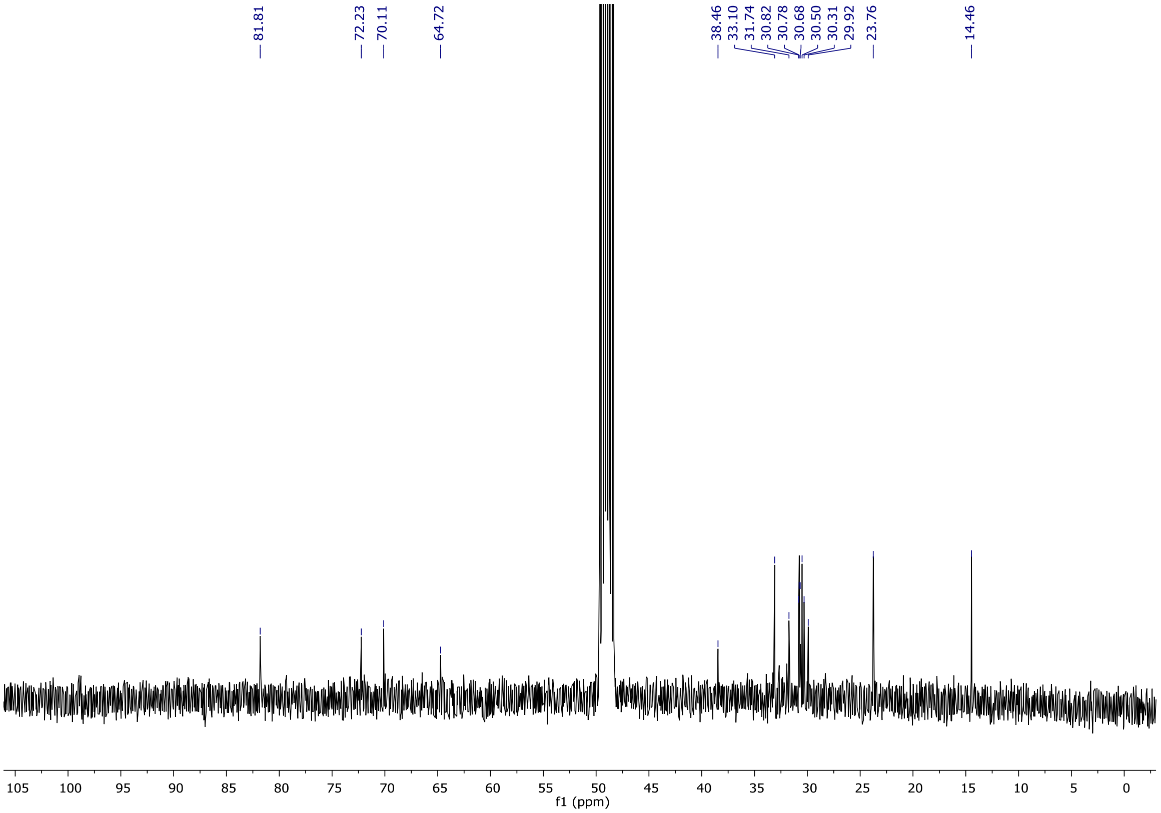

**Supplementary Fig. 37.** $^{1}H$ NMR and $^{13}C$ NMR spectra of compound **19**.

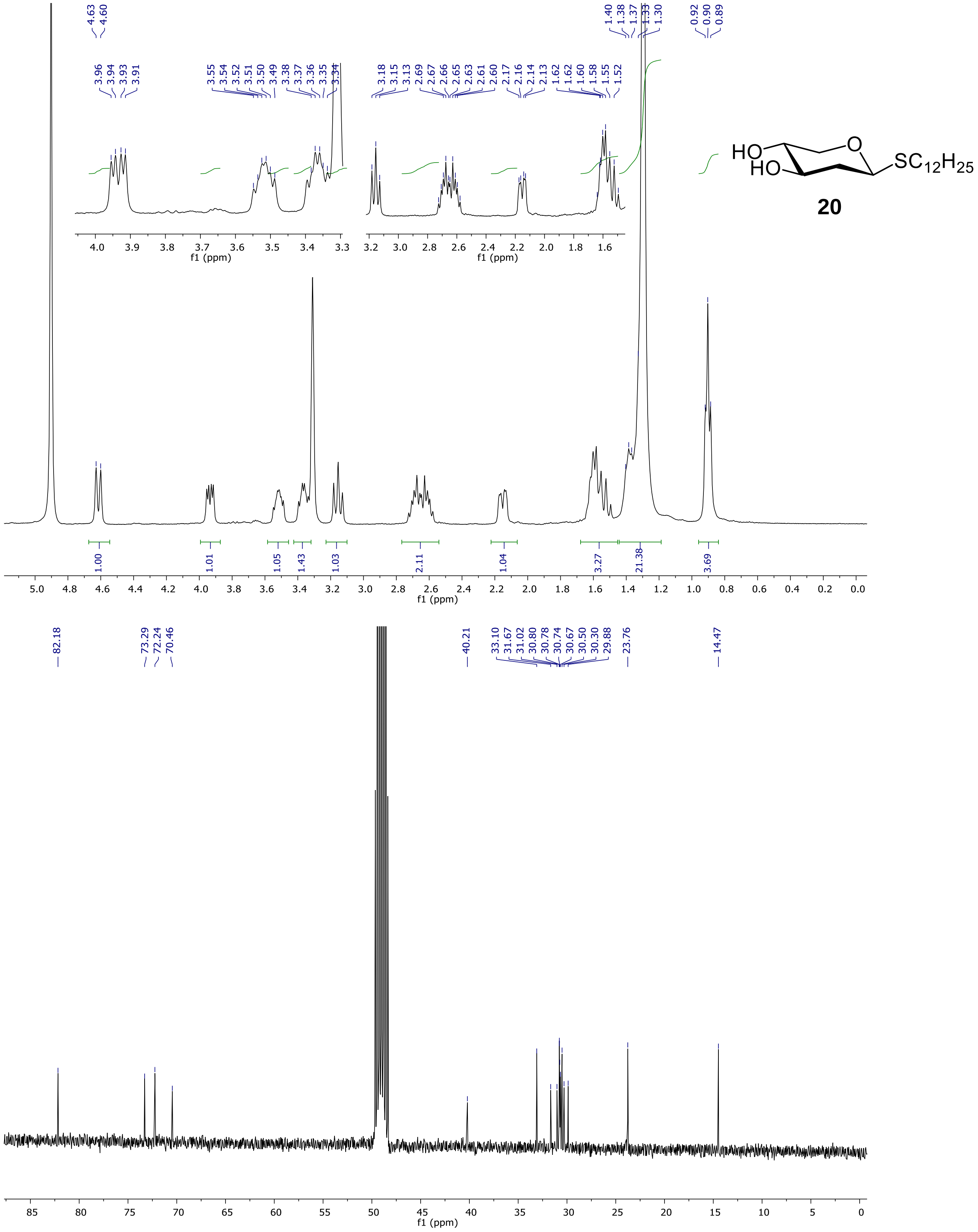

**Supplementary Fig. 38.** $^{1}$H NMR and $^{13}$C NMR spectra of compound **20**.

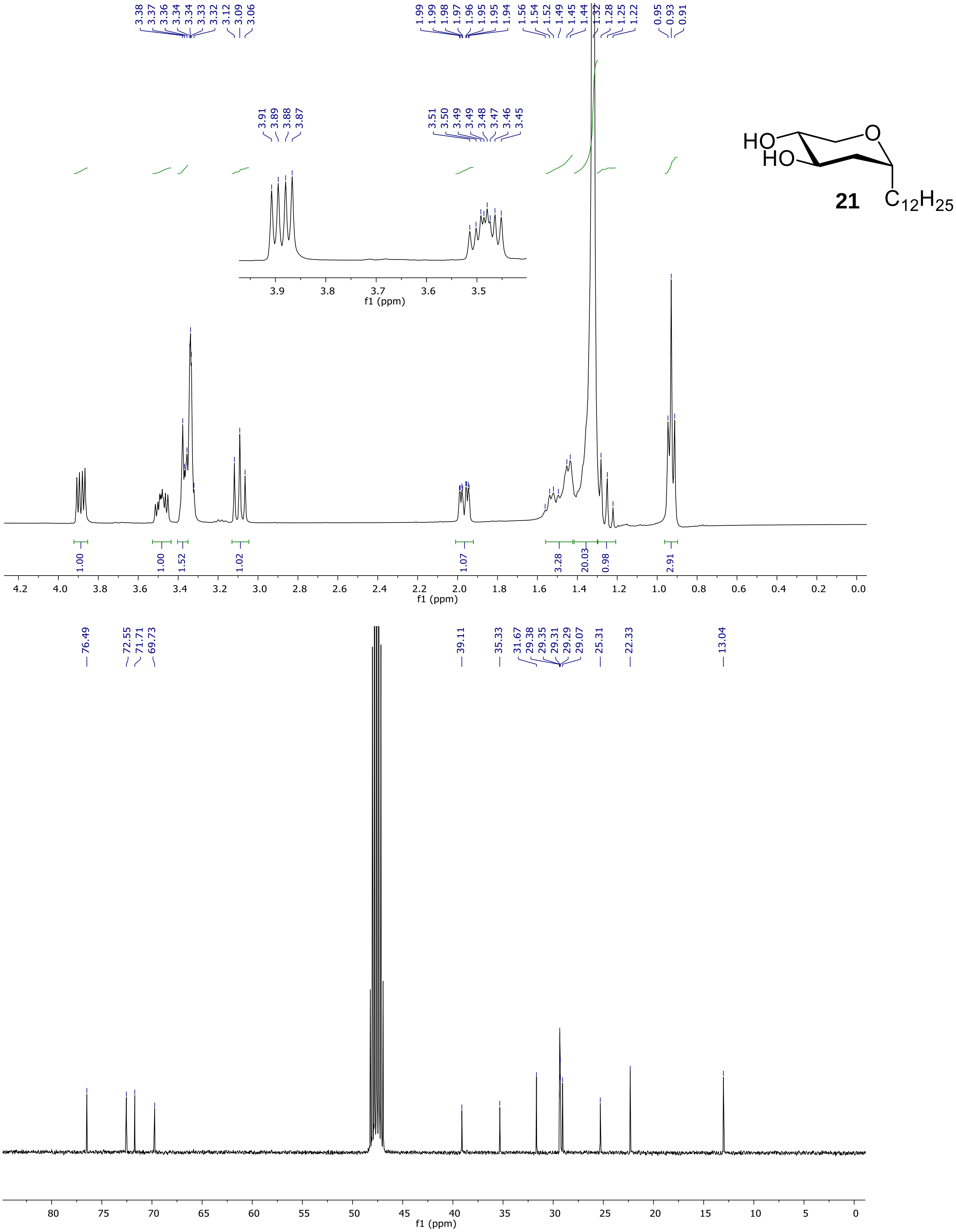


**Supplementary Fig. 39.** $^{1}$H NMR and $^{13}$C NMR spectra of compound **21**.

# 2 | Supplementary Tables

**Supplementary Table 1.** Relative Gibbs free energies between the two chair conformations of dodecyl 2-deoxy-α-L-*threo*-pentopyranoside (**7**) and dodecyl 2-deoxy-β-L-*threo*-pentopyranoside (**8**) obtained by DFT calculations

| **Solvent** | **Δ*G* ($^4C_1 \rightarrow {}^1C_4$) (kcal mol$^{-1}$)** | |
|---|---|---|
| | α-anomer **(7)** | β-anomer **(8)** |
| **Chloroform** | -4.2 | 1.0 |
| **Methanol** | -3.2 | 0.2 |

**Supplementary Table 2.** Cell death kinetics study at MIC and 2 times MIC concentration values of compound **1**

| **[CFU mL$^{-1}$]$^{ini}$** | **[1] (µg mL$^{-1}$)** | **T99.9 (min)** | **ΔLog CFU h$^{-1}$** | **ΔLog CFU/24h** | **MBC (µg mL$^{-1}$)** |
|---|---|---|---|---|---|
| $10^8$ | 16 | 40 | 4 | 4 | 32 |
| $10^8$ | 32 | 10 | 8 | 8 | 32 |
| $10^6$ | 16 | <10 | 6 | 6 | 16 |
| $10^6$ | 32 | <10 | 6 | 6 | 16 |

**Supplementary Table 3.** Susceptibility of *B. cereus* ATCC 14579 to deoxy *O*-/*S*-/*C*-glycosides

| **Compound Nr.** | **MIC ($\mu g\ mL^{-1}$)** | **MBC ($\mu g\ mL^{-1}$)** |
|---|---|---|
| **1** | 16 | 16 |
| **6** | 32 | 32 |
| **7** | 16 | 16 |
| **10** | 32 | 32 |
| **13** | 128 | 128 |
| **17** | 16 | 16 |
| **20** | 32 | 32 |

**Supplementary Table 4.** Susceptibility of resistant strains

| Antibiotic | MIC (μg mL$^{-1)}$ | Compound **1** | |
|---|---|---|---|
| | | MIC (μg mL$^{-1}$) | MBC (μg mL$^{-1}$) |
| **Erythromycin** | 20 | 16 | 16 |
| **Penicillin** | 20 | 16 | 16 |
| **Vancomycin** | 5 | 16 | 16 |
| **Ciprofloxacin** | 50 | 16 | 16 |
| **Tetracycline** | 50 | 16 | 16 |

**Supplementary Table 5.** Obtained mutants and their susceptibility to compound **1**

| Strain | Description | MIC (μg mL$^{-1}$) | MBC (μg mL$^{-1}$) |
|---|---|---|---|
| *B. cereus* ATCC 14579 | Control strain | 16 | 32 |
| *B. subtilis* 168 | Parental isogenic strain | 16 | 32 |
| *B. subtilis* ΔygaD | Putative ABC transporter (ATP-binding protein) | 16 | 16 |
| *B. subtilis* ΔyjdD | PTS mannose-specific enzyme IIBCA component | 16 | 16 |
| *B. subtilis* ΔyhcJ | Putative ABC transporter (binding lipoprotein) | 16 | 16 |
| *B. subtilis* ΔyhaQ | Na+-efflux ABC transporter (ATP-bindingprotein) | 16 | 16 |
| *B. subtilis* ΔykfD | Putative cell wall oligopeptide ABC transporter (ATP binding protein) | 16 | 16 |
| *B. subtilis* ΔykoD | Thiamine-related ABC transport system ATP-binding protein, thi-box regilon | 16 | 16 |
| *B. subtilis* ΔywhQ | ABC transport system ATP-binding protein, subtilosin production | 16 | 32 |
| *B. subtilis* ΔyknU | Putative ABC transporter (ATP-binding protein) | 16 | 32 |
| *B. subtilis* ΔykpA | Fructose-1-phosphate kinase (similar to ABC transport system ATP-binding protein) | 16 | 32 |
| *B. subtilis* ΔyheH | Multidrug ABC transporter (ATP-binding protein), involved in the signalling pathway that activates KinA during sporulation initiation | 16 | 32 |
| *B. subtilis* ΔyvcC | Multidrug ABC transporter | 16 | 32 |
| *B. subtilis* ΔywbA | Putative phosphotransferase system enzyme IIC permease component | 16 | 32 |
| *B. subtilis* ΔyxdM | ABC transporter (permease); efflux of cationicpeptides | 16 | 32 |
| *B. subtilis* ΔyybL | Putative integral inner membrane protein | 16 | 32 |
| *B. subtilis* ΔybbF | Putative sugar phosphotransferase enzyme II | 16 | 16 |

| | | | |
|---|---|---|---|
| *B. subtilis* ΔmtlA | Phosphotransferase system (PTS) mannitol-specific enzyme IICB component | 16 | 32 |
| *B. subtilis* ΔexpZ | ABC transport system ATP-binding protein, export of virginiamycin M and lincomycin | 16 | 32 |
| *B. subtilis* ΔydhO | PTS oligo-beta-mannoside-specific enzyme IIC component | 16 | 32 |
| *B. subtilis* ΔyfmR | Putative ABC protein involved in RecA-independent precise excision of transposons | 16 | 16 |
| *B. subtilis* ΔtreP | Phosphotransferase system (PTS) trehalose-specific enzyme IIBC component | 16 | 16 |
| *B. subtilis* ΔyfiB | ABC transporter ATP-binding protein | 32 | 32 |
| *B. subtilis* ΔyfiM | Putative ABC transporter (permease) | 16 | 16 |
| *B. subtilis* ΔyflF | PTS N-acetylglucosamine-specific enzyme IICB component | 16 | 32 |
| *B. subtilis* ΔyfiC | Putative ABC transporter (ATP-binding protein) | 16 | 16 |
| *B. subtilis* ΔyfiN | putative ABC transporter (permease) | 16 | 32 |
| *B. subtilis* ΔybdB | Exporter of cell killing factor, ABC transport system permease protein, the mutation exhibited highly oligosporogenous phenotypes | 16 | 32 |
| *B. subtilis* ΔybdA | Exporter of cell killing factor, ABC transport system ATP-binding protein, the mutation exhibited highly oligosporogenous phenotypes | 16 | 32 |

**Supplementary Table 6.** Activity of compound **1** on *E. coli*, *B. cereus*, *S. aureus*, and corresponding spheroplasts, protoplasts, expressed in MIC, compared with the activity of the standard antibiotic polymyxin B

| Strain | MIC (μg $mL^{-1}$) | Polymyxin B |
|---|---|---|
| *E. coli* K12 | 256 | 8 |
| *E. coli* K12 spheroplasts | 4 | >1 |
| *B. cereus* ATCC 14579 | 16 | 128 |
| *B. cereus* ATCC 14579 protoplasts | 16 | 16 |
| *S. aureus* ATCC 29213 | 256 | 128 |
| *S. aureus* ATCC 29213 protoplasts | 256 | >1 |

**Supplementary Table 7.** Susceptibility to compound **1** of diverse cellular forms of *B. cereus* ATCC 14579

| ***B. cereus* ATCC 14579** | **MBC (μg mL$^{-1}$)** |
|---|---|
| Vegetative cells | 32 |
| Spores | > 256 |
| Vegetative cells suspended in 1 mM PBS and 0.01 % Tween 20 | 16 |
| Spores suspended in 1 mM PBS and 0.01 % Tween 20 | >256 |
| Vegetative cells after heat treatment at 70 ºC for 30 min | 8 |
| Spores after heat treatment at 70 ºC for 30 min | 16 |
| Vegetative cells in AGFK buffer* at 37 ºC for 20 min | 8 |
| Spores after germination, achieved in AGFK buffer at 37 ºC for 20 min | 16 |

*AGFK buffer (30 mM L-asparagine, 5.6 mM D-glucose, 5.6 mM D-fructose, 20 mM KCl and 50 mM Tris-HCl, pH 8.4)

**Supplementary Table 8.** Antimicrobial susceptibility of *Bacillus cereus* ATCC 14579 to main antimicrobial classes and its mechanism of action

| Antimicrobial class[a] | Compound | Molecular Target and MoA[4] | MIC ($\mu g\ mL^{-1}$) | Reference |
|---|---|---|---|---|
| J01XB01 Polymyxins | Colistin | Membrane permeabilization and depolarization | >5000 | 5 |
| J01XB02 Polymyxins | Polymyxin B | Membrane permeabilization and depolarization | 781 | 5 |
| J01XX09 Lipopeptides | Daptomycin | Membrane permeabilization and depolarization | 3.125 | 6 |
| J01CA01 Penicillins | Ampicillin | Cell-wall biosynthesis – penicillin-binding protein (PBP) enzymes | 0.25 | 7 |
| J01XA01 Glycopeptides | Vancomycin | Cell-wall synthesis – lipid II binding | 0.18 | 8 |
| J01AA07 Tetracyclines | Tetracycline | Protein synthesis – binding to the 30S A-site | 2.5 | 9 |
| J01BA01 Amphenicols | Chloramphenicol | Protein synthesis – binding to the 23S rRNA of the 50S ribosomal subunit | 2.5 | 9 |
| J01FA01 Macrolides | Erythromycin | Protein synthesis – inhibition of 50S ribosomal subunit | 8 | 10 |
| J01GB03 Aminoglycosides | Gentamicin | Protein synthesis – binding to the 30S A-site | 4 | 10 |
| J01GB04 Aminoglycosides | Kanamycin | Protein synthesis – binding to the 30S A-site | 15 | 9 |
| J01MA02 Fluoroquinolones | Ciprofloxacin | DNA gyrase, topoisomerase IV | 0.32 | 9 |
| J01MA06 Fluoroquinolones | Norfloxacin | DNA gyrase, topoisomerase IV | 1.25 | 9 |
| J04AB02 Rifamycins | Rifampicin | RNA synthesis – inhibition of RNA polymerase | 0.5 | 11 |

[a]Antimicrobial classes and description according to the Anatomical Therapeutic Chemical (ATC) Classification System (adapted from WHO guidelines[12]).

# 3 | Supplementary Methods

## 3.1. Synthesis

Starting materials and reagents were purchased from Sigma–Aldrich, Fluka and Acros. The solvents were dried prior to use with 4 or 3 Å (methanol) molecular sieves. TLC was carried out on aluminium sheets (20 cm x 20 cm) coated with 0.2 mm silica gel 60 $F_{254}$ (Merck) and detection was accomplished by spraying the plates with a solution of $H_2SO_4$ in ethanol (10%) followed by heating at 120 °C. The compounds were purified by column chromatography (CC) using silica gel 60G (0.040–0.063 mm, Merck) or silica gel 60G (0.015–0.040 mm, Merck). Melting points were obtained with a SMP3 Melting Point Apparatus, Stuart Scientific, Bibby. Optical rotations were measured with a Perkin–Elmer 343 polarimeter at 20 ºC (589 nm, sodium D line). NMR spectra were recorded with a Bruker Avance 400 spectrometer at 298 K operating at 100.62 MHz for $^{13}C$ NMR and at 400.13 MHz for $^{1}H$ NMR. The solvents used were $CDCl_3$ with 0.03% TMS and $CD_3OD$ (Sigma–Aldrich). The chemical shifts are reported as $\delta$ (ppm) and the coupling constants ($J$) are given in Hz. NMR spectra for final compounds are provided in Supplementary Figs. 21-39. The assignment of the NMR signals was made with the help of NMR bidimensional experiments (COSY, HMQC, HMBC, NOESY). *C*-glycosides are named systematically and that is why glycone NMR data are given with primed locants, while for the *O*-/ and *S*-glycosides, primed locants are used for the dodecyl chain. The high resolution mass spectra of the new compounds were acquired on a Bruker Daltonics HR QqTOF Impact II mass spectrometer (Billerica, MA, USA). The nebulizer gas ($N_2$) pressure was set to 1.4 bar, and the drying gas ($N_2$) flow rate was set to 4.0 L $min^{-1}$ at a temperature of 200 °C. The capillary voltage was set to 4500 V and the charging voltage was set to 2000 V.

Starting materials and intermediate compounds that are not cited in the main text are here numbered in increasing order of appearance and preceded with "S". In one-pot procedures covering several steps, the intermediates were isolated for characterization purposes and data is given for the new compounds.

**Synthesis of dodecyl 2-deoxyglycosides and dodecyl 2-deoxy-1-thioglycosides.** The one-pot approach covering glycosylation and deacylation gave pure glycosides **1**, **5** and **17-20**. Isolation of protected compounds **S6-S13** was required to access the pure unprotected anomers, which purification via the one-pot procedure could not be achieved. Deprotection was accomplished by effective removal of the acetyl groups via Zemplén reaction, and the benzyl groups by catalytic hydrogenation, affording the target glycosides in almost quantitative yields (Supplementary Fig. 1). Glycal starting materials were synthesized as described below.

**General procedure for the synthesis of glycals S1, S2, S4 and S5.** To a solution of the free sugar (12.0 mmol) in pyridine (10 mL *per* g), acetic anhydride (2 equiv./OH) and DMAP (0.3 mmol) were added. Reaction mixture was stirred at room temperature for 2 h. Then, the solution was diluted with dichloromethane and washed three times with HCl 2M and then with water. The organic phase was dried

with anhydrous $MgSO_4$, filtered and concentrated under vacuum, to afford the peracetylated sugar as a colourless oil. The resulting product was then dissolved in glacial acetic acid (10 mL *per* g), followed by addition of methanol (18 mmol, 0.7 mL, 1.5 equiv.) and acetyl bromide (2.7 mL, 36 mmol, 3 equiv.). The reaction mixture was stirred, under nitrogen and light protection, at room temperature for 3 h. Dichloromethane (15 mL) was added and the solution was washed three times with saturated $NaHCO_3$, and with brine. The organic phase was dried with anhydrous $MgSO_4$, filtered and concentrated under vacuum, affording the corresponding glycosyl bromide. To a solution of glycosyl bromide in acetone (5 mL $mmol^{-1}$), sodium dihydrogen phosphate (0.26 mol, 41.21 g, 22 equiv.), and zinc (0.18 mol, 11.77g, 15 equiv.) were added and the mixture stirred vigorously, at room temperature. After 10 min, water (0.5 mL $mmol^{-1}$) was also added and the mixture kept under stirring for 1 h 30 min. The reaction mixture was extracted with EtOAc and washed twice with saturated $NaHCO_3$, and then with brine. The organic phase was dried with anhydrous $MgSO_4$, filtered and concentrated under vacuum. The resulting residue was purified by column chromatography with hex/EtOAc, affording the corresponding glycal.

**3,4-Di-*O*-acetyl-1,5-anhydro-2,6-dideoxy-L-*arabino*-hex-1-enitol (S1).** Obtained from L-rhamnose (8.0 g, 48.7 mmol) as a colourless oil (7.0 g, 67%); TLC (EtOAc/Pet. Et. 1:2 v/v): $R_f$ = 0.7; $^1$H NMR (400 MHz, $CDCl_3$) δ 6.38 (br d, 1H, $J_{1,2}$ = 6.1 Hz, H-1), 5.35 (m, 1H, H-3), 5.03 (dd, 1H, $J_{3,4}$=6.4 Hz, $J_{4,5}$=7.0 Hz, H-4), 4.79 (dd, 1H, $J_{2,3}$= 3.0 Hz, H-2), 4.11 (qd, 1H, $J_{4,5}$= $J_{5,6}$=7.0 Hz, H-5), 2.09 (s,, 3H, -OAc), 2.05 (s, 3H, -OAc), 1.31 (d, 3H, H-6). $^{13}$C NMR (100.6 MHz, $CDCl_3$) δ 170.7, 169.9 (C=O, OAc), 145.0 (C-1), 98.80 (C-2), 72.5 (C-5), 71.8 (C-4), 68.3 (C-3), 21.1 (-$CH_3$, OAc), 20.7 (-$CH_3$, OAc), 17.1 (C-6). Spectroscopic data is in full agreement with the literature[13].

**3,4-Di-*O*-acetyl-1,5-anhydro-2,6-dideoxy-L-*lyxo*-hex-1-enitol (S2).** Obtained from L-fucose (1.7 g, 10.4 mmol) as a syrup (0.794 g, 36%); TLC (EtOAc/hex, 1:5 v/v): $R_f$ = 0.66; $[\alpha]_D$ = +8 º (c1.0, $CHCl_3$); $^1$H NMR (400 MHz, $CDCl_3$) δ 6.47 (br d, 1H, $J_{1,2}$ = 6.3 Hz, H-1), 5.58 (m, 1H, H-3), 5.29 (dd, 1H, $J_{3,4}$=1.1 Hz, $J_{4,5}$ = 3.1 Hz, H-4), 4.64 (dd, 1H, $J_{2,3}$=1.3 Hz, H-2), 4.06 (qd, 1H, $J_{5,6}$=6.5 Hz, H-5), 2.16 (s, 3H, OAc), 2.02 (s, 3H, OAc), 1.28 (d, 3H, H-6); $^{13}$C NMR (100.6 MHz, $CDCl_3$) δ 170.6 (C=O, OAc), 170.3 (C=O, OAc), 146.0 (C-1), 98.1 (C-2), 71.4 (C-5), 66.1 (C-4), 64.9 (C-3), 21.1 (-$CH_3$, OAc), 20.7 (-$CH_3$, OAc), 20.6 (-$CH_3$, OAc), 16.4 (C-6). Spectroscopic data is in full agreement with the literature[14].

**3,4-Di-*O*-acetyl-1,5-anhydro-2-deoxy-L-*threo*-pent-1-enitol (S4)**. Obtained from L-xylose (0.9 g, 6.0 mmol) as a syrup (0.80 g, 66%); TLC (Pet. Et./EtOAc, 3:1 v/v): $R_f$ = 0.76; $^1$H NMR (400 MHz, $CDCl_3$) δ 6.61 (d, 1H, $J_{1,2}$ = 5.6 Hz, H-1), 5.00-4.95 (m, 3H, H-2, H-3, H-4), 4.20 (br d, 1H, $J_{5a,5b}$ = 12.3 Hz, H-5a), 3.98 (br d, H-5b), 2.10 (s, 3H, OAc), 2.07 (s, 3H, OAc); $^{13}$C NMR (100.6 MHz, $CDCl_3$) δ 169.9 (C=O, OAc), 169.8 (C=O, OAc), 148.0 (C-1), 97.4 (C-2), 67.2 (C-4), 63.6 (C-5), 63.4 (C-3), 21.1 ($CH_3$, OAc), 21.0 (-$CH_3$, OAc).

**3,4-Di-*O*-acetyl-1,5-anhydro-2-deoxy-D-*threo*-pent-1-enitol (S5).** Obtained from D-xylose (10.0 g, 66.61 mmol) as a syrup (8.658 g, 65%); TLC (EtOAc/Petrol. Ether, 1:6 v/v): $R_f$ = 0.34; NMR data was in full agreement with that described above for its enantiomer (compound **S4**), and with the literature[13].

**3,4-Di-*O*-benzyl-1,5-anhydro-2,6-dideoxy-L-*arabino*-hex-1-enitol (S3).** To a solution of glycal **S1** (2.197g, 10.26 mmol) in THF (70 mL), powdered NaOH (4.24 g, 102.60 mmol, 10 equiv.) and TBAI

(1.895g, 5.13 mmol, 0.5 equiv.) were added. After 10 min stirring at room temperature, benzyl bromide (3.7 mL, 30.78 mmol, 6 equiv.) was added and the mixture was stirred for 3 h. Reaction mixture was then poured in water (150 mL) and extracted with dichloromethane (3x100 mL). The organic phase was washed with water, dried with anhydrous $MgSO_4$, filtered and concentrated under vacuum. The resulting residue was purified by column chromatography with hex/EtOAc 60:1, affording the title compound as a colourless oil (2.558 g, 80%); TLC (hex/EtOAc, 6:1 v/v): $R_f$ = 0.71; $[\alpha]_D$ = +16 º (c1.0, $CHCl_3$); $^1$H NMR (400 MHz, $CDCl_3$) δ 7.38-7.26 (m, 10H, Ph) 6.36 (d, 1H, $J_{1,2}$ = 6.0 Hz, H-1), 4.90-4.84 (m, 2H, H-2, part A of AB system, $CH_2$Ph), 4.71-4.64 (m, 2H, $CH_2$Ph), 4.57 (part B of AB system, 1H, C<u>H</u>$_2$Ph), 4.21 (br d, 1H, $J_{2,3}$ = 6.2 Hz, H-3), 3.95 (qd, 1H, $J_{5,6}$=6.7 Hz, H-5), 3.48 (dd, 1H, $J_{3,4}$= 8.7 Hz, H-4), 1.36 (d, 3H, H-6); $^{13}$C NMR (100.6 MHz, $CDCl_3$) δ 144.7 (C-1), 138.4, 138.2, 128.4, 127.9, 127.7, 127.6 (Ph, OBn), 100.1 (C-2), 79.5 (C-4), 76.4 (C-3), 74.0 (C-5) 73.9 (<u>C</u>H$_2$, -OBn), 70.5 (<u>C</u>H$_2$, -OBn), 17.5 (C-6). Spectroscopic data is in full agreement with the literature[15].

**General one-pot procedure for the synthesis of dodecyl 2-deoxyglycosides 1, 5, 17-20.** Glycal (**S1** or **S5**, 5.0 mmol) was dissolved in dichloromethane (10 mL *per* g), followed by the addition of TPHB (0.1 equiv or 0.3 equiv. for reaction with dodecan-1-ol or dodecan-1-thiol, respectively), and dodecan-1-ol (1.5 equiv. for **S1** and 1.2 equiv. for **S5**) or dodecan-1-thiol (2 equiv). The reaction was stirred at room temperature for 1 h (**1**, **5**), 2 h (**17, 18**) or overnight (**19**, **20**). Reaction mixture was either directly neutralized with a free base resin (e.g. IRA-400 for **1**, **5** or Amberlyst A21 for **17, 18**) and concentrated under vacuum, or diluted with dichloromethane, washed with saturated $NaHCO_3$, then with water, and the organic phase was dried with magnesium sulfate and concentrated in vacuum (**19**, **20**). In all cases, the resulting residue was dissolved in methanol (0.1mL *per* mg) and a freshly prepared solution of 1% NaOMe in MeOH (0.1 mL *per* 100 mg residue) was added. The mixture was stirred at room temperature for 1 h. Neutralization with Amberlite (IR-120) was followed by filtration and evaporation of the solvent. The residue was purified by column chromatography (CC).

**Dodecyl 2,6-dideoxy-α-L-*arabino*-hexopyranoside (1).** Obtained from glycal **S1** in 67% isolated yield after CC eluted with EtOAc. Physical and spectroscopic data in full agreement with the literature[1].

**Dodecyl 2,6-dideoxy-β-L-*arabino*-hexopyranoside (5).** Obtained from glycal **S1** in 14% isolated yield after CC eluted with EtOAc. Physical and spectroscopic data in full agreement with the literature[1].

**Dodecyl 2-deoxy-α-D-*threo*-pentopyranoside (17).** Obtained from glycal **S5** in 33% isolated yield after CC eluted with hex/EtOAc 5:1; mp: 53.6-54.4 ºC; $[\alpha]_D$ = +81º (*c* 1, MeOH); TLC (hex/EtOAc, 2:1 v/v): $R_f$ = 0.24; $^1$H NMR (400 MHz, $CDCl_3$): δ 4.84 (dd, 1H, $J_{1,2eq}$ = 1.7 Hz, $J_{1,2ax}$= 3.3 Hz, H-1), 3.96-3.88 (m, 1H, H-3), 3.68 (dd, 1H, $J_{5eq,5ax}$= 9.9 Hz, $J_{4,5eq}$= 4.1 Hz, H-5eq), 3.66-3.60 (m, 1H, H-1'a), 3.59-3.46 (m, 2H, H-4, H-5ax), 3.37-3.31 (m, 1H, H-1'b), 2.44 (s, 1H, OH-4), 2.35 (s, 1H, OH-3), 2.11 (ddd, 1H, $J_{2eq,2ax}$= 12.8 Hz, $J_{2eq,3}$=4.1 Hz, $J_{1,2eq}$=1.7 Hz, H-2eq), 1.64 (ddd, 1H, $J_{2ax,2eq}$=12.8 Hz, $J_{2ax,3}$=10.9 Hz, $J_{1,2ax}$=3.3 Hz, H-2ax),), 1.60-1.53 (m, 2H, H-2'), 1.38-1.19 (m, 18H, H-3' - H-11'), 0.86 (t, 3H, $J_{11',12'}$= 6.7 Hz, H-12'); $^{13}$C NMR (100.6 MHz, $CDCl_3$): δ 97.6 (C-1), 72.1 (C-4), 69.7 (C-3), 67.6 (C-1'), 62.1 (C-5), 37.3 (C-2), 31.9, 29.7, 29.7, 29.6, 29.6, 29.6, 29.5, 29.4, 26.2, 22.7 (C-2' – C-11'), 14.3 (C-12'); HRMS (*m/z*): Calcd for $[M+Na]^+$ 325.2349; Found: 325.2338 (error 3.5 ppm).

**Dodecyl 2-deoxy-β-D-*threo*-pentopyranoside** (**18**). Obtained from glycal **S5** in 18% isolated yield after CC eluted with hex/EtOAc 5:1; mp: 68.1-68.7 ºC; TLC (hex/EtOAc, 2:1 v/v): $R_f$ = 0.19; $[\alpha]_D$ = −72 (*c* 1, MeOH); $^1$H NMR (400 MHz, $CDCl_3$): δ 4.78 (t, 1H, $J_{1,2ax}$ =$J_{1,2eq}$= 3,5 Hz, H-1), 4.14 (dd, 1H, $J_{5ax,5eq}$= 12.5 Hz; $J_{4,5eq}$= 1.7 Hz, H-5eq), δ 3.78-3.71 (m, 2H, H-3, H-1'a), 3.64 (br s, 1H, H-4), 3.48-3.37 (m, 3H, H-5ax, H-1'b, OH), 2.25-2,15 (m,2H, H-2eq, OH), 1.78 (dt, 1H, $J_{2ax,2eq}$=14.2 Hz, $J_{1,2ax}$=$J_{2ax,3}$=3.5 Hz, H-2ax), 1.64-1.56 (m, 2H, H-2'),1.39-1.19 (m, 18H, H-3' - H-11'), 0.89 (t, 3H, $J_{11',12'}$ = 6.7 Hz, H-12'); $^{13}$C NMR (100.6 MHz, $CDCl_3$): δ 98.1 (C-1), 69.3 (C-4), 68.6 (C-1'), 67.9 (C-3), 60.8 (C-5), 32.3 (C-2), 31.9, 29.7, 29.6, 29.6, 29.6, 29.5, 29.4, 29.4, 26.2, 22.7 (C-2' to C-11'), 14.3 (C-12'); HRMS (*m/z*): Calcd for $[M+Na]^+$ 325.2349; Found: 325.2336 (error 4.1 ppm).

**Dodecyl 2-deoxy-1-thio-α-D-*threo*-pentopyranoside** (**19**). Obtained from **S5** in 15% isolated yield after CC eluted with toluene/EtOAc 3:2; mp: 75.9-78.9 ºC; TLC (EtOAc/tol., 3:2 v/v): $R_f$ = 0.38; $[\alpha]_D$ = +130º (*c*1.0, $CHCl_3$); $^1$H NMR (400 MHz, MeOD) δ 5.22 (t, 1H, $J_{1,2ax}$=$J_{1,2eq}$=4.0 Hz, H-1), 3.80 (dd, 1H, $J_{4,5ax}$=8.9 Hz, $J_{5ax,5eq}$=11.5 Hz, H-5ax), 3.73 (ddd, 1H, $J_{3,4}$=8.0 Hz, $J_{2ax,3}$=9.0 Hz, $J_{3, 2eq}$ = 3.9 Hz, H-3), 3.65 (dd, $J_{4,5eq}$= 4.7 Hz, H-5eq), 3.37 (ddd, H-4), 2.60 (dt, 1H, $J_{1'a,1'b}$=12.5 Hz, $J_{1'a,2'}$= 7.0 Hz, H-1'a), 2.54 (dt, 1H, $J_{1'b,2'}$= 7.0 Hz, H-1'b), 2.10 (dt, 1H, $J_{1,2eq}$=3.9 Hz $J_{2ax,2eq}$=13.5 Hz, H-2eq), 1.88 (ddd, $J_{1,2ax}$=4.5 Hz, 1H, H-2ax),1.66-1.55 (m, 2H, H-2'), 1.44-1.22 (m, 18H, H-3' - H-11'), 0.90 (t, 3H, $J_{11,12}$= 7.1 Hz, H-12'); $^{13}$C NMR (100.6 MHz, MeOD) δ 81.8 (C-1), 72.2 (C-4), 70.1 (C-3), 64.7 (C-5), 38.5 (C-2), 33.1 (C-1'), 31.7, 30.8, 30.7, 30.5, 30.3, 29.9 (C-2' a C-10'), 23.8 (C-11'), 14.5 (C-12'); HRMS (*m/z*): Calcd for $[M+Na]^+$ 341.2121; Found: 341.2112.

**Dodecyl 2-deoxy-1-thio-β-D-*threo*-pentopyranoside** (**20**)**.** Obtained from **S5** in 31% isolated yield after CC eluted with toluene/EtOAc 3:2; mp: 84.9-86.5 ºC; TLC (EtOAc): $R_f$ = 0.26; $[\alpha]_D$ =−54º (c1.0, $CHCl_3$); $^1$H NMR (400 MHz, MeOD) δ 4.61 (dd, 1H, $J_{1,2ax}$=11.3 Hz, $J_{1,2eq}$=1.8 Hz, H-1), 3.93 (dd, 1H, $J_{4,5eq}$=5.0 Hz, $J_{5ax,5eq}$=11.4 Hz, H-5eq), 3.52 (br ddd, 1H, $J_{3,4}$=10.0 Hz, $J_{2ax,3}$=5 Hz, $J_{2eq,3}$=1.8 Hz, H-3), 3.38 (ddd, 1H, $J_{4,5ax}$= 11.4 Hz, H-4), 3.18 (t, 1H, $J_{4,5ax}$=$J_{5ax,5eq}$=11.4 Hz, H-5ax), 2.69 (dt, 1H, $J_{1'a,1'b}$=13.0 Hz, $J_{1'a,2'}$= 7.2 Hz, H-1'a), 2.61 (dt, 1H, $J_{1'b,2'}$= 7.4 Hz, H-1'b), 2.15 (ddd, 1H, $J_{1,2eq}$=1.9 Hz, $J_{2eq,3}$=1.8 Hz, $J_{2ax,2eq}$=12.8 Hz, H-2eq), 1.64-1.49 (ddd, 1H, $J_{1,2ax}$=4.5 Hz, $J_{2ax,3}$=5 Hz, H-2ax),1.66-1.55 (m, 2H, H-2'), 1.43-1.22 (m, 18H, H-3' - H-11'), 0.90 (t, 3H, $J_{11,12}$= 6.5 Hz, H-12'); $^{13}$C NMR (100.6 MHz, MeOD) δ 82.2 (C-1), 73.3 (C-3), 72.3 (C-4), 70.5 (C-5), 40.2 (C-2), 33.1 (C-1'), 31.7, 31.0 30.8, 30.7, 30.5, 30.3, 29.9 (C-2' a C-10'), 23.8 (C-11'), 14.5 (C-12'). HRMS (*m/z*): Calcd for $[M+Na]^+$ 341.2121; Found: 341.2113.

**Dodecyl 2,3-dideoxy-1-thio-D-*glycero*-pent-2-enopyranoside (S15α,β)**. Obtained from glycal **S5** as an anomeric mixture (1.9:1 α/β) in 10.6% isolated yield, after CC eluted with toluene; TLC (toluene/EtOAc, 3:2 v/v): $R_f$ = 0.88; $^1$H NMR (400 MHz, acetone-$d_6$) δ 5.95 (br dd, $J_{2,3}$=10 Hz, H-3α), 5.88 (dd, $J_{1,2}$=3 Hz, $J_{2,3}$=10 Hz, H-2α), 5.78-5.65 (m, H-2β , H-3β), 4.48 (br s, H-1α), 4.44 (d, $J_{1,2}$=7.53 Hz, H-1β) , 4.18-4.17 (dd $J_{4,5eq}$=2.4 Hz, $J_{5ax,5eq}$=12 Hz, H-5eq α, H-4β), 4.03 (br s, OHα), 3.80 (br s, H-4α), 3.64 (d, H-5ax α), 3.55-3-39 (m, H-5β), 2.72-2.55 (m, H-1'α,β), 1.68-1.53 (m, H-2'α,β), 1.44-1.35 (m, H-3'α,β), 1.34-1.20 (m, H-4'α,β - H-11'α,β), 0.88 (t, $J_{11',12'}$=7 Hz, H-12'αβ); $^{13}$C NMR (100.6 MHz, acetone-$d_6$) δ 133.1 (C-3β), 130.0 (C-2β), 129.9 (C-2α), 129.1 (C-3α) 80.2 (C-1α), 72.9 (C-4β), 67.6 (C-5β), 65.5 (C-5α), 62.1 (C-4α), 51.3 (C-1β), 32.7 (C-1'α,β), 31.9 (C-2'α), 31.6 (C-2'β), 30.9, 30.4, 30.3,

30.2, 30.1, 29.8, 29.6, 29.5 (C-3' - C10', partially under acetone-$d_6$ signal), 32.4 (C-11'α,β), 14.4 (C-12'αβ); HRMS (*m/z*): Calcd for $[M+H]^+$ 301.2196; Found: 301.2196 (error 0.0 ppm).

**Glycosylation procedure for the synthesis of dodecyl glycosides 2, 4/4b, 7 and 8.** Glycal **(S2, S3** or S**4,** 5.0 mmol**)** was dissolved in dichloromethane (10 mL *per* g), followed by the addition of TPHB (0.1 equiv or 0.3 equiv. for reaction with dodecan-1-ol or dodecan-1-thiol, respectively), and dodecan-1-ol or dodecan-1-thiol (2 equiv). The reaction was stirred at room temperature for 3 h (**S8, S10**) or overnight (**S9**, **S13** and **S7**, **S12**)**.** Reaction mixture was diluted with dichloromethane, washed with saturated $NaHCO_3$ and water, and the organic phase was dried with magnesium sulfate and concentrated in vacuum. The residue was purified by column chromatography.

**Dodecyl 2,6-dideoxy-3,4-di-*O*-acetyl-α-L-*lyxo*-hexopyranoside (S8).** Obtained from glycal **S2** in 71% isolated yield, after CC eluted with hex/EtOAc 17:1; mp: 40.9-41.7 ºC; TLC (hex/EtOAc, 5:1 v/v): $R_f$ = 0.43; $[\alpha]_D$ = -104º (*c*1.0, $CHCl_3$); $^1H$ NMR (400 MHz, $CDCl_3$) δ 5.26 (br dd, 1H, $J_{2ax,3}$= 12.8 Hz, $J_{2eq,3}$=5.4 Hz, H-3), 5.16 (br s, 1H, H-4), 4.92 (d, 1H, $J_{1,2ax}$=2.6 Hz, H-1), 4.03 (q, 1H, $J_{5,6}$=6.5 Hz, H-5), 3.60 (td, 1H, H-1'a), 3.34 (td, 1H, H-1'b), 2.13 (s, 3H, -OAc), 2.02 (td, 1H, $J_{2ax,2eq}$ = $J_{2ax,3}$=12.8 Hz, $J_{1,2ax}$=2.6 Hz, H-2ax), 1.96 (s, 3H, -OAc), 1.82 (dd, 1H, H-2eq), 1.53 (m, 2H, H-2'), 1.34-1.19 (m, 18H, H-3' - H-11'), 1.11 (d, 3H, H-6), 0.85 (t, 3H, $J_{11',12'}$= 6.6 Hz, H-12'); $^{13}C$ NMR (100.6 MHz, $CDCl_3$) δ 170.8, 170.2 (C=O, OAc), 97.3 (C-1), 69.9 (C-4), 67.5 (C-1'), 66.9 (C-3), 64.4 (C-5), 31.9 (C-2), 30.0, 29.7, 29.6, 29.5, 29.4, 29.3, 29.2, 22.7 (C-2' - C-11'), 21.0, 20.8 ($CH_3$, OAc), 16.5 (C-6), 14.3 (C-12'); HRMS (*m/z*): Calcd for $[M+H]^+$ 401.2898; Found: 401.2888 (error 2.4 ppm).

**Dodecyl 2,6-dideoxy-3,4-di-*O*-acetyl-β-L-*lyxo*-hexopyranoside (S10)**. Obtained from glycal **S2** in 14% isolated yield, after CC eluted with hex/EtOAc 17:1 as a colourless oil; TLC (hex/EtOAc, 5:1 v/v): $R_f$ =0.43; $[\alpha]_D$ = +8º (*c*1.0, $CHCl_3$) $^1H$ NMR (400 MHz, $CDCl_3$) δ 5.09 (br d, 1H, $J_{3,4}$ = 2.5 Hz, H-4), 4.97 (ddd, 1H, $J_{2ax,3}$= 13.2 Hz, $J_{2eq,3}$=5.6, H-3), 4.50 (dd, 1H, $J_{1,2ax}$=9.3 Hz, $J_{1,2eq}$=2.2 Hz, H-1), 3.91 (td, 1H, H-1'a), 3.65 (q, 1H, $J_{5,6}$=6.5, H-5), 3.44 (td, 1H, H-1'b), 2.15 (s, 3H, -OAc), 1.99 (s, 3H, -OAc), 1.97-1.85 (m, H-2ax and H2eq), 1.64-1.50 (m, 2H, H-2'), 1.35-1.17 (m, 19H, H-3' - H-11', H-6), 0.87 (t, 3H, $J_{11,12}$= 6.5 Hz, H-12'); $^{13}C$ NMR (100.6 MHz, $CDCl_3$) δ 170.9, 170.2 (C=O, OAc), 99.8 (C-1), 69.8 (C-1'), 69.2, 69.1 (C-3 and C-5), 68.7 (C-3), 31.9 (C-2), 31.8, 29.7, 29.6, 29.6, 29.5, 29.4, 29.3, 26.0, 22.7 (C-2' - C-11'), 20.9, 20.8 ($CH_3$, -OAc), 16.5 (C-6), 14.1 (C-12'); HRMS (*m/z*): Calcd for $[M+Na]^+$ 423.2717; Found: 423.2709 (error 1.8 ppm).

**Dodecyl 3,4-di-*O*-benzyl-2,6-dideoxy-1-thio-α-L-*arabino*-hexopyranoside (S7)** and **dodecyl 3,4-di-*O*-benzyl-2,6-dideoxy-1-thio-β-L-*arabino*-hexopyranoside (S12).** Obtained from glycal **S3** in 45% yield as an anomeric mixture (α/β ratio 3:1) after CC eluted with hex/EtOAc 30:1.$^1H$ NMR (400 MHz, $CDCl_3$) δ 7.34-7-24 (Ph, -OBn) 5.00-5.28 (m, $J_{1,2ax}$=6.2 Hz, H-1α), 5.00-4.94 (m, C<u>H</u>$_2$Ph, α,β), 4.70-4.57 (m, C<u>H</u>$_2$Ph, α, β), 4.46 (t, $J_{1,2}$=11 Hz, H-1β), 4.13-4.04 (m, H-5α, H-5β), 3.87 (ddd, , $J_{2e,3}$=4.2 Hz, $J_{3,4}$=8.9 Hz, $J_{2a,3}$=7.1 Hz, H-3α), 3.69-3.61 (m, H-3β), 3.15-3.07 (m, H-4α,β), 2.71-2.61 (m, H1'β), 2.59-2.43 (m, H1'α), 2.35 (dd, $J_{2ax,2eq}$=13.2 Hz, H-$2_{eq}$β), 2.28 (dd, $J_{2ax,2eq}$=13.3 Hz, H-$2_{eq}$α), 1.99 (m, H-$2_{ax}$α, H-$2_{ax}$β), 1.61-1.51 (m, H-2'α,β), 1.37-1.18 (m, H-3'-H11' α,β, H-6α,β), 0.87 (t, $J_{11',12'}$=6.5 Hz, H-12'α, H-12'β); $^{13}C$ NMR (100.6 MHz, $CDCl_3$) δ 128.5, 128.4, 128.1, 128.0, 127.7, 127.6 ($CH_2$-<u>Ph</u>), 84.6 (C-1α), 83.6

(C-1β), 80.5 (-$\underline{C}H_2$Ph β), 80.4 (-$\underline{C}H_2$Ph α), 79.6 (-$\underline{C}H_2$Ph β), 77.9 (-$\underline{C}H_2$Ph α), 75.6 (C-4β), 75.4 (C-3β), 75.2 (C-4 α), 71.8 (C-3α), 67.5 (C-5α), 60.4 (C5-β), 37.3 (C-2'β), 36.3 (C-2'α), 31.90 (C-1'α, C-1'β), 31.10, 30.8, 29.9, 29.7, 29.7,29.7, 29.6, 29.6, 29.4, 29.2, 28.9, 22.7 (C-2' - C-11' α, β), 18.4 (C-6β), 18.1 (C-6α), 14.2 (C-12'β), 14.1 (C-12'α). Further purification by column chromatography allowed the isolation of a fraction of the alfa anomer **S7**, which was submitted to hydrogenation to remove benzyl protecting groups to afford compound **2**.

**Dodecyl 3,4-di-*O*-acetyl-2-deoxy-α-L-*threo*-pentopyranoside (S9).** Obtained from glycal **S4** in 23% isolated yield, after CC eluted with hex/EtOAc 95:5 as a syrup; TLC (hex/EtOAc, 6:1 v/v): $R_f$ = 0.55; $[\alpha]_D$ = −58º (c 1.0, $CHCl_3$); $^1$H NMR (400 MHz, $CDCl_3$) δ 5.27 (ddd, 1H, $J_{3,4}$ = 8.5 Hz, $J_{2ax,3}$ = 10.1 Hz, $J_{2eq,3}$ = 4.8 Hz, H-3), 4.86 (ddd, 1H, $J_{3,4}$ = 8.5 Hz, $J_{4,5ax}$ = 9.2 Hz, $J_{4,5eq}$ = 5.0 Hz, H-4), 4.82 (br t, 1H, $J_{1,2ax}$ = 3.2 Hz, $J_{1,2eq}$ = 2.8 Hz, H-1), 3.77 (dd, 1H, $J_{5ax,5eq}$ = 11.1 Hz, $J_{4,5eq}$ = 5.0 Hz, H-5eq), 3.69-3.62 (m, 2H, H-5ax, H-1'a), 3.35 (dt, 1H, $J_{1'a,1'b}$ = 9.3 Hz, $J_{1'b,2'}$ = 6.7 Hz, H-1'b), 2.18 (ddd, 1H, $J_{2ax,2eq}$ = 13.0 Hz, $J_{2eq,3}$ = 4.8 Hz, $J_{1,2eq}$ = 2.8 Hz, H-2eq), 2.05 (s, 3H, -OAc), 2.04 (s, 3H, -OAc), 1.76 (ddd, 1H, $J_{2ax,2eq}$ = 13.0 Hz, $J_{2ax,3}$ = 10.1 Hz, H-2ax), 1.61-1.54 (m, 2H, H-2'), 1.36-1.23 (m, 18H, H-3' to H-11'), 0.88 (t, 3H, $J_{11',12'}$ = 6.8 Hz, H-12'); $^{13}$C NMR (100.6 MHz, $CDCl_3$) δ 170.2 (C=O, OAc), 170.1 (C=O, OAc), 97.0 (C-1), 69.6 (C-4), 68.6 (C-3), 68.0 (C-1'), 59.8 (C-5), 34.6 (C-2), 31.9, 29.7, 29.6, 29.6, 29.6, 29.5, 29.5, 29.4, 26.2, 22.7 (C-2’ to C-11'), 21.1 ($CH_3$, -OAc), 20.9 ($CH_3$, -OAc), 14.1 (C-12'); HRMS (*m/z*): Calcd for $[M+Na]^+$ 409.2561; Found: 409.2568 (error 1.7 ppm).

**Dodecyl 3,4-di-*O*-acetyl-2-deoxy-β-L-*threo*-pentopyranoside (S13).** Obtained from glycal **S4** in 10% isolated yield, after CC eluted with hex/EtOAc 95:5 as a syrup; TLC (hex/EtOAc, 6:1 v/v): $R_f$ = 0.48; $[\alpha]_D$ = +52º (c 1.0, $CHCl_3$); $^1$H NMR (400 MHz, $CDCl_3$) δ 4.94 (ddd, 1H, $J_{3,4}$ = 6.5 Hz, $J_{2ax,3}$ = 8.7 Hz, $J_{2eq,3}$ = 4.6 Hz, H-3), 4.84 (ddd, 1H, $J_{3,4}$ = 6.5 Hz, $J_{4,5ax}$ = 7.3 Hz, $J_{4,5eq}$ = 3.9 Hz, H-4), 4.62 (dd, 1H, $J_{1,2ax}$ = 6.3 Hz, $J_{1,2eq}$ = 2.7 Hz, H-1), 4.12 (dd, 1H, $J_{5ax,5eq}$ = 12.1 Hz, $J_{4,5eq}$ = 3.9 Hz, H-5eq), 3.76 (dt, 1H, $J_{1'a,1'b}$ = 9.2 Hz, $J_{1'a,2'}$ = 6.8 Hz, H-1'a), 3.43-3.35 (m, 2H, H-5ax, H-1'b), 2.23 (ddd, 1H, $J_{2ax,2eq}$ = 13.3 Hz, $J_{2eq,3}$ = 4.6 Hz, $J_{1,2eq}$ = 2.7 Hz, H-2eq), 2.07 (s, 3H, -OAc), 2.06 (s, 3H, -OAc), 1.79 (ddd, 1H, $J_{2ax,2eq}$ = 13.3 Hz, $J_{2ax,3}$ = 8.7 Hz, $J_{1,2ax}$ = 6.3 Hz, H-2ax), 1.61-1.53 (m, 2H, H-2'), 1.35-1.21 (m, 18H, H-3' - H-11', 0.88 (t, 3H, $J_{11',12'}$= 6.5 Hz, H-12'); $^{13}$C NMR (100.6 MHz, $CDCl_3$) δ 170.2 (C=O, OAc), 170.1 (C=O, OAc), 98.2 (C-1), 69.1 (C-4), 68.9 (C-1'), 68.6 (C-3), 60.9 (C-5), 33.3 (C-2), 31.9, 29.7, 29.7, 29.6, 29.6, 29.6, 29.4, 29.4, 26.1, 22.7 (C-2’ - C-11'), 21.1 ($CH_3$, -OAc), 21.0 ($CH_3$, -OAc), 14.1 (C-12'); HRMS (*m/z*): Calcd for $[M+Na]^+$ 409.2561; Found: 409.2565 (error 1.0 ppm).

**Dodecyl 4-*O*-acetyl-2,3-dideoxy-α-L-*glycero*-pent-2-enopyranoside (S14α)**. Obtained from glycal **S4,** in 20% isolated yield, after CC eluted with hex/EtOAc 95:5; mp: = 40.4−41.1 ºC, TLC (hex/EtOAc, 6:1 v/v): $R_f$ = 0.71; $[\alpha]_D$ = −96 º (*c* 1.0, $CHCl_3$); $^1$H NMR (400 MHz, $CDCl_3$) δ 6.07-6.04 (m, 2H, H-2, H-3), 4.99 (s, 1H, H-1), 4.95 (br s, 1H, H-4), 4.17 (dd, 1H, $J_{5ax,5eq}$ = 13.0 Hz, $J_{4,5ax}$ = 2.0 Hz, H-5ax), 3.83 (d, 1H, $J_{5ax,5eq}$ = 13.0 Hz, H-5eq), 3.77 (dt, 1H, $J_{1'a,1'b}$ = 9.4 Hz, $J_{1'a,2'}$ = 6.7 Hz, H-1'a), 3.48 (dt, 1H, $J_{1'a,1'b}$ = 9.4 Hz, $J_{1'b,2'}$ = 6.7 Hz, H-1'b), 2.10 (s, 3H, -OAc), 1.63-1.55 (m, 2H, H-2'), 1.37-1.23 (m, 18H, H-3' - H-11'), 0.88 (t, 3H, $J_{11',12'}$ = 6.8 Hz, H-12'); $^{13}$C NMR (100.6 MHz, $CDCl_3$) δ 170.7 (C=O, OAc), 131.1, 124.9 (C-3, C-2), 93.0 (C-1), 68.7 (C-1'), 63.4 (C-4), 61.2 (C-5), 31.9, 29.7, 29.7, 29.6, 29.6, 29.6, 29.4, 29.4,

26.2, 22.7 (C-2' - C-11'), 21.1 ($CH_3$, -OAc), 14.1 (C-12'); HRMS (*m/z*): Calcd for $[M+Na]^+$ 349.2349; Found: 349.2335 (error 4.2 ppm).

**Dodecyl 4-*O*-acetyl-2,3-dideoxy-β-L-*glycero*-pent-2-enopyranoside (S14β).** Obtained from glycal **S4,** in 7% isolated yield, after CC eluted with hex/EtOAc 95:5 as a syrup; TLC (hex/EtOAc, 6:1 v/v): $R_f$ = 0.78; $[\alpha]_D$ = −51 ° (*c* 1.1, $CHCl_3$). $^1H$ NMR (400 MHz, $CDCl_3$) δ 5.94 (br d, 1H, $J_{2,3}$ = 10.3 Hz, H-3), 5.87 (br d, 1H, $J_{2,3}$ = 10.3 Hz, H-2), 5.28 (br s, 1H, H-4), 4.93 (s, 1H, H-1), 3.88-3.74 (m, 3H, H-1'a, H-5ax, H-5eq), 4.47 (dt, 1H, $J_{1'a,1'b}$ = 9.3 Hz, $J_{1'b,2'}$ = 6.7 Hz, H-1'b), 2.07 (s, 3H, -OAc), 1.65-1.56 (m, 2H, H-2'), 1.39-1.21 (m, 18H, H-3' - H-11'), 0.88 (t, 3H, $J_{11',12'}$ = 6.7 Hz, H-12'); $^{13}C$ NMR (100.6 MHz, $CDCl_3$) δ 170.6 (C=O, OAc), 129.4 (C-2), 128.7 (C-3), 94.3 (C-1), 68.9 (C-1'), 65.0 (C-4), 60.2 (C-5), 31.9, 29.8, 29.7, 29.7, 29.6, 29.6, 29.4, 29.4, 26.2, 22.7 (C-2' - C-11'), 21.0 ($CH_3$, -OAc), 14.1 (C-12'); HRMS (*m/z*): Calcd for $[M+Na]^+$ 349.2349; Found: 349.2338 (error 3.3 ppm).

**Dodecyl 2,6-dideoxy-1-thio-α-L-*arabino*-hexopyranoside (2).** Dodecyl 3,4-di-*O*-benzyl-2,6-dideoxy-1-thio-α-L-*arabino*-hexopyranoside (**S7**, 0.082 g, 0.16 mmol) was dissolved in dried methanol (1 mL *per* 100 mg) and 10% Pd/C (20 mg) was carefully added. After air purge with $N_2$, reaction was kept under $H_2$ for 48 h. Reaction mixture was filtered over Celite, and the solvent was evaporated under reduced pressure. The residue was purified by CC eluted with hex/EtOAc 1:1 to afford the title compound as a white solid (0.048 g, 0.146 mmol) in 91% yield; mp: 54.4-55.4 ºC; TLC (Pet. ether/EtOAc, 2:3 v/v): $R_f$ =0.35; $[\alpha]_D$ = -165º (c1.0, $CHCl_3$); $^1H$ NMR (400 MHz, MeOD) δ 5.31 (d, 1H, $J_{1,2ax}$=5,80 Hz, H-1), 4.01 (dq, 1H, $J_{4,5}$=9.0, $J_{5,6}$= 6.3 Hz, H-5), 3.88 (ddd, 1H, $J_{2ax,3}$=12.4 Hz, $J_{3,4}$=9.0 Hz, $J_{3,2eq}$=4.6 Hz, H-3), 3.10 (t, 1H, $J_{3,4}$=$J_{4,5}$=9.0 Hz, H-4), 2.60-2.48 (m, 2H, H-1'), 2.18 (dd, 1H, $J_{2ax,2eq}$=13.0 Hz, $J_{3,2eq}$=4.6 Hz, H-2eq), 2.03 (ddd, 1H, $J_{2ax,2eq}$=13.0, $J_{2ax,3}$=12.4, $J_{1,2ax}$=5.80 Hz, H-2ax), 1.62-1.55 (m, 2H, H-2'), 1.38-1.31 (m, 2H,H-3'), 1.30 (d, 3H, $J_{5,6}$= 6.3 Hz, H-6), 1.27-1.22 (m, 16H, H-4' - H-11'), 0.87 (t, 3H, $J_{11',12'}$=6.5 Hz, H-12'); $^{13}C$ NMR (100.6 MHz, MeOD) δ 80.4 (C-1), 78.5 (C-4), 69.9 (C-3), 67.6 (C-5), 38.1 (C-2), 31.9 (C-1'), 31.1, 29.8, 29.7, 29.6, 29.6, 29.51, 29.3, 29.2, 28.9, 22.7 (C-2' - C-11'), 17.6 (C-6), 14.1 (C-12'); HRMS (*m/z*): Calcd for $[M+Na]^+$ 355.2277; Found: 355.2264 (error -3.6 ppm).

**Deacetylation procedure for the synthesis of dodecyl glycosides 2, 4/4b, 7 and 8.** Acetylated glycoside (**S8, S9, S10** or **S13,** 0.6 mmol) was dissolved in dried methanol (6 mL), and a 1M NaOMe solution in MeOH was added (0.1 mL *per* 100 mg) and stirred at room temperature for 2 h (**4**/**4b**) or 1 h 15 min (**7**/**8**). Neutralization with Amberlite (IR-120), followed by filtration and evaporation of the solvent gave the residue, which was submitted to column chromatography.

**Dodecyl 2,6-dideoxy-α-L-*lyxo*-hexopyranoside (4).** Obtained from **S8** in 99% yield after CC eluted with hex/EtOAc 1:1; mp: 54.4-56.9 ºC; TLC (EtOAc): $R_f$ = 0.56; $[\alpha]_D$ = −86º (c1.0, $CHCl_3$); $^1H$ NMR (400 MHz, MeOD) δ 4.83 (br d, 1H, $J_{1,2ax}$=3.8 Hz, H-1,partially under $H_2O$ signal), 3.92 (ddd, 1H, $J_{2ax,3}$= 11.8 Hz, $J_{2eq,3}$=5.1, H-3), 3.85 (q, 1H, $J_{5,6}$=6.6 Hz, H-5), 3.60 (td, 1H, H-1'a), 3.53 (br s, 1H, H-4), 3.36 (td, 1H, H-1'b), 1.90 (td, $J_{2ax,2eq}$ $J_{2ax,3}$=12.5 Hz, $J_{1,2ax}$=3.8 Hz, 1H, H-2ax), 1.74 (dd, 1H, $J_{1,2eq}$=5.1 Hz, H-2eq), 1.61-1.51 (m, 2H, H-2'), 1.40-1.23 (m, 18H, H-3' - H-11'), 1.22 (d, 3H, H-6), 0.90 (t, 3H, $J_{11,12}$= 7 Hz, H-12'); $^{13}C$ NMR (100.6 MHz, MeOD) δ 99.0 (C-1), 72.3 (C-4), 68.4 (C-1'), 67.5 (C-3), 67.0 (C-5),

33.4 (C-2), 33.1, 30.8, 30.7, 30.7, 30.6, 30.5, 27.4, 23.7 (C-2' - C-11'), 17.2 (C-6), 14.4 (C-12'). HRMS (*m/z*): Calcd for $[M+Na]^+$ 339.2506; Found: 339.2489 (error -4.9 ppm).

**Dodecyl 2,6-dideoxy-β-L-*lyxo*-hexopyranoside (4b).** Obtained from **S10** in 98% yield after CC eluted with hex/EtOAc 1:1; mp: 64.4-66.7 ºC; TLC (EtOAc): $R_f$ = 0.56; $[\alpha]_D$ = +17º (*c*1.0, $CHCl_3$); $^1$H NMR (400 MHz, MeOD) δ 4.45 (dd, 1H, $J_{1,2ax}$=9.9 Hz, $J_{1,2eq}$=1.7 Hz, H-1), 3.85 (td, 1H, H-1'a), 3.68 (ddd, 1H, $J_{2ax,3}$= 11.9 Hz, $J_{2eq,3}$=4.6, Hz, H-3), 3.51-3.42 (m, 3H, H-4, H-5, H-1'b), 1.81 (ddd, $J_{2ax,2eq}$=11.9 Hz, 1H, H-2eq), 1.69 (br q, 1H, $J_{2ax,3}$ $J_{1,2ax}$ $J_{2ax,2eq}$ =11.9 Hz, H-2ax), 1.60-1.53 (m, 2H, H-2'), 1.36-1.28 (m, 18H, H-3' - H-11'), 1.27 (d, 3H, $J_{5,6}$= 6.5 Hz, H-6), 0.90 (t, 3H, $J_{11,12}$= 6.8 Hz, H-12'), $^{13}$C NMR (100.6 MHz, MeOD) δ 101.6 (C-1), 71.9 (C-4), 71.4 (C-5), 70.1, 70.1 (C-3, C-1'), 35.4 (C-2), 33.1, 30.8, 30.8, 30.8, 30.7, 30.6, 30.5, 27.2, 23.8 (C-2' - C-11'), 17.1 (C-6), 14.5 (C-12'); HRMS (*m/z*): Calcd for $[M+Na]^+$ 339.2506; Found: 339.2491 (error -4.2 ppm).

**Dodecyl 2-deoxy-α-L-*threo*-pentopyranoside (7).** Obtained from **S9** in 93% isolated yield after CC eluted with hex/EtOAc 1:4 → EtOAc; mp: 52.8−54.2 ºC; TLC (EtOAc/hex, 3:1 v/v): $R_f$ =0.43; $[\alpha]_D$ = −62º (c 1.0, $CHCl_3$); $^1$H NMR (400 MHz, MeOD) δ 4.80 (br dd, 1H, $J_{1,2ax}$ = 3.5 Hz, $J_{1,2eq}$ = 1.7 Hz, H-1), 3.76 (br ddd, 1H, $J_{2ax,3}$ = 10.8 Hz, $J_{3,4}$ = 8.1 Hz, $J_{2eq,3}$ = 4.9 Hz, H-3), 3.63 (dt, 1H, $J_{1'a,1'b}$ = 9.3 Hz, $J_{1'a,2'}$ = 6.7 Hz, H-1'a), 3.59 (dd, 1H, $J_{5ax,5eq}$=10.1 Hz, $J_{4,5eq}$=4.5 Hz, H-5eq), 3.44 (t, 1H, $J_{5ax,5eq}$=$J_{5ax,4}$=10.1 Hz, H-5ax), 3.42-3.32 (m, 2H, H-4, H-1'b), 2.02 (br ddd, 1H, $J_{2ax,2eq}$=12.9 Hz, $J_{2eq,3}$=4.9 Hz, $J_{1,2eq}$=1.7 Hz, H-2eq), 1.62-1.52 (m, 3H, H-2ax, H-2'), 1.41-1.24 (m, 18H, H-3' - H-11'), 0.90 (t, 3H, $J_{11',12'}$ = 6.7 Hz, H-12'); $^{13}$C NMR (100.6 MHz, MeOD) δ 99.0 (C-1), 72.5 (C-4), 69.9 (C-3), 68.4 (C-1'), 63.6 (C-5), 38.5 (C-2), 34.1, 30.8, 30.7, 30.7, 30.7, 30.7, 30.6, 30.5, 27.4, 23.7 (C-2' - C-11'), 14.4 (C-12'); $^1$H NMR (400 MHz, $CDCl_3$) δ 4.84 (dd, 1H, $J_{1,2ax}$=3.3 Hz, $J_{1,2eq}$=1.7 Hz, H-1), 3.96-3.88 (m, 1H, H-3), 3.69 (dd, 1H, $J_{5ax,5eq}$=9.9 Hz, $J_{4,5eq}$=4.1 Hz, H-5eq), 3.63 (dt, 1H, $J_{1'a,1'b}$=9.3 Hz, $J_{1'a,2'}$=6.8 Hz, H-1'a), 3.59-3.46 (m, 2H, H-4, H-5ax), 3.34 (dt, 1H, $J_{1'a,1'b}$=9.3 Hz, $J_{1'b,2'}$=6.8 Hz, H-1'b), 2.51 (br s, 1H, OH), 2.11 (ddd, 1H, $J_{2ax,2eq}$=12.8 Hz, $J_{2eq,3}$=4.1 Hz, $J_{1,2eq}$=1.7 Hz, H-2eq), 1.72 (br s, 1H, OH), 1.64 (ddd, 1H, $J_{2ax,2eq}$=12.8 Hz, $J_{2ax,3}$=10.9 Hz, $J_{1,2ax}$=3.3 Hz, H-2ax), 1.60-1.52 (m, 2H, H-2'), 1.38-1.21 (m, 18H, H-3' - H-11'), 0.88 (t, 3H, $J_{11',12'}$ = 6.7 Hz, H-12'); $^{13}$C NMR (100.6 MHz, $CDCl_3$) δ 97.6 (C-1), 72.1 (C-4), 69.6 (C-3), 67.6 (C-1'), 62.1 (C-5), 37.3 (C-2), 31.9, 29.7, 29.6, 29.6, 29.6, 29.6, 29.5, 29.4, 26.2, 22.7 (C-2' - C-11'), 14.1 (C-12'); HRMS (m/z): Calcd. $[M+Na]^+$ 325.2349; Found: 325.2336 (error 4.1 ppm).

**Dodecyl 2-deoxy-β-L-*threo*-pentopyranoside (8).** Obtained from **S13** in 99% isolated yield after CC eluted with hex/EtOAc 1:4 → EtOAc. mp: 68.3-68.8 ºC; TLC (EtOAc/hex, 3:1 v/v): $R_f$ = 0.43; $[\alpha]_D$ = +72º (*c* 1.0, $CHCl_3$); $^1$H NMR (400 MHz, MeOD) δ 4.53 (dd, 1H, $J_{1,2ax}$ = 8.4 Hz, $J_{1,2eq}$=1.9 Hz, H-1), 3.90 (dd, 1H, $J_{5ax,5eq}$=11.5 Hz, $J_{4,5eq}$=4.7 Hz, H-5eq), 3.78 (dt, 1H, $J_{1'a,1'b}$=9.4 Hz, $J_{1'a,2}$= 6.7 Hz, H-1'a), 3.52 (br ddd, 1H, $J_{2ax,3}$=10.2 Hz, $J_{3,4}$=8.1 Hz, $J_{2eq,3}$ = 4.8 Hz, H-3), 3.44 (dt, 1H, H-1'b), 3.38 (ddd, 1H, $J_{4,5ax}$=9.0 Hz, $J_{3,4}$=8.1 Hz, $J_{4,5eq}$ = 4.7 Hz, H-4), 3.17 (dd, 1H, $J_{5ax,5eq}$=11.5 Hz, $J_{4,5ax}$=9.0 Hz, H-5ax), 2.11 (br ddd, 1H, $J_{2ax,2eq}$ = 12.8 Hz, $J_{2eq,3}$ = 4.8 Hz, $J_{1,2eq}$ = 1.9 Hz, H-2eq), 1.60-1.53 (m, 2H, H-2'), 1.48 (ddd, 1H, $J_{2ax,2eq}$ = 12.8 Hz, $J_{2ax,3}$ = 10.2 Hz, $J_{1,2ax}$ = 8.4 Hz, H-2ax), 1.37-1.25 (m, 18H, H-3' - H-11'), 0.90 (t, 3H, $J_{11',12'}$ = 6.7 Hz, H-12'); $^{13}$C NMR (100.6 MHz, MeOD) δ 101.5 (C-1), 72.1 (C-4), 71.7 (C-3), 70.1 (C-1'), 66.1 (C-5), 38.8 (C-2), 33.1, 30.8, 30.8, 30.8, 30.7, 30.7, 30.5, 30.5, 27.2, 23.8 (C-2' - C-11'), 14.5 (C-12'); $^1$H NMR (400 MHz, $CDCl_3$) $^1$H NMR (400 MHz, $CDCl_3$) δ 4.79 (t, 1H, $J_{1,2ax}$ = $J_{1,2eq}$ = 3.5 Hz, H-1), 4.15 (dd, 1H,

$J_{5ax,5eq}$ = 12.5 Hz, $J_{4,5eq}$ = 1.7 Hz, H-5eq), 3.80-3.72 (m, 2H, H-3, H-1'a), 3.64 (br s, 1H, H-4), 3.50-3.36 (m, 3H, H-5ax, OH, H-1'b), 2.21 (dt, 1H, $J_{2eq,2ax}$=14.2 Hz, $J_{1,2eq}$=$J_{2eq,3}$=3.5 Hz, H-2eq), 2.08 (br d, 1H, OH), 1.78 (dt, 1H, $J_{2ax,2eq}$=14.2 Hz, $J_{1,2ax}$=$J_{2ax,3}$=3.5 Hz, H-2ax), 1.63-1.56 (m, 2H, H-2'), 1.37-1.22 (m, 18H, H-3' - H-11'), 0.88 (t, 3H, $J_{11',12'}$ = 6.7 Hz, H-12'); $^{13}$C NMR (100.6 MHz, $CDCl_3$) δ 98.1 (C-1), 69.1 (C-4), 68.6 (C-1'), 67.9 (C-3), 60.8 (C-5), 32.3 (C-2), 31.9, 29.7, 29.6, 29.6, 29.6, 29.5, 29.4, 29.4, 26.2, 22.7 (C-2' - C-11'), 14.1 (C-12'); HRMS (m/z): Calcd. $[M+Na]^+$ 325.2349; Found: 325.2338 (error 3.5 ppm).

**Synthesis of dodecyl 6-deoxy-mannopyranosides**

**1,2,3,4-Tetra-*O*-acetyl-6-deoxy-α-L-mannopyranose (S17).** Commercial L-(+)-rhamnose monohydrate (1.0 g, 5.5 mmol) was dissolved in pyridine (10 mL) and acetic anhydride (6.9 mL, 73.1 mmol, 13 equiv.) was added dropwise at 0 ºC, followed by a catalytic amount of 4-(dimethylamino)pyridine. The reaction reached room temperature and was stirred overnight. Dichloromethane was then added, and the solution was washed with 2M HCl aq. solution and brine. The combined organic layers were dried with $MgSO_4$, filtered and concentrated under reduced pressure to give **S17** in quantitative yield as a very viscous colourless oil. TLC (hex/EtOAc, 2:1 v/v): $R_f$ **=** 0.42; $[\alpha]_D$ = −55º (c 1.0, $CHCl_3$); $^1$H NMR (400 MHz, $CDCl_3$) δ 6.02 (br d, 1H, $J_{1,2}$ = 1.1 Hz, H-1), 5.30 (dd, 1H, $J_{2,3}$ = 2.9 Hz, $J_{3,4}$ = 9.9 Hz, H-3), 5.25 (br dd, 1H, $J_{1,2}$ = 1.1 Hz, $J_{2,3}$ = 2.9 Hz, H-2), 5.13 (t, 1H, $J_{3,4}$ $J_{4,5}$ = 9.9 Hz, H-4), 3.94 (dq, 1H, $J_{4,5}$ = 9.9 Hz, $J_{5,6}$ = 6.2 Hz, H-5), 2.17 (s, 3H, -OAc), 2.16 (s, 3H, -OAc), 2.07 (s, 3H, -OAc), 2.01 (s, 3H, -OAc), 1.24 (d, 3H, $J_{5,6}$ = 6.2 Hz, H-6); $^{13}$C NMR (100.6 MHz, $CDCl_3$) δ 170.1(C=O, OAc), 169.9 (C=O, OAc), 169.8 (C=O, OAc), 168.4 (C=O, OAc), 90.6 (C-1), 70.4 (C-4), 68.8 (C-3), 68.7 (C-5), 68.6 (C-2), 20.9 ($CH_3$, -OAc), 20.8 ($CH_3$, -OAc), 20.8 ($CH_3$, -OAc), 20.7 ($CH_3$, -OAc), 17.4 (C-6).

**2,3,4-Tri-*O*-acetyl-6-deoxy-α-L-mannopyranosyl trichloroacetimidate (S18).** To a solution of L-rhamnose peracetate **S17** (2.29 g, 5.5 mmol) in DMF (20 mL) was added hydrazine acetate (1.77 g, 19.2 mmol, 3.5 equiv.) at room temperature. The reaction was stirred until complete consumption of the starting material (c.a. 96 h) and quenched by adding EtOAc. The solution was washed with brine and water, dried with $MgSO_4$, filtered and concentrated under reduced pressure to afford 2,3,4-tri-*O*-acetyl-6-deoxy-L-mannopyranose (1.36 g, 85%); TLC (hex/EtOAc, 2:1 v/v): $R_f$ = 0.21. The resulting product (100 mg, 0.35 mmol) and trichloroacetonitrile (104 µL, 1.04 mmol) were dissolved in dichloromethane (1 mL) at 0 ºC. Then, 1,8-diazabicyclo[5.4.0]undec-7-ene (16 µL, 0.11 mmol) was added and the reaction was allowed to reach room temperature. After 2 h, the solution was concentrated under reduced pressure and the residue was purified by column chromatography on silica gel (hex/EtOAc 3:1 to 2:1) to give glycosyl trichloroacetimidate **S18** as a syrup (133 mg, 89%). TLC (hex/EtOAc, 2:1 v/v): $R_f$ = 0.59; $^1$H NMR (400 MHz, $CDCl_3$) δ 8.74 (s, 1H, NH), 6.20 (d, 1H, $J_{1,2}$ = 1.6 Hz, H-1), 5.46 (dd, 1H, $J_{1,2}$ = 1.6 Hz, $J_{2,3}$ = 3.2 Hz, H-2), 5.37 (dd, 1H, $J_{2,3}$ = 3.2 Hz, $J_{3,4}$ = 10.1 Hz, H-3), 5.18 (t, 1H, $J_{3,4}$ $J_{4,5}$ = 10.1 Hz, H-4), 4.09 (dq, 1H, $J_{4,5}$ = 10.1 Hz, $J_{5,6}$ = 6.2 Hz, H-5), 2.19 (s, 3H, -OAc), 2.08 (s, 3H, -OAc), 2.01 (s, 3H, -OAc), 1.27 (d,3H, $J_{5,6}$ = 6.2 Hz, H-6); $^{13}$C NMR (100.6 MHz, $CDCl_3$) δ 169.9 (C=O, OAc), 169.9 (C=O, OAc), 169.8 (C=O, OAc), 159.9 (C-1'), 94.6 (C-1), 90.6 (C-2'), 70.3 (C-4), 69.3 (C-5), 68.8 (C-3), 68.4 (C-2), 20.8

($CH_3$, OAc), 20.8 ($CH_3$, OAc), 20.7 ($CH_3$, OAc), 17.5 (C-6). Spectroscopic characterization is in full agreement with the literature[16].

**Dodecyl 6-deoxy-α-L-mannopyranoside (6).** A solution of glycosyl trichloroacetimidate **S18** (68 mg, 0.157 mmol) and dodecan-1-ol (40 μL, 0.18 mmol, 1.1 equiv.) in dichloromethane (2 mL) was stirred in the presence of pre-activated 4 Å molecular sieves (100 mg) at room temperature for 30 min. The mixture was then cooled to 0 ºC and trimethylsilyl triflate (3 μL, 0.02 mmol, 0.1 equiv.) was added dropwise. After 2 h at 0 ºC, the reaction was quenched by addition of triethylamine until neutral pH and the solution was concentrated under vacuum to afford a residue containing dodecyl 2,3,4-tri-*O*-acetyl-6-deoxy-α-L-mannopyranoside as major product, as confirmed by NMR. $R_f$ = 0.72 (hex/EtOAc 3:2). The crude residue was then dissolved in dried methanol (0.5 mL), and a 1M NaOMe solution in MeOH (160 μL) was slowly added under vigorous stirring. The reaction mixture was kept under stirring at room temperature overnight and then neutralized with Amberlite (IR-120). Filtration and evaporation of the solvent gave a residue purified by column chromatography on silica gel eluted with EtOAc to afford glycoside **6** (37 mg, 71%); mp: 48.8−49.7 ºC; TLC (EtOAc): $R_f$ = 0.49; $[\alpha]_D = -49^o$ (*c* 1.0, $CHCl_3$); HRMS (*m/z*): Calcd. [M+Na] 355.2455; Exp. 355.2442 (error 3.6 ppm); NMR data was in full agreement with that described below for its enantiomer (compound **16**).

**Methyl 2,3,4-tri-*O*-benzyl-α-D-mannopyranoside (S20).** To a solution of methyl α-D-mannopyranoside (**S19)** (5 g, 25.7 mmol) in pyridine (175 mL), trityl chloride (12.9 g, 46.3 mmol, 1.8 equiv.) and DMAP (256 mg, 0.2 mmol, 0.08 equiv.) were added and the mixture kept under stirring at room temperature under nitrogen atmosphere for 5 h. Reaction mixture was washed with a saturated solution of $NaHCO_3$ (250 mL) and water (250 mL), and the aqueous phases extracted with EtOAc (3x250 mL). Organic phases were combined and dried with $MgSO_4$. After evaporation of the solvent, methyl 6-*O*-trytil-α-D-glucopyranoside was obtained as an oil with $R_f$ = 0.5 (EtOAc). This residue was then dissolved in DMF (350 mL) and kept at 0 ºC while NaH 60% (3.699 g, 154 mmol, 6 equiv.) was added. After dropwise addition of benzyl bromide (18.3 mL, 154 mmol, 6 equiv.), the reaction mixture was stirred at room temperature overnight, under nitrogen. Iced water (200 mL) was poured into the reaction mixture, which was then extracted with diethyl ether (3×100 mL). Organic layers were combined, washed with brine and dried with $MgSO_4$. After filtration, and evaporation under vacuum, the residue was the purified by column chromatography (hex/EtOAc 20:1) affording methyl 2,3,4-tri-*O*-benzyl-6-*O*-trityl-α-D-mannopyranoside ( 12.89 g, 71%); mp: 97.5-98.6 ºC; TLC (hex/EtOAc, 3:1 v/v): $R_f$ = 0.73; $[\alpha]_D = +10^o$ (*c*1.0, $CHCl_3$); $^1$H NMR (400 MHz, $CDCl_3$) δ 7.53-7.15 (m, 30H, Ph), 4.84 (part A of AB system1, 2H, C$\underline{H_2}$Ph, H-1), 4.76-4.69 (part B of AB system1, part A of AB system2, 2H, C$\underline{H_2}$Ph), 4.64 (s, 2H, C$\underline{H_2}$Ph), 4.27 (part B of AB system2, 1H, J=10.0 Hz, C$\underline{H_2}$Ph), 4.03 (t, 1H, $J_{3,4} = J_{4,5}$= 8.8 Hz, H-4), 3.88 (dd, 1H, $J_{2,3}$ = 3.1 Hz, H-3), 3.82 (br s, 1H, H-2), 3.77 (dd, 1H, $J_{5,6b}$ = 4.0 Hz, H-5), 3.52 (br d, 1H, $J_{6a,6b}$ = 9.7 Hz, H-6a), 3.37 (s, 3H, OMe), 3.27 (dd, 1H, H-6b); $^{13}$C NMR (100.6 MHz, $CDCl_3$) δ 146.8, 144.1 (Cq, C$\underline{Ph_3}$), 138.6, 138.6, 138.2 (Cq, Bn), 128.8, 128.3, 128.3, 128.1, 127.9, 127.7,127.6, 127.5, 127.4, 127.4, 127.1, 126.8 (Ph), 98.6 (C-1), 86.3 (C-2), 82.1 (Cq, -$\underline{C}Ph_3$), 80.2 (C-3), 75.4 ($\underline{C}H_2$Ph), 75.0 (C-4), 72.7 ($\underline{C}H_2$Ph), 71.7 (C-5), 63.0 (C-6), 54.5 ($OCH_3$); HRMS (*m/z*): Calcd for $[M+Na]^+$ 729.3187; Found: 729.3184 (error 0.4 ppm). 2,3,4-tri-*O*-benzyl-6-*O*-trityl-α-D-mannopyranoside (2.5 g) was dissolved in a solution of $I_2$ in

methanol (0.500g *per* 50 mL). The solution was stirred at 60 ºC for 3 h. After evaporation of the solvent, the resulting residue was dissolved in EtOAc (50 mL), washed with a saturated solution of sodium thiosulfate (50 mL), followed by water (50 mL). The organic phase was dried with $MgSO_4$, and after filtration, the solvent was evaporated under vacuum. Purification of the residue with column chromatography (hex/EtOAc 3:1) gave **S20** as a colourless oil (1.13 g, 57%); TLC (hex/EtOAc, 3:2 v/v): $R_f$= 0.40; $[\alpha]_D$ = +20º (c1.0, $CHCl_3$); $^1H$ NMR (400 MHz, $CDCl_3$) δ 7.38-7.24 (m, 15H, Ph), 4.94 (part A of AB system1, 1H, J= 11 Hz, C<u>H</u>$_2$Ph), 4.77 (part A of AB system2, 1H, J= 12 Hz, C<u>H</u>$_2$Ph), 4.70 (m, 5H, parts B of AB systems, C<u>H</u>$_2$Ph, H-1), 3.97 (t, 1H, $J_{3,4}$ = $J_{4,5}$= 9.6 Hz, H-4), 3.90 (dd, 1H, $J_{5,6a}$ = 2.7 Hz, $J_{6a,6b}$ = 9.7 Hz, H-6a), 3.84 (br dd, 1H, $J_{5,6b}$=2Hz, H-6b), 3.81-3.74 (m, 2H, H-2, H-3), 3.62 (ddd, 1H, H-5), 3.29 (s, 3H, OMe), 2.2 (br s, 1H, OH); $^{13}C$ NMR (100.6 MHz, $CDCl_3$) δ 138.4, 138.4, 138.2 (Cq, OBn), 128.4, 128.4, 128.4, 128.0, 127.8, 127.7,127.6 (Ph), 99.3 (C-1), 80.2 (C-2), 75.2 (C-3), 74.8 (<u>C</u>H$_2$Ph), 74.6 (<u>C</u>H$_2$Ph), 72.9 (C-4), 72.2 (<u>C</u>H$_2$Ph),72.0 (C-5), 62.4 (C-6), 54.8 ($OCH_3$). HRMS (*m/z*): Calcd for $[M+H]^+$ 465.2272; Found: 465.2252 (error 4.3 ppm).

**Methyl 2,3,4-Tri-*O*-benzyl-6-deoxy-α-D-mannopyranoside (S21).** To a solution of compound **S20** (0.710 g, 1.53 mmol) in toluene (10 mL), triphenylphosphane, (0.603 g, 2.30 mmol, 1.5 equiv.), imidazole (0.208 g, 3.06 mmol, 2 equiv.) and $I_2$ (, 0.584 g, 2.30 mmol, 1.5 equiv.), were added and the reaction was stirred at 65 ºC for 2 h, under nitrogen. Reaction mixture was cooled to room temperature, diluted with EtOAc (20 mL) and washed with saturated sodium thiosulfate solution (20 mL) and water (20 mL). The organic phase was dried with anhydrous $MgSO_4$, filtered and concentrated under vacuum. Column chromatography (hex/EtOAc 20:1) gave methyl 2,3,4-tri-*O*-benzyl-6-deoxy-6-iodo-α-D-mannopyranoside as a colourless oil (0.770 g, 88%); TLC (hex/EtOAc, 3:1 v/v): $R_f$ = 0.53; $[\alpha]_D$ = +27º (*c*1.0, $CHCl_3$); $^1H$ NMR (400 MHz, $CDCl_3$) δ 7.39-7.23 (m, 15H, Ph), 4.95 (part A of AB system, 1H, J= 11 Hz, C<u>H</u>$_2$Ph), 4.78-4.70 (m, 3H, C<u>H</u>$_2$Ph, H-1), 4.68-4.60 (m, 3H, C<u>H</u>$_2$Ph and part B of AB system), 3.83 (dd, 1H, $J_{3,4}$ = 8.8 Hz, $J_{2,3}$ = 2.6 Hz, H-3), 3.79-3.74 (m, 2H, H-2, H-5), 3.58-3.48 (m, 2H, H-4, H-6a), 3.36 (s, 3H, -OMe), 3.31 (m, 1H, H-6b); $^{13}C$ NMR (100.6 MHz, $CDCl_3$) δ 138.2, 138.2, 138.2 (Cq, Ph), 128.4, 128.4, 128.3, 128.0, 127.8, 127.6, 127.6 (Ph), 99.0 (C-1), 79.9 (C-3), 78.6 (C-2), 75.4 (<u>C</u>H$_2$Ph), 74.6 (C-5), 72.7 (<u>C</u>H$_2$Ph), 71.4 (C-4), 55.0 (OMe), 7.0 (C-6); HRMS (*m/z*): Calcd for $[M+Na]^+$ 597.1108; Found: 597.1107 (error -0.4 ppm). Methyl 2,3,4-tri-*O*-benzyl-6-deoxy-6-iodo-α-D-mannopyranoside (0.514 g, 0.89 mmol) was then dissolved in THF (20 mL), a 2M solution of $LiAlH_4$ in THF was added (7.2 mL; 14.3 mmol, 16 equiv.) and the reaction mixture was kept under reflux for 1 h. Reaction mixture was cooled down to 0 ºC, 0.3 mL of distilled water were added, followed by 0.3 mL of aqueous NaOH 15% and 0.9 mL of distilled water. This solution was stirred at room temperature for 15 min. Anhydrous $MgSO_4$ was then added, and after filtration the solvent was evaporated and the residue was purified with column chromatography eluted with hex/EtOAc 10:1, affording compound **S21** as a colourless oil (0.300g, 75%); TLC (hex/EtOAc, 5:1 v/v): $R_f$ = 0.35; $[\alpha]_D$ = +24º (*c*1.0, $CHCl_3$); $^1H$ NMR (400 MHz, $CDCl_3$) δ 7.38-7.24 (m, 15H, Ph), 4.95 (part A of AB system, 1H, J= 11 Hz, C<u>H</u>$_2$Ph), 4.78-4.70 (m, 3H, C<u>H</u>$_2$Ph, H-1), 4.65-4.60 (m, 3H, C<u>H</u>$_2$Ph, part B of AB system), 3.83 (dd, 1H, $J_{3,4}$ = 8.8 Hz, $J_{2,3}$ = 2.6 Hz, H-3), 3.78 (m, 1H, H-2), 3.70-3.59 (m, 2H, H-4, H-5), 3.29 (s, 3H, OMe), 1.34 (d, 3H, $J_{5,6}$ = 5.7 Hz, H-6); $^{13}C$ NMR (100.6 MHz, $CDCl_3$) δ 138.6, 138.6, 138.3 (Cq, Ph), 128.3, 128.4, 128.0, 127.9, 127.6, 127.6, 127.6, 127.5

(Ph), 99.0 (C-1), 80.4 (C-4), 80.1 (C-2) 75.4 (<u>C</u>$H_2$Ph), 74.6 (C-3) 72.8 (<u>C</u>$H_2$Ph), 72.1 (<u>C</u>$H_2$Ph), 67.8 (C-5), 54.6 (OMe), 18.0 (C-6); HRMS (*m/z*): Calcd for $[M+H]^+$ 449.2323; Found: 449.2323 (error 0.2 ppm).

**Dodecyl 6-deoxy-α-D-mannopyranoside (16).** To a solution of compound **S21** (0.230 g, 0.513 mmol) in dodecan-1-ol (2 mL), Amberlyst A15 beeds (0.038 g) were added and the reaction was stirred at 100 ºC for 8 h. Reaction mixture was diluted with dichloromethane, the resin was filtered off, and the reaction mixture was concentrated under vacuum. Column chromatography eluted with hex/EtOAc 20:1 afforded dodecyl 2,3,4-tri-O-benzyl-6-deoxy-α-D-mannopyranoside as a colourless oil (0.239 g, η=77%); TLC (hex/EtOAc, 5:1 v/v): $R_f$ =0.75; $^1$H NMR (400 MHz, $CDCl_3$) δ 7.38-7.26 (m, 15H, Ph), 4.94 (part A of AB system1, 1H, J= 10 Hz, C<u>H</u>$_2$Ph), 4.75 (part A of AB system2, 1H, J=13 Hz, C<u>H</u>$_2$Ph), 4.71 (part B of AB system2, 1H, J=13 Hz, C<u>H</u>$_2$Ph), 4.66-4.61 (m, 2H, H-1, part B of AB system1, C<u>H</u>$_2$Ph), 4.59 (br s, 2H, C<u>H</u>$_2$Ph), 3.86 (dd, 1H, $J_{3,4}$ = 9.0 Hz, $J_{2,3}$= 2.8 Hz, H-3), 3.77 (br s, 1H, H-2), 3.73-3.56 (m, 3H, H-1'a, H-4, H-5), 3.34-3.28 (m, 1H, H-1'b), 1.53-1.44 (m, 2H, H-2'), 1.33 (d, 3H, $J_{5,6}$ = 6.1 Hz, H-6), 1.30-1.18 (m, 18H, H-3' - H-11'), 0.88 (br t, 3H, $J_{11',12'}$=6.5 Hz); $^{13}$C NMR (100.6 MHz, $CDCl_3$) δ 138.6, 138.6, 138.4 (Cq, Ph), 128.3, 128.3, 128.0, 127.8, 127.5, 127.4 (Ph), 97.9 (C-1), 80.5 (C-4), 80.2 (C-2) 75.4 (<u>C</u>$H_2$Ph), 75.0 (C-3) 72.7 (<u>C</u>$H_2$Ph), 72.1 (<u>C</u>$H_2$Ph), 67.9 (C-5),67.5 (C-1'), 31.9 (C-2'), 29.6, 29.6, 29.4, 29.3 (C-3' - C-9'), 26.1 (C-10’), 22.7 (C11'), 18.0 (C.6), 14.1 (C-1'). Dodecyl 2,3,4-tri-*O*-benzyl-6-deoxy-α-D-mannopyranoside (0.175 g, 0.29 mmol) was dissolved in MeOH and 2 spatula tips of 10% Pd/C were added. The reaction mixture was stirred at room temperature for 2 h under hydrogen atmosphere. Catalyst was filtered off over Celite and washed with MeOH. The filtrate was concentrated and purified by column chromatography (hex/EtOAc 1:2) to afford compound **16** (0.090 g, 94%); mp: 48.7-50.0 ºC; TLC (hex/EtOAc, 1:2 v/v): $R_f$ = 0.49; $[\alpha]_D$ = +49º (*c*1.0, $CHCl_3$); $^1$H NMR (400 MHz, MeOD) δ 4.94 (br s, 1H, H-1), 3.77 (br s, 1H, H-2), 3.67-3.60 (m, 2H, H-3, H-1'a), 3.55 (qd, 1H, $J_{4,5}$ = 7.3 Hz, $J_{5,6}$ = 5.7 Hz, H-5), 3.40-3.33 (m, 2H, H-1'b, H-4), 1.60-1.52 (m, 2H, H-2'), 1.37-1.23 (m, 21H, H-3' - H-11', H-6), 0.88 (br t, 3H, $J_{11',12'}$=6.0 Hz, H-12'); $^{13}$C NMR (100.6 MHz, MeOD) δ 101.5 (C-1), 73.9 (C-4), 72.4 (C-3), 72.3 (C-2), 69.7 (C-5), 68.5 (C-1'), 33.1 (C-2'), 30.8, 30.8, 30.8, 30.7, 30.6, 30.5, 30.4 (C-3' - C-9'), 27.4 (C-10'), 23.8 (C11'), 18.0 (C.6), 14.5 (C-12'). HRMS (*m/z*): Calcd for $[M+Na]^+$ 355.2455; Found: 355.2441 (error 4.1 ppm).

**Synthesis of dodecyl 4-deoxy- and 4,6-dideoxy-D-*xylo*-hexopyranosides**

**Methyl 2,3-Di-*O*-benzyl-4,6-*O*-benzylidene-α-D-glucopyranoside (S23).** To a solution of methyl 4,6-*O*-benzylidene-α-D-glucopyranoside[17] (3 g, 10.6 mmol) in DMF (200 mL), NaH (60% oil suspension, 2.84 g, 42.4 mmol, 4 equiv.) was carefully added at 0 ºC and the reaction was stirred for 15 min. Then, benzyl bromide (5.1 mL, 42.4 mmol, 4 equiv.) was added and the reaction was stirred at room temperature overnight. The reaction was quenched by pouring it into cooled water (200 mL) and extracted with DCM (3×100 mL). Organic phases were combined, washed with saturated aqueous sodium hydrogen carbonate and dried with $MgSO_4$. After filtration and concentration under reduced pressure, the residue was purified by CC (hex→hex/EtOAc 5:1), affording compound **S23** as a syrup in 82% yield. TLC (hex/EtOAc, 2:1 v/v): $R_f$ = 0.63; $[\alpha]_D$ = -30 º (*c*1, $CHCl_3$); $^1$H NMR (400 MHz, $CDCl_3$) δ 7.55-7.27 (m, 15H, Ph), 5.55 (s, 1H, CH-Ph), 4.92 (part A, AB system, 1H, $CH_2$Ph), 4.85 (AB system, 2H, $CH_2$Ph), 4.70 (part B, AB system, 1H, $CH_2$Ph), 4.60 (d, 1H, $J_{1,2}$ = 2.9 Hz, H-1), 4.27 (dd, 1H, $J_{5,6a}$ = 4.0 Hz, $J_{6a,6b}$ = 10.0 Hz, H-6a),

4.05 (t, 1H, $J_{2,3}$=$J_{3,4}$=9.0 Hz, H-3), 3.83 (br td, 1H, $J_{4,5}$= 9.8 Hz, $J_{5,6b}$= 10.0 Hz, H-5), 3.71 (t, 1H, $J_{6a,6b}$ =10 Hz, H-6b), 3.63-3.54 (m, 2H, H-2, H-4), 3.40 (s, 3H, OMe); $^{13}$C NMR (100.6 MHz, $CDCl_3$) δ 138.7, 138.1, 137.4 (Cq, Ph), 128.9, 128.4, 128.3, 128.2, 128.1, 128.0, 127.9, 126.0 (Ph), 101.2 (CH-Ph), 99.2 (C-1), 82.1 (C-4), 79.1 (C-2), 78.6 (C-3), 75.3 ($\underline{C}H_2Ph$), 73.8 ($\underline{C}H_2Ph$), 69.0 (C-6), 62.3 (C-5), 55.3 ($OCH_3$); HRMS (*m/z*): Calcd. [$C_{28}H_{30}NaO_6$] 485.1935; Found 485.1928 (error 1.4 ppm).

**Methyl 2,3,6-tri-*O*-benzyl-α-D-glucopyranoside (S24).** To a solution of compound **S23** (3.00 g, 6.49 mmol) in acetonitrile (60 mL) containing 4Å molecular sieves (ca. 200 mg), sodium cyanoborohydride (2.037 g, 32.4 mmol, 5 equiv.) was added. Then, molecular iodine (3,781 g, 14.9 mmol, 2.3 equiv.) was added portionwise over 1 h (each time iodine was added the solution became orange and more iodine was added only when the solution became white). After completion of the starting material (3 h), the reaction mixture was diluted with dichloromethane (100 mL) and filtered over celite. The solution was washed with saturated aqueous sodium hydrogen carbonate and then with water. The organic phases were combined and dried with $MgSO_4$, filtered, and concentrated under reduced pressure to give a syrup purified by CC eluted with CyHex-EtOAc 4:1, affording compound **S24** as a colourless oil in 46% yield. TLC (hex/EtOAc, 3:1 v/v): $R_f$ = 0.43; $[\alpha]_D$ = +13 º (*c*1, $CHCl_3$); $^1$H NMR (400 MHz, $CDCl_3$) δ 7.35-7.27 (m, 15H, Ph), 4.99 (part A, AB system, $J_{A,B}$=11.0 Hz, 1H, $C\underline{H}_2Ph$), 4.78-4.70 (m, 2H, Part B, AB system1, -$C\underline{H}_2Ph$ and part A, AB system2, $J_{A,B}$=12.0 Hz, -$C\underline{H}_2Ph$), 4.67-4.60 (m, 2H, part B, AB system2, $J_{A,B}$=12.0 Hz, 2H, -$C\underline{H}_2Ph$, H-1), 4.57 (part A, AB system3, $J_{A,B}$=12.0 Hz, 1H, $C\underline{H}_2Ph$), 4.52 (part B, AB system3, $J_{A,B}$=12.0 Hz, 1H, $C\underline{H}_2Ph$), 3.79 (t, 1H, $J_{2,3}$ = $J_{3,4}$= 9.2 Hz, H-3), 3.73-3.64 (m, 3H, H-5, H-6a, H-6b), 3.59 (t, 1H, $J_{3,4}$ = $J_{4,5}$= 9.2 Hz, H-4), 3.52 (dd, 1H, $J_{1,2}$ = 4.0 Hz, $J_{2,3}$ = 9.2 Hz, H-2), 2.46 (br s, 1H, OH-4);$^{13}$C NMR (100.6 MHz, $CDCl_3$) δ 138.7, 137.9, 137.9 (Cq, Ph), 128.4, 128.3, 128.2, 128.0, 127.9, 127.8, 127.7, 127,5 (Ph), 98.0 (C-1), 81.3 (C-3), 79.5 (C-2), 75.3 ($\underline{C}H_2Ph$), 73.4 ($\underline{C}H_2Ph$), 73.0 ($\underline{C}H_2Ph$), 70.5 (C-4), 69.8 (C-5), 69.3 (C-6) 55.1 ($OCH_3$).

**Methyl 2,3,6-tri-*O*-benzyl-4-deoxy-α-D-*xylo*-hexopyranoside (S25).** Compound **S24** (2.28 g, 4.95 mmol) was dissolved in DCM (70 mL) and pyridine (0.9 mL, 11.39 mmol, 2.3 equiv.), and cooled to -10 ºC, under $N_2$. Trifluoromethanesulfonic anhydride (1.92 mL, 11.39 mmol, 2.3 equiv.) was then added dropwise to the stirred solution, which was warmed up to 0 ºC and stirred for 2 h 30 min. The reaction was quenched by addition of cool distilled water (100 mL), followed by extraction with dichloromethane (2 x 100 mL). The organic phase was dried with $MgSO_4$, filtered and evaporated under reduced pressure, affording methyl 2,3,6-tri-*O*-benzyl-4-*O*-trifluoromethanesulfonyl-α-D-glucopyranoside. The solid was dissolved in toluene (74 mL), and tetra-*n*-butylammonium borohydride (3.76 g, 14.85 mmol, 3 equiv.) was added. The reaction mixture was stirred at 85 ºC for 2 h, cooled to room temperature and then poured into ice cold water (100 mL). After extraction with dichloromethane (2x50 mL), the organic phase was washed with saturated aqueous sodium hydrogen carbonate (2x50 mL) and water (50 mL). The organic phases were combined and dried with $MgSO_4$, filtered, and concentrated under reduced pressure. The residue was purified by CC eluted with hex/EtOAc 10:1 to afford compound **S25** as a colourless oil in 83% yield. TLC (hex/EtOAc, 3:1 v/v): $R_f$ = 0.67; $[\alpha]_D$ = +26 º (*c*1, $CHCl_3$); $^1$H NMR (400 MHz, $CDCl_3$) δ 7.42-7.24 (m, 15H, Ph), 4.86-4.65 (m, 5H, $\underline{C}H_2Ph$, H-1), 4.54 (br s, 2H, $\underline{C}H_2Ph$), 3.97-3.90 (m, 2H, H-3, H-5), 3.50-3.44 (m, 3H, H-6a, H6b, H-2) 3.37 (s, 3H, OMe), 2.15 (s, 1H, OH-4), 2.05 (dd, 1H, $J_{4ax,4eq}$=

12.0 Hz, $J_{3,4eq}$=3 Hz, H-4eq), 1.50 (q, $J_{4ax,5}$=$J_{3,4ax}$=12 Hz, H-4ax); $^{13}$C NMR (100.6 MHz, $CDCl_3$) δ 138.8, 138.5, 138.1 (Cq, Ph), 128.4, 128.0, 127.8, 127.6, 127.5, (Ph), 99.2 (C-1), 80.4 (C-2), 75.3 (C-3), 75.4 ($\underline{C}H_2Ph$), 73.3 ($\underline{C}H_2Ph$), 72.5 ($\underline{C}H_2Ph$), 72.4 (C-6), 66.7 (C-5), 55.2 ($OCH_3$), 33.9 (C-4); HRMS (*m/z*): Calcd. for $[M+Na]^+$ 471.2142; Found 471.2144 (error 0.4 ppm).

**Dodecyl 4-deoxy-D-*xylo*-hexopyranoside (S26).** A solution of compound **S25** (1.498 g, 3.34 mmol) in dodecan-1-ol (11.2 mL, 50 mmol, 15 equiv.), containing Amberlyst A15 beads (0.250 g) was stirred at 100 ºC for 4.5 h. The reaction mixture was diluted with dichloromethane, the resin beads were filtered off, and the solution was concentrated under reduced pressure. The residue was taken up in methanol (15 mL) and, after addition of Pd/C 10% (50 mg) and inertization of the vessel with $N_2$, triethylsilane (1.8 mL, 5.63 mmol, 3.2 equiv.) was added dropwise. After 24 h, the catalyst was filtered off using celite, the solvent was evaporated under reduced pressure and the residue was purified by CC (petrol. ether/EtOAc 1:1 → EtOAc). The anomeric mixture **S26** (1.1:1 α/β) was obtained in 80% yield. TLC (EtOAc/toluene, 3:1 v/v): $R_f$ = 0.20; $^1$H NMR (400 MHz, MeOD) δ 4.81 (d, 1H, $J_{1,2}$=3.6 Hz, H-1α), 4.19 (d, 1H, $J_{1,2}$=7.9 Hz, H-1β), 3.91-3.80 (m, 3H, H-3α, H-5α, H1'aβ), 3.72 (td, 1H, $J_{1'a,1'b}$= 9 Hz, $J_{1'a,2'}$=7 Hz, H-1'aα), 3.65-3.48 (m, 7H, H-3β, H-5β, H-6α, H-6β, H1'bβ), 3.42 (td, 1H, H-1'bα), 3.32 (m, 1H, H-2α)*, 3.08 (t, 1H, $J_{1,2}$= $J_{2,3}$=7.9 Hz, H-2β), 1.93 (br dd, 2H, $J_{4ax,4eq}$= 13.1 Hz, $J_{4eq,5}$= $J_{3,4eq}$= 3.1 Hz, H-$4_{eq}$ α, H-$4_{eq}$ β), 1.69-1.67 (m, 4H, H-2' α, H-2'β), 1.42-1.25 (m, 38H, H-3' - H-11'α,β, H-$4_{ax}$α, H-$4_{ax}$β), 0.92-0.88 (m, 6H, H-12'α, H-12'β); $^{13}$C NMR (100.6 MHz, MeOD) δ 104.7 (C-1β), 100.7 (C-1α), 76.9 (C-2β), 75.6 (C-2α), 73.9, 72.3, 70.9, 70.0 (C-3α,C-4α, C-3β C-4β), 69.1 (C-1'α), 68.9 (C-1'β), 65.7 (C-6α), 65.6 (C-6β), 36.6 (-4α, C-4β), 33.2, 30.9, 30.9, 30.7, 30.6, 27.4, 27.2, 23.8 (C-2' α,β - C-11'α,β), 14.6 (C-12'α, C-12'β); *Signal under MeOH signal; HRMS (*m/z*): Calcd. for $[M+Na]^+$ 335.2455; Found 335.2451 (error 1.0 ppm).

**Dodecyl 4-deoxy-α-D-*xylo*-hexopyranoside (15).** An aliquote of the anomeric mixture **S26** was further purified by CC with $CHCl_3$:EtOH 95:5, to afford the pure alfa-anomer (**15**) for biological evaluation; mp: 79.6-80.7 °C; TLC ($CHCl_3$:EtOH, 9:1, v/v): $R_f$ = 0.3; $[\alpha]_D$ = +71º (*c*1, MeOH). $^1$H NMR (400 MHz, MeOD) δ 4.80 (d, 1H, $J_{1,2}$=3.6 Hz, H-1), 3.89-3.80 (m, 2H, H-3, H-5), 3.71 (td, 1H, $J_{1'a,1'b}$= 9 Hz, $J_{1'a,2'}$=7 Hz, H-1'a), 3.52 (br d, 2H, $J_{5,6}$=5 Hz, H-6a,b), 3.41 (td, 1H, H1'b), 3.30 (1H, H-2, partially under MeOH signal), 1.92 (ddd, 1H, $J_{4ax,4eq}$= 12 Hz, $J_{4eq,5}$=2 Hz, $J_{3,4eq}$= 5 Hz, H-$4_{eq}$), 1.71-1.55 (m, 2H, H-2'), 1.48-1.23 (m, 19H, H-3' to H-11', and H-$4_{ax}$), 0.92 (t, 3H, H-12'); $^{13}$C NMR (100.6 MHz, MeOD) δ 100.7 (C-1), 75.5 (C-2), 73.8, 69.9 (C-3, C-5), 69.0 (C-1'), 65.7 (C-6), 36.5 (C-4), 33.1, 30.8, 30.7, 30.6, 30.5, 27.4, 23.7 (C-2' to C-11'), 14.5 (C-12'); HRMS (*m/z*): Calcd. for $[M+Na]^+$ 335.2455; Found 335.2444 (error 3.0 ppm).

**Dodecyl 2,3-di-*O*-acetyl-4,6-dideoxy-α/β-D-*xylo*-hexopyranoside (S27, S28).** The anomeric mixture **8** (0.3176 g, 0.955 mmol) was dissolved in dichloromethane (10 mL) and pyridine (0.12 mL, 1.43 mmol, 1.5 equiv.), and cooled to -10 ºC, under $N_2$. Trifluoromethanesulfonic anhydride (0.24 mL, 1.43 mmol, 1.5 equiv.) was added dropwise to the solution kept under stirring, which was then warmed to 0 ºC and stirred for 1 h. The reaction was quenched by addition of cool distilled water (20 mL), followed by extraction with dichloromethane (2 x 20 mL). The organic phase was dried with $MgSO_4$, filtered and evaporated under reduced pressure at 25 ºC, affording a syrup. This intermediate triflate was dissolved in THF (8.3 mL), and a 2 M $LiAlH_4$ solution in THF (1.67 mL, 3.34 mmol, 3.5 equiv.) was added dropwise

at 0 ºC. After stirring 1 h at room temperature, the reaction was again cooled to 0 ºC. Slow addition of water (0.3 mL), followed by aqueous NaOH 15% (0.9 mL) and water (0.9 mL) gave a mixture kept under stirring for 15 min at room temperature. $MgSO_4$ was added to the resulting suspension and after filtration of the solids, the solution was concentrated under reduced pressure to give a syrup composed by an inseparable anomeric mixture of dodecyl 4,6-dideoxy-D-*xylo*-hexopyranoside, which was dissolved in pyridine (4 mL), and $Ac_2O$ (2 mL) followed by a spatula tip of DMAP. The reaction was stirred at room temperature overnight. The fully acetylated glycosides α and β could be separated in the TLC plate (hex/EtOAc 5:1). After co-evaporation of pyridine with toluene, the anomers were purified by column chromatography (hex/EtOAc 25:1) to afford the dodecyl 2,3-di-*O*-acetyl-4,6-dideoxy-α-D-*xylo*-hexopyranoside (**S27**, 116 mg, 30% yield) and dodecyl 2,3-di-*O*-acetyl-4,6-dideoxy-β-D-*xylo*-hexopyranoside (**S28**, 42 mg, 11% yield).

**Dodecyl 2,3-di-*O*-acetyl-4,6-dideoxy-α-D-*xylo*-hexopyranoside (S27)**. Colourless oil; TLC (hex/EtOAc, 5:1 v/v): $R_f$ = 0.50; $^1$H NMR (400 MHz, $CDCl_3$) δ 5.25 (ddd, 1H, $J_{2,3}$=10 Hz, $J_{3,4ax}$=11 Hz, $J_{3,4eq}$=4.9 Hz, H-3), 4.97 (d, 1H, $J_{1,2}$=3.4 Hz, H-1), 4.80 (dd, 1H, H-2), 4.05-3.95 (m, 1H, H-5), 3.65 (td, 1H, $J_{1'a,1'b}$= 10 Hz, $J_{1'a,2'}$=7 Hz, H-1'a), 3.36 (td, 1H, $J_{1'b,2'}$=7 Hz H-1'b), 2.18 (br dd, 1H, $J_{3,4eq}$=4.9 Hz, $J_{4eq,5}$= 1.2 Hz, H-4eq), 2.06 (s, 3H, OAc), 2.02 (s, 3H, OAc), 1.60-1.52 (m, 2H, H-2'), 1.41 (q, 1H, $J_{3,4ax}$= $J_{4,5}$= $J_{4eq,4ax}$=11 Hz, H-4ax), 1.35-1.21 (m, 18H, H-3' - H-11'), 1.18 (d, 3H, H-6, $J_{5,6}$=6 Hz), 0.87 (t, 3H, $J_{11',12'}$=6 Hz, H-12'); $^{13}$C NMR (100.6 MHz, $CDCl_3$) 170.5 (C=O, OAc), 170.3 (C=O, OAc), 96.3 (C-1), 72.2 (C-2), 68.2 (C-3), 68.0 (C-1'), 63.0 (C-5), 38.2 (C-4), 31.9, 29.7, 29.6, 29.4, 29.3, 26.1, 22.7 (C-2' to C-11'), 21.1 ($CH_3$, OAc), 20.9 ($CH_3$, OAc), 20.6 (C-6), 14.1 (C-12').

**Dodecyl 2,3-di-*O*-acetyl-4,6-dideoxy-β-D-*xylo*-hexopyranoside (S28)**. Colourless oil; TLC (hex/EtOAc, 5:1 v/v): $R_f$ = 0.39; $^1$H NMR (400 MHz, $CDCl_3$) δ 4.96 (ddd, 1H, $J_{2,3}$=10 Hz, $J_{3,4ax}$=10 Hz, $J_{3,4eq}$=5.3 Hz, H-3), 4.85 (dd, 1H, $J_{1,2}$=8 Hz, H-2), 4.35 (d, 1H, $J_{1,2}$=8 Hz, H-1), 3.85 (td, 1H, $J_{1'a,1'b}$= 10 Hz, $J_{1'a,2'}$=7 Hz, H-1'a), 3.68-3.58 (m, 1H, H-5), 3.45 (td, 1H, H-1'b), 2.12-2.06 (m, 1H, H-4eq), 1.71-1.42 (m, 5H, H-2', H-3', H-4ax), 1.41-1.17 (m, 19H, H-4' - H-11', H-6); 0.86 (t, 3H, H-12', $J_{11',12'}$=7 Hz); $^{13}$C NMR (100.6 MHz, $CDCl_3$) 170.1 (C=O, OAc), 169.8 (C=O, OAc), 98.8 (C-1), 72.6 (C-2), 71.2 (C-3), 69.8 (C-1'), 67.7 (C-5), 37.9 (C-4), 31.9, 29.7, 29.6, 29.5, 29.4, 25.9, 22.7 (C-2' to C-11'), 21.0 ($CH_3$, OAc), 20.9 ($CH_3$, OAc), 20.7 (C-6), 14.1 (C-12').

**Procedure for the deacetylation reaction to afford compounds 11 and 12.** The acetylated compound (**S27** or **S28**) is dissolved in methanol (100mg *per* mL), and a 1M solution of NaOMe in methanol (0.1 mL *per* 0.100 mg of substrate) is added. The reaction mixture is stirred for 2 h at room temperature. Neutralization with amberlite IR-120, filtration and evaporation of the solvent, afforded the deacetylated compounds.

**Dodecyl 4,6-dideoxy-α-D-*xylo*-hexopyranoside (11)**. Obtained from compound **S27** (0.099g, 0.25 mmol) in 96% yield (0.076 g); mp: 31.4-36.0 °C; TLC (toluene/EtOAc, 2:3 v/v): $R_f$ = 0.31; $[\alpha]_D$ = +103 º (*c*1, $CH_2Cl_2$); $^1$H NMR (400 MHz, MeOD) δ 4.70 (d, 1H, $J_{1,2}$=3.4 Hz, H-1), 3.90 (quint. d, 1H, $J_{5,6}$= $J_{4ax,5}$= 6 Hz, $J_{4eq,5}$=2 Hz, H-5), 3.77 (qd, 1H, $J_{2,3}$= $J_{3,4ax}$=10 Hz, $J_{3,4eq}$=5 Hz, H-3), 3.62 (td, 1H, $J_{1'a,1'b}$= 10 Hz, $J_{1'a,2'}$=7 Hz, H-1'a), 3.40 (td, 1H, $J_{1'b,2'}$=6 Hz, $J_{1'b,2'}$=7 Hz, H-1'b), 3.24 (dd, 1H, $J_{2,3}$=9 Hz, H-2), 1.91 (ddd,

1H, $J_{4ax,4eq}$= 12 Hz, H-$4_{eq}$), 1.65-1.51 (m, 2H, H-2'), 1.40-1.18 (m, 19H, H-3' - H-11', H-4ax), 1.13 (d, 3H, $J_{5,6}$=6 Hz, H-6), 0.87 (t, 3H, $J_{11',12'}$=6 Hz, H-12'). $^{13}$C NMR (100.6 MHz, MeOD) δ 100.8 (C-1), 75.5 (C-2), 69.1, 68.9 (C-1' and C-3), 65.2 (C-5), 42.3 (C-4), 33.1, 30.8, 30.7, 30.6, 30.5, 27.4, 23.8 (C-2' to C-11'), 21.2 (C-6), 14.5 (C-12'); HRMS (*m/z*): Calcd. for $[M+Na]^+$ 339.2506; Found 339.2509 (error -1.1 ppm).

**Dodecyl 4,6-dideoxy-β-D-*xylo*-hexopyranoside (12)**. Obtained from compound **S28** (0.030g, 0.075 mmol), in 94% yield (0.022 g); mp: 48.2-50.7 °C; TLC (tol/EtOAc, 2:3 v/v): $R_f$ = 0.31; $[\alpha]_D$ = -30 º (c0.6, $CH_2Cl_2$); $^1$H NMR (400 MHz, MeOD) δ 4.12 (d, 1H, $J_{1,2}$=7.9 Hz, H-1), 3.78 (td, 1H, $J_{1'a,1'b}$= 10 Hz, $J_{1'a,2'}$=7 Hz, H-1'a), 3.60-3.44 (m, 3H, H-1'b, H-3 and H-5), 3.01 (t, 1H, $J_{1,2}$=$J_{2,3}$=8 Hz, H-2), 1.89 (ddd, 1H, $J_{4ax,4eq}$= 13 Hz, $J_{3,4eq}$=2 Hz, H-$4_{eq}$), 1.58 (quint, 2H, $J_{1'a,2'}$=$J_{2'a,3'}$=7Hz, H-2'), 1.39-1.21 (m, 19H, H-3' - H-11', H-4ax), 1.13 (d, 3H, $J_{5,6}$=6 Hz, H-6), 0.87 (t, 3H, H-12', $J_{11',12'}$=6 Hz); $^{13}$C NMR (100.6 MHz, MeOD) δ 104.6 (C-1), 76.8 (C-2), 72.2 (C-3), 70-9 (C-1'), 69.1 (C-5), 42.1 (C-4), 33.1, 30.9, 30.8, 30.6, 30.5, 27.1, 23.8 (C-2' - C-11'), 21.3 (C-6), 14.5 (C-12'); HRMS (*m/z*): Calcd. for $[M+Na]^+$ 339.2506; Exp. 339.2495 (error 3.3 ppm).

**Synthesis of *C*-glycosides**

**1,3,4-Tri-*O*-acetyl-2,6-dideoxy-L-*arabino*-hexopyranose (S29).** Triphenylphosphane hydrobromide (0.10 mmol) and acetic acid (2.78 mmol) were added to a stirred solution of 3,4-di-*O*-acetyl-1,5-anhydro-2,6-dideoxy-L-*arabino*-hex-1-enitol (504 mg, 2.35 mmol) in dichloromethane (18.0 mL) at room temperature. The reaction mixture was stirred for 5 h at room temperature and then washed with a saturated solution of $NaHCO_3$, extracted with dichloromethane and dried with anhydrous sodium sulfate. After filtration, the organic phase was evaporated under reduced pressure to give a residue that was purified by column chromatography eluted with EtOAc/hex (1:4), affording the anomeric mixture **S29** (α,β = 8.3:1) as a colourless oil (213 mg, 33%). TLC (hex/EtOAc, 2:1 v/v): $R_f$ = 0.36; $^1$H NMR (400 MHz, $CDCl_3$) δ 6.19 (d, 1H, $J_{1\alpha,2\alpha}$= 2.08, H1α), 5.77 (dd, 1H, $J_{1\beta,2eq\beta}$= 1.8, $J_{1\beta,2ax\beta}$= 9.9, H-1β), 5.28 (ddd, 1H, $J_{3\alpha,2eq\alpha}$= 5.5, $J_{3\alpha,2ax\alpha}$= 11.29, H-3α), 5.07 – 4.99 (m, 1H, H-3β), 4.81 (t, 1H, $J_{4\alpha,3\alpha}$= $J_{4\alpha,5\alpha}$ =9.7, H-4α), 4.77 (t, 1H, $J_{4\beta,5\beta}$=$J_{4\beta,3\beta}$= 9.6, H-4β), 3.98-3.91 (m, 1H, H-5α), 3.65-3.59 (m, 1H, H-5β), 2.26 (dd, 1H, $J_{2ax\alpha,3\alpha}$= 11.29, $J_{2ax\alpha,2eq\alpha}$= 13.5, H-2axα), 2.34 (ddd, 1H, $J_{2ax\beta,3\beta}$= 5.0, $J_{2ax\beta,2eq\beta}$= 7.0, H-2axβ), 2.12 (s, 3H, $CH_3$, OAcα), 2.11 (s, 3H, $CH_3$, OAcβ), 2.07 (s, 3H, $CH_3$, OAcα), 2.06 (s, 3H, $CH_3$, OAcβ), 2.03 (s, 3H, $CH_3$, OAcα), 2.02 (s, 3H, $CH_3$, OAcβ), 1.93 (dd, 1H, H-2eqα), 1.83 (td, 1H, H-2eqβ), 1.24 (d, 3H, $J_{5,6}$= 6.2, H-6β), 1.20 (d, 3H, $J_{5,6}$= 6.2 Hz, H-6α); $^{13}$C NMR (100.6 MHz, $CDCl_3$) δ 170.4 (C=O, OAc α), 170.2 (C=O, OAc β), 170.1 (C=O, OAc α, β), 169.3 (C=O, OAc α), 168.9 (C=O, OAc β), 90.9 (C-1β), 90.8 (C-1α), 74.1 (C-4α), 73.6 (C-4β), 70.9 (C-5β), 70.2 (C-3β), 68.4 (C-5α), 68.2 (C-3α), 35.1 (C-2β), 34.1 (C-2α), 22.0 ($CH_3$, OAcα), 21.1 ($CH_3$, OAcα), 20.9 ($CH_3$, OAcβ), 20.8 ($CH_3$,OAcα e $CH_3$, OAcβ), 17.6 (C-6α), 17.5 (C-6β).

**1,3,4-Tri-*O*-acetyl-2-deoxy-D-*threo*-pentopyranose (S33).** Triphenylphosphane hydrobromide (0.10 mmol) and acetic acid (2.78 mmol) were added in sequence to a stirred solution of 1,5-anidro-3,4-di-*O*-acetyl-2-deoxy-D-*threo*-pent-1-enitol (473 mg, 2.36 mmol) in dichloromethane (18.0 mL) at room temperature. The reaction mixture was stirred for 5 h at room temperature. The reaction mixture was then

washed with a saturated solution of sodium hydrogen carbonate, extracted with dichloromethane and dried with anhydrous sodium sulfate. After filtration, the organic phase was evaporated, and the resulting residue was purified by column chromatography eluted with EtOAc/hex (1:4) to afford the anomeric mixture **S33** (α/β= 2.4:1) as a colourless oil (172 mg, 28%); TLC (hex/EtOAc, 2:1 v/v): $R_f$ =0.39; $^1$H NMR (400 MHz, $CDCl_3$) δ 6.13 (brs, 1H, $J_{1α,2α}$= 2.6, H-1α), 5.97 (t, 1H, $J_{1β,2β}$= 3.8, H-1β), 5.28 (ddd, 1H, $J_{3α,2eqα}$= 5.1, $J_{3α,2axα}$= 9.2, $J_{3α,4α}$= 13.7, H-3α), 5.00 – 4.90 (m, 2H, H-4α e H-3β), 4.85 (ddd, 1H, $J_{4β,5axβ}$= 4.7, $J_{4β,3β}$= 7.5, H-4β), 4.18 (dd, 1H, $J_{5eqβ,4β}$= 2.5, $J_{5axβ,5eqβ}$= 12.5, H-5eqβ), 3.94 (ddd, 1H, $J_{5eqα,4α}$= 4.9, $J_{5axα,5eqα}$= 11.6, H-5eqα), 3.73 (dd, 1H, $J_{5axα,4α}$= 9.19, H-5axα), 3.64 (dd, 1H, $J_{5aβ,4β}$= 4.73, H-5axβ), 2.31 – 2.21 (m, 2H, H-2axα, H-2axβ), 2.13 (s, 3H, $CH_3$, OAcα), 2.10 (s, 9H, $CH_3$, OAcα e $CH_3$, OAcβ), 2.07 (s, 6H, $CH_3$, OAcα e $CH_3$, OAcβ), 1.98-1.87 (m, 2H, H-2eqα, H-2eqβ); $^{13}$C NMR (100.6 MHz, $CDCl_3$) δ 170.1 (C=O, OAc α), 170.0 (C=O, OAc α, β), 169.8 (C=O, OAc β), 169.3 (C=O, OAc α, β), 90.8 (C-1α), 90.5 (C1β), 68.7 (C4α), 67.9 (C-3α), 67.6 (C-4β), 66.7 (C-3β), 61.8 (C-5α), 61.0 (C-5β), 33.2 (C-2α), 30.3 (C-2β), 21.1 ($CH_3$, -OAcβ), 21.0 ($CH_3$, OAcα, $CH_3$, OAcβ), 20.9 ($CH_3$, OAcα).

**1,3,4,6-Tetra-*O*-acetyl-2-deoxy-β-D-*arabino*-hexopyranose (S34).** 2-deoxy-D-*arabino*-hexopyranose (502 mg, 3.06 mmol) was dissolved in pyridine (5 mL). After addition of acetic anhydride (1.4 mL, 14.68 mmol) and DMAP (catalytic), the reaction mixture was stirred at room temperature for 1 h. After completion, the reaction was washed with a solution of 2M HCl. The organic phase was extracted with dichloromethane and dried with anhydrous sodium sulfate. After filtration, the organic phase was evaporated under reduced pressure, resulting in a residue that was purified by column chromatography eluted with EtOAc/hex (1:2), affording compound **S34** as colourless oil (1.0 g, 100%); TLC (hex/EtOAc, 2:1 v/v): $R_f$ = 0.43; $^1$H NMR (400 MHz, $CDCl_3$) δ 5.80 (br d, 1H, $J_{1,2ax}$= 9.1, H-1), 5.11 – 5.01 (m, 2H, H-3, H-4), 4.33 (dd, 1H, $J_{6a,5}$= 4.5, $J_{6a,6b}$= 12.3 Hz, H-6a), 4.08 (dd, 1H, $J_{6b,5}$= 2.3 Hz, H-6b), 3.76 (ddd, 1H, $J_{4,5}$=9.5 Hz, H-5), 2.35 (br dd, 1H, $J_{2ax,2eq}$= 12.5 Hz, $J_{2ax,3}$=4.9 Hz, H-2ax), 2.12 (s, 3H, $CH_3$, OAc), 2.09 (s, 3H, $CH_3$, OAc), 2.05 (s, 6H, $CH_3$, OAc), 1.91 (m, 1H, H-2eq); $^{13}$C NMR (100.6 MHz, $CDCl_3$) δ 170.7 (C=O, OAc), 170.1 (C=O, OAc), 169.8 (C=O, OAc), 168.8 (C=O, OAc), 91.1 (C1), 72.8 (C5), 70.2 (C4), 68.2 (C3), 62.0 (C6), 34.7 (C2), 21.0 ($CH_3$, -OAc), 20.9 ($CH_3$, -OAc), 20.8 ($CH_3$, -OAc), 20.7 ($CH_3$, -OAc).

**General procedure for the allylation reaction.** Allyltrimethylsilane (0.61 mL, 3.84 mmol) and boron trifluoride etherate (0.95 mL, 7.68 mmol) were added successively to a solution of the appropriate *O*-acetyl 2-deoxy sugar (**S29, S33** or **S34**, 500 mg) in dry acetonitrile (3.0 mL) under nitrogen atmosphere at 0 ºC. The reaction mixture was stirred for 15 min at 0 ºC, then washed with a saturated solution of $NaHCO_3$. The organic phase was extracted with dichloromethane and dried with anhydrous sodium sulfate. After filtration, the organic phase was evaporated under reduced pressure to give a residue that was purified by column chromatography.

**3-(3,4-Di-*O*-acetyl-2,6-dideoxy-α-L-*arabino*-hexopyranosyl)prop-1-ene (S30).** By applying the above-mentioned procedure to **S29**, compound **S30** was obtained as a colourless oil (409 mg, 73%); TLC (hex/EtOAc, 2:1 v/v): $R_f$ = 0.61; $^1$H NMR (400 MHz, $CDCl_3$) δ 5.84 – 5.73 (m, 1H, H-2), 5.16 – 5.09 (m, 3H, H-1b, H-1a, H-3'), 4.69 (t, 1H, $J_{4',3'}$=$J_{4',5'}$= 7.6, H-4'), 4.04 – 3.98 (m, 1H, H-1'), 3.80 (qd, 1H, $J_{5',6'}$= 6.53, $J_{5',4'}$= 13.3, H5'), 2.52 (Part AX of the system ABX, 1H, $J_{3a,2}$= 7.9, $J_{3a,3b}$= 14.5, H-3a), 2.30 (Part BX

of the system ABX, 1H, $J_{3b,2}$= 6.8, H-3b), 2.07 (s, 3H, $CH_3$, OAc), 2.05 (s, 3H, $CH_3$, OAc), 1.99 (dd, 1H, $J_{2'ax,2'eq}$= 13.9, $J_{2'ax,3'}$= 9.3, H-2'ax), 1.87 – 1.80 (m, 1H, H-2'eq), 1.23 (d, 3H, H-6'); $^{13}$C NMR (100.6 MHz, $CDCl_3$) δ 170.3 (C=O, OAc), δ 170.1 (C=O, OAc), 134.2 (C-2), 117.5 (C-1), 73.6 (C-4'), 69.7 (C-1'), 69.1 (C-3'), 68.2 (C-5'), 36.7 (C-3), 32.4 (C-2'), 21.1 ($CH_3$, OAc), 21.0 ($CH_3$, OAc), 17.5 (C-6').

**3-(3,4-Di-*O*-acetyl-2-deoxy-α-D-*threo*-pentopyranosyl)prop-1-ene (S35).** By applying the above mentioned procedure to compound **S33**, product **S35** was obtained as a colourless oil (284 mg, 61%); TLC (hex/EtOAc, 2:1 v/v): $R_f$ = 0.54; $^1$H NMR (400 MHz, $CDCl_3$) δ 5.84 - 5.73 (m, 1H, H-2), 5.14 – 5.07 (m, 2H, H-1b, H-1a), 5.01 - 4.87 (m, 2H, H-3' and H-4'), 4.08 (dd, 1H, $J_{5'eq,4'}$=5.3 Hz, $J_{5'eq,5'ax}$= 11.2 Hz, H-5'e), 3.48 (m, 1H, $J_{1',3}$= 7.7 Hz, $J_{1',2'ax}$= 6.3 Hz, $J_{1',2'eq}$= 1.7 Hz, H-1'), 3.22 (t, 1H, $J_{5'ax,4'}$=10.4 Hz, H-5'ax), 2.37 – 2.30 (m, 1H, H-3a), 2.27 – 2.20 (m, 1H, H-3b), 2.15 (dd, 1H, $J_{2'eq,3'}$= 4.9 Hz, H-2'eq), 2.05 (s, 3H, $CH_3$, OAc), 2.04 (s, 3H, $CH_3$, OAc), 1.46 (td, 1H, $J_{2'ax,3'}$= 11.4Hz =$J_{2'eq,2'ax}$, $J_{1',2'ax}$=6.8 Hz, H-2'ax); $^{13}$C NMR (100.6 MHz, $CDCl_3$) *δ* 170.5 (C=O, OAc), δ 170.2 (C=O, OAc), 133.7 (C-2), 117.7 (C-1), 75.4 (C-1'), 71.8 (C-3'), 70.0 (C-4'), 67.1 (C-5'), 39.7 (C-3), 35.8 (C-2'), 21.1 ($CH_3$, OAc), 20.9 ($CH_3$, OAc).

**3-(3,4,6-Tri-*O*-acetyl-2-deoxy-α-D-*arabino*-hexopyranosyl)prop-1-ene (S36).** By applying the above mentioned procedure to compound **S34**, product **S36** was obtained as a colourless oil (407 mg, 86%); TLC (hex/EtOAc, 2:1 v/v): $R_f$ = 0.22; $^1$H NMR (400 MHz, $CDCl_3$) δ 5.81 – 5.73 (m, 1H, H-1), 5.16 – 5.07 (m, 3H, H1b, H-1a, H-3'), 4.85 (t, 1H, $J_{4,3}$=$J_{4,5}$= 7.8 Hz, H-4'), 4.39 (dd, 1H, $J_{6'b,5'}$= 5.9 Hz, $J_{6'b,6'a}$= 11.4 Hz, H-6'b), 4.09-4.05 (m, 2H, H-1', H-6a'), 3.92 (ddd, 1H, H-5'), 2.55 – 2.48 (m, 1H, H-3a), 2.31 – 2.28 (m, 1H, H-3b), 2.06 (s, 3H, $CH_3$, OAc), 2.06 (s, 6H, $CH_3$, OAc), 2.01 – 1.97 (m, 1H, H-2'ax), 1.86 – 1.84 (m, 1H, H-2'eq); $^{13}$C NMR (100.6 MHz, $CDCl_3$) δ 170.3 (C=O, OAc), δ 169.6 (C=O, OAc), δ 169.5 (C=O, OAc), 133.8 (C-2), 117.3 (C-1), 70.5 (C-5'), 69.5 (C-1'), 68.4 (C-3', C-4'), 61.8 (C-6'), 36.5 (C-3), 31.8 (C-2'), 20.7 ($CH_3$, OAc), 20.5 ($CH_3$, OAc), 20.5 ($CH_3$, OAc).

**General procedure for the metathesis reaction.** The 2$^{nd}$ generation Grubbs-Hoveyda catalyst (5%, 1.86 mmol) was added to a solution of allyl glycoside (3.5 mmol) and 1-undecene (3.97 mL, 19.30 mmol, 5.5 equiv.) in dichloromethane (18 mL). The reaction was refluxed under nitrogen for 24 h. After the reaction was complete (TLC), the solvent was evaporated, and the residue directly purified by column chromatography to afford the product.

**(2*E*)-/(2*Z*)-1-(3,4-Di-*O*-acetyl-2,6-dideoxy-α-L-*arabino*-hexopyranosyl)dodec-2-ene (S31).** By applying the above-mentioned procedure to compound **S30**, the unseparable (2*E*)/(2*Z*) mixture in a 4/1 ratio **S31** was isolated as a colourless oil (766 mg, 75%); TLC (hex/EtOAc, 5:1 v/v): $R_f$ = 0.50; (*E*)-Isomer: $^1$H NMR (400 MHz, $CDCl_3$) δ 5.54, 5.50 (part AXY of ABXY system, each t, 1H, $J_{1a,2}$= $J_{1b,2}$= 6.5 Hz, $J_{2,3}$= 15.2 Hz, H-2); 5.37, 5.33 (part BXY of ABXY system, each t, 1H, $J_{3,4a}$= $J_{3,4b}$= 7.1 Hz, H-3),5.11 (td, 1H, $J_{3',2eq'}$= 4.7, $J_{3',2ax'}$= 13.0, H-3'), 4.68 (t, 1H, $J_{3',4'}$= $J_{4',5'}$= 7.8, H-4'), 3.97 – 3.91 (m, 1H, H-1'), 3.81 – 3.75 (qd, 1H, H-5'), 2.48 – 2.40 (m, 1H, H-1a), 2.27-2.20 (m, 1H, H-1b), 2.06 (s, 3H, $CH_3$, OAc), 2.04 (s, 3H, $CH_3$, OAc), 2.01 – 1.95 (m, 3H, H-4a, H-4b and H-2'ax), 1.87-1.77 (m, 1H, H-2'eq), 1.26 (br s, 14H, H-5 - H-11), 1.21 (d, 3H, $J_{5',6'}$= 6.5 Hz, H-6'), 0.88 (t, 3H, $J_{11,12}$= 6.4, H-12); $^{13}$C NMR (100.6 MHz, $CDCl_3$) δ 170.2 (C=O, OAc), 170.1 (C=O, OAc), 133.8 (C-2), 125.2 (C-3), 74.0 (C-4'), 70.5 (C-1'), 69.2 (C-3'), 68.0 (C-5'), 35.4 (C-1), 32.6 (C-4), 32.4 (C-2'), 31.9, 29.6, 29.4, 29.3, 29.2, 22.7 (C-5 to C-11),

21.1 ($CH_3$, -OAc), 20.9 ($CH_3$, -OAc), 17.6 (C6'), 14.1 (C-12); HRMS (*m/z*): Calcd. for $[M+Na]^+$ 405.2611; Found: 405.2621 (error: -2.3 ppm).

**(2*E*)-1-(3,4-Di-*O*-acetyl-2-deoxy-α-D-*threo*-pentopyranosyl)dodec-2-ene (S37).** By applying the above-mentioned procedure to **S35**, compound **S37** was obtained as a colourless oil (780 mg, 74%); TLC (hex/EtOAc, 5:1 v/v): $R_f$ = 0.51; $^1$H NMR (400 MHz, $CDCl_3$) δ 5.50, 5.46 (part AXY of ABXY system, each t, 1H, $J_{1a,2}$= $J_{1b,2}$= 6.3 Hz, $J_{2,3}$= 15.3 Hz, H-2); 5.37, 5.33 (part BXY of ABXY system, each t, 1H, $J_{3,4a}$= $J_{3,4b}$= 7.4 Hz, H-3), 5.00 – 4.86 (m, 2H, H-3' and H-4'), 4.07 (dd, 1H, $J_{5'eq,4}$= 5.4 Hz, H-5'eq), 3.44-3.36 (m, 1H, H-1'), 3.21 (t, 1H, $J_{5'ax,4}$=$J_{5'ax,5'eq}$= 10.6, H-5'ax), 2.31 – 2.24 (m, 1H, H-1a), 2.19 – 2.10 (m, 2H, H-1b, H-2'ax), 2.04 (s, 3H, $CH_3$, OAc), 2.03 (s, 3H, $CH_3$, OAc), 2.01 – 1.96 (m, 2H, H-4a, H-4b), 1.47 – 1.38 (m, 1H, H-2'eq), 1.26 (br s, 14H, H-5 - H-11), 0.88 (t, 3H, $J_{11,12}$= 6.4, H-12); $^{13}$C NMR (100.6 MHz, $CDCl_3$) δ 170.5 (C=O, OAc), 170.2 (C=O, OAc), 134.1 (C-2), 124.7 (C-3), 76.0 (C-1'), 72.0 (C-3'), 70.1 (C-4'), 67.1 (C-5'), 35.4 (C-1), 32.6 (C-4), 32.0 (C-2'), 29.6, 29.5, 29.3, 29.2, 22.7 (C-5 to C-11), 21.1 ($CH_3$, -OAc), 20.9 ($CH_3$, -OAc), 14.1 (C-12); HRMS (*m/z*): Calcd. for $[M+H]^+$ 369.2636; Found: 369.2621 (error: 4 ppm).

**(2*E*)-/(2*Z*)-1-(3,4,6-Tri-*O*-acetyl-2-deoxy-α-D-*arabino*-hexopyranosyl)dodec-2-ene (S38).** By applying the abovementioned procedure to **S36**, compound **S38** was obtained as colourless oil (407 mg, 77%); TLC (hex/EtOAc, 4:1 v/v): $R_f$ = 0.26; *E*/*Z* 4:1 (2*E*)-isomer: $^1$H NMR (400 MHz, $CDCl_3$) δ 5.55, 5.51 (part AXY of ABXY system, each t, 1H, $J_{1a,2}$= $J_{1b,2}$= 6.4 Hz, $J_{2,3}$= 15.5 Hz, H-2); 5.37, 5.33 (part BXY of ABXY system, each t, 1H, $J_{3,4a}$= $J_{3,4b}$= 7.2 Hz, H-3), 5.17 – 5.11 (m, 1H, H-3'), 4.88 (t, 1H, $J_{3',4'}$= $J_{4',5'}$= 7.7, H-4'), 4.37 (dd, 1H, $J_{6a',5'}$= 6.0, $J_{6a',6b'}$= 12.0, H-6'a), 4.11 – 3.97 (m, 2H, H-6'b, H-1'), 3.91 – 3.87 (m, 1H, H-5'), 2.44 (m, 1H, $J_{1a,2}$= 7.6, $J_{1a,1b}$= 14.6, H-1a), 2.27 – 2.20 (m, 1H, H-1b), 2.09 (s, 3H, $CH_3$, OAc), 2.06 (s, 3H, $CH_3$, OAc), 2.05 (s, 3H, $CH_3$, OAc), 2.02 – 1.97 (m, 3H, H-4a, H-4b and H-2'ax), 1.91 – 1.80 (m, 1H, H-2'eq), 1.26 (m, 14H, H-5 - H-11), 0.88 (t, 3H, $J_{11,12}$= 6.4, H-12); $^{13}$C NMR (100.6 MHz, $CDCl_3$) δ 170.8 (C=O, OAc), 170.1 (C=O, OAc), 169.8 (C=O, OAc), 134.0 (C-2), 124.8 (C-3), 70.6 (C-1'), 70.5 (C-5'), 68.9 (C-3' e C-4'), 62.4 (C-6'), 35.4 (C-1), 32.6 (C-4), 31.7 (C-2'), 29.6, 29.4, 29.3, 29.2, 22.7 (C-5 - C-11), 21.0 ($CH_3$, OAc), 20.8 ($CH_3$, OAc), 20.8 ($CH_3$, OAc), 14.1 (C-12); HRMS (*m/z*): Calcd. for $[M+H]^+$ 441.2847; Found: 441.2830 (error: 3.8 ppm).

**General procedure for hydrogenation and deacetylation.** A solution containing the alkene (**S31**, **S37** or **S38**, 2.0 mmol) and 10% Pd/C (10-20% w/w) in EtOAc (6 mL) was stirred under hydrogen atmosphere for 3 h, at room temperature. After completion of the starting material, the mixture was filtered through Celite and the solvent was removed under reduced pressure. After dissolution of the residue in MeOH (10 mL *per* g), a solution of 1% NaOMe in MeOH (1 mL *per* g) was added. The mixture was stirred at room temperature for 1 h. Neutralization with Amberlite (IR-120) was followed by filtration and evaporation of the solvent to give a residue, which was submitted to column chromatography to afford the respective glycosyl dodecane. The intermediate acetylated products (**S32**, **S39** and **S40)** were purified on a short chromatography column for intermediate product characterization.

**1-(3,4-Di-*O*-acetyl-2,6-dideoxy-α-L-*arabino*-hexopyranosyl)dodecane (S32).** Colourless oil (769 mg, quantitative yield); TLC (hex/EtOAc, 5:1 v/v): $R_f$ = 0.70; $^1$H NMR (400 MHz, $CDCl_3$) *δ* 5.10 (ddd,

1H, $J_{2'ax,3'}$= 9.36 Hz, $J_{2'eq,3'}$= 5.1 Hz, $J_{3',4'}$= 7.8 Hz, H-3'), 4.67 (t, 1H, $J_{3',4'}$= $J_{4',5'}$= 7.8, H-4'), 3.92 (m, 1H, H-1'), 3.74 (qd, 1H, $J_{5',6'}$= 6.5, H-5'), 2.06 (s, 3H, $CH_3$, OAc), 2.03 (s, 3H, $CH_3$, OAc), 1.95 (ddd, 1H, $J_{2'ax,2'eq}$=13.3, H-2'ax), 1.88 – 1.77 (m, 2H, H-2'eq, H-1a), 1.47 – 1.42 (m, 1H, H-1b), 1.26 (br s, 20H, H-2 - H-11), 1.21 (d, 3H, H-6'), 0.88 (t, 3H, $J_{12,11}$= 6.4, H-12); $^{13}$C NMR (100.6 MHz, $CDCl_3$) $\delta$ 170.3 (C=O. OAc), 170.1 (C=O, OAc), 74.2 (C-4'), 70.6 (C-1'), 69.5 (C-3'), 67.6 (C-5'), 33.4 (C-2'), 31.9, 31.7, 29.68, 29.65, 29.60, 29.57, 29.40, 29.36, 26.0, 22.7 (C-1 - C-11), 17.7 (C-6'), 14.1 (C-12); HRMS (*m/z*): Calcd. for $[M+H]^+$ 385.2949; Found: 385.2938 (error: 2.7 ppm).

**1-(2,6-Dideoxy-α-L-*arabino*-hexopyranosyl)dodecane (3).** Isolated in quantitative yield (840 mg); mp: 68.6-69.9 ºC; TLC (hex/EtOAc, 1:1 v/v): $R_f$ = 0.37; $^1$H NMR (400 MHz, $CDCl_3$) δ 3.91 – 3.88 (m, 1H, H-1'), 3.71 (ddd, 1H, $J_{2'ax,3'}$= 11.4 Hz, $J_{2'eq,3'}$= 5.0 Hz, H-3'), 3.48 (qd, 1H, H-5'), 2.94 (t, 1H, $J_{3',4'}$= $J_{4',5'}$= 8.7 Hz, H-4'), 1.90 (ddd, 1H, $J_{2eq',1'}$= 1.4 Hz, $J_{2ax',2eq'}$= 11.2 Hz, H-2'e), 1.83 (m, 1H, H-1a), 1.73 (td, 1H, $J_{2ax',1'}$= 5.9 Hz, H-2'ax), 1.50-1.30 (m, 21H, H-1b, H-2 - H-11), 1.26 (d, 3H, $J_{5',6'}$= 6.3 Hz, H-6'), 0.93 (t, 3H, $J_{12,11}$= 6.5 Hz, H-12). $^{13}$C NMR (100.6 MHz, $CDCl_3$) δ 77.8 (C-4'), 72.4 (C-1'), 68.9 (C-5'), 68.7 (C-3'), 36.2 (C-2'), 31.7 (C-2 to C-11), 30.6 (C-1), 29.4(0), 29.3(6), 29.3, 29.1, 25.9, 22.3 (C-2 to C-11), 17.3 (C-6'), 13.1 (C-12). HRMS (*m/z*): Calcd. for $[M+H]^+$ 301.2737; Found: 301.2736 (error: -0.4 ppm).

**1-(3,4,6-Tri-*O*-acetyl-2-deoxy-α-D-*arabino*-hexopyranosyl)dodecane (S39).** Colourless oil (768 mg, quantitative yield); TLC (hex/EtOAc, 2:1 v/v): $R_f$ = 0.35 (EtOAc/hex 1:2); $^1$H NMR (400 MHz, $CDCl_3$) δ 5.14 (ddd, 1H, $J_{3',4'}$= 7.7 Hz, $J_{2'ax,3'}$= 12.8, $J_{2'eq,3'}$= 4.9 Hz, H-3'), 4.87 (t, 1H, $J_{3',4'}$= $J_{4',5'}$= 7.7 Hz, H-4'), 4.37 (dd, 1H, $J_{6'a,5'}$= 5.9 Hz, $J_{6'a,6'b}$= 9.1 Hz, H-6'a), 4.08 (dd, 1H, $J_{6'b,5'}$= 3.3 Hz, H-6'b), 3.99 (td, 1H, $J_{1',1}$= 9.4, $J_{1',2'}$= 4.6, H-1'), 3.88 – 3.83 (m, 1H, H-5'), 2.09 (s, 3H, $CH_3$, OAc), 2.06 (s, 3H, $CH_3$ OAc), 2.05 (s, 3H, $CH_3$, OAc), 1.96 (dt, 1H, $J_{2ax',2eq'}$= 13.6, $J_{2ax',3'}$= 9.1, H-2'ax), 1.90 – 1.83 (m, 1H, H-2'eq), 1.81 – 1.74 (m, 1H, H-1a), 1.45 – 1.39 (m, 1H, H-1b), 1.26 (m, 20H, H-2 to H-11), 0.88 (t, 3H, $J_{12,11}$= 6.6, H-12); $^{13}$C NMR (100.6 MHz, $CDCl_3$) δ 170.8 (C=O, OAc), 170.2 (C=O, OAc), 169.8 (C=O, OAc), 70.8 (C-1'), 70.2 (C-5'), 69.1 (C-3'), 69.0 (C-4'), 62.4 (C-6'), 33.0 (C-2'), 31.9, 31.7, 29.64, 29.59, 29.3, 25.7, 22.7 (C-1 to C-11), 21.1 ($CH_3$, OAc), 20.83 ($CH_3$, OAc), 20.80 ($CH_3$, OAc), 14.1 (C-12); HRMS (*m/z*): Calcd. $[M+Na]^+$ 465.2823; Found: 465.2803 (error: 4.3 ppm).

**1-(2-Deoxy-α-D-*arabino*-hexopyranosyl)dodecane (14).** Isolated in quantitative yield (808 mg); mp: 91.1-91.7 ºC; TLC (EtOAc): $R_f$= 0.27; $^1$H NMR (400 MHz, $CDCl_3$) δ 3.97 – 3.95 (m, 1H, H-1'), 3.81 (dd, 1H, $J_{6'a,5'}$= 2.6, $J_{6'a,6'b}$= 11.7, H-6'a), 3.78 – 3.73 (m, 1H, H-3'), 3.70 (dd, 1H, $J_{6'b,5'}$= 5.7, H-6'b), 3.42 (ddd, 1H, $J_{5',4'}$= 8.7, H-5'), 3.22 (t, 1H, $J_{3',4'}$= $J_{4',5'}$= 8.7, H-4'), 1.90 (ddd, 1H, $J_{2'ax,2'eq}$= 11.3, $J_{2'eq,3'}$= 4.8, $J_{2'eq,1'}$= 2.0, H-2'eq), 1.83 (t, 1H, $J_{1a,1'}$= $J_{1a,1b}$= 9.0, H-1a), 1.73 (td, 1H, $J_{2ax',1'}$= 5.8, H-2'ax), 1.46 (brs, 1H, H-1b), 1.32 (br s, 20H, H-2 to H-11), 0.93 (t, 3H, $J_{12,11}$= 6.4, H-12); $^{13}$C NMR (100.6 MHz, $CDCl_3$) δ 73.8 (C-5'), 72.3 (C-1'), 72.2 (C-4'), 68.9 (C-3'), 61.8 (C-6'), 35.9 (C-2'), 30.6 (C-1), 31.7, 29.4, 29.3, 29.2, 29.1, 25.8, 22.3 (C-2 to C-11), 13.0 (C-12). HRMS (*m/z*): Calcd. $[M+Na]^+$ 339.2506; Found: 339.2504 (error: -0.7 ppm).

**1-(3,4-Di-*O*-acetyl-2-deoxy-α-D-*threo*-pentopyranosyl)dodecane (S40).** Colourless oil (770 mg, quantitative yield); TLC (hex/EtOAc, 2:1 v/v): $R_f$= 0.62; $^1$H NMR (400 MHz, $CDCl_3$): 5.01 – 4.87 (m, 2H, H-3' and H4'), 4.07 (dd, 1H, $J_{5'eq,4'}$= 5.4 Hz, $J_{5'ax,5'eq}$= 11.2 Hz, H-5'eq), 3.40 – 3.35 (m, 1H, H-1'), 3.21

(t, 1H, $J_{5'ax,4'}$= 10.8 Hz, H-5'ax), 2.13 (ddd, 1H, $J_{1',2'ax}$ = 1.5 Hz, $J_{2'ax,2'eq}$= 12.6 Hz, $J_{2'ax,3'}$= 6.4, H-2'ax), 2.05 (s, 3H, $CH_3$,-OAc), 2.04 (s, 3H, $CH_3$, OAc), 1.59 – 1.50 (m, 1H, H-1a), 1.47 – 1.39 (m, 2H, H-2'eq, H-1b), 1.26 (m, 20H, H-2 to H-11), 0.89 (t, 3H, $J_{12,11}$= 6.4, H-12); $^{13}$C NMR (100.6 MHz, $CDCl_3$): 170.5 (C=O, OAc), 170.1 (C=O, OAc), 76.1 (C-1'), 72.0 (C-3'), 70.2 (C-4'), 67.0 (C-5'), 36.3 (C-2'), 35.3 (C-1), 31.9, 29.64, 29.62, 29.53, 29.49, 29.3, 25.4, 22.7 (C-2 to C-11), 21.1 ($CH_3$, OAc), 20.8 ($CH_3$, OAc), 14.1 (C-12); HRMS (*m/z*): Calcd. for $[M+Na]^+$ 393.2611; Found: 93.2602 (error: 2.4 ppm).

**1-(2-Deoxy-α-D-*threo*-pentopyranosyl)dodecane (21).** Isolated in quantitative yield (570 mg); mp: 69.8-70.7 ºC; TLC (hex/EtOAc, 1:1 v/v): $R_f$= 0.24; $^1$H NMR (400 MHz, $CDCl_3$) δ 3.88 (dd, 1H, $J_{5'eq,4'}$= 5.2 Hz, $J_{5'eq,5'ax}$=10.7 Hz, H-5'eq), 3.50 – 3.44 (m, 1H, H-1'), 3.37 – 3.31 (m, 2H, H-3', H-4'), 3.08 (t, 1H, $J_{5'eq,5'ax}$=$J_{5'ax,4'}$= 10.7, H-5'ax), 1.97 (ddd, 1H, $J_{1',2'ax}$= 1.5, $J_{2'ax,2'eq}$= 12.9, $J_{2'ax,3'}$= 6.5, H-2'ax), 1.56 – 1.44 (m, 2H, H-1a, H-1b), 1.32 (m, 20H, H-2 to H-11), 1.28 – 1.22 (m, 1H, H-2'eq), 0.93 (t, 3H, $J_{12,11}$= 6.8, H-12); $^{13}$C NMR (100.6 MHz, $CDCl_3$) δ 76.5 (C-4'), 72.6 (C-1'), 71.7 (C-'), 69.7 (C-5'), 39.1 (C-2'), 35.3 (C-1), 31.7, 29.3(8), 29.3(5), 29.3(1), 29.2(9), 25.3, 22.3 (C-2 to C-11), 13.0 (C-12); HRMS (*m/z*): Calcd. for $[M+Na]^+$ 309.2400; Found: 309.2400 (error: 0.0 ppm).

**1-(2,6-Dideoxy-α-D-*arabino*-hexopyranosyl)dodecane (10).** TsCl (602 mg, 3.16 mmol) in pyridine (2 mL) was added to a solution of **14** (500 mg, 1.56 mmol) in pyridine (4.0 mL) at 0 ºC and the reaction mixture was stirred at room temperature for 4 h. The solvent was co-evaporated with toluene under reduced pressure, and the resulting residue was dissolved in dry THF (23.0 mL), and $LiAlH_4$ (2M in THF, 8.5 mL) was added. The reaction was stirred under reflux for 2 h. When the reaction was complete, the solution was cooled to 0 ºC and water (0.5 mL) was added dropwise, followed by aqueous NaOH 15% (1.0 mL). The solution was stirred for 15 min at room temperature and then dried with anhydrous sodium sulfate. After filtration and evaporation of the solvent, the resulting residue was purified by column chromatography eluted with EtOAc/hex 1:2, yielding product **10** (198 mg, 42%); mp: 62.1-63.3 ºC; TLC (EtOAc): $R_f$ = 0.58; $^1$H NMR (400 MHz, $CDCl_3$) δ 3.93 – 3.90 (m, 1H, H-1'), 3.78 (ddd, 1H, $J_{2ax',3'}$= 9.0 Hz, $J_{2eq',3'}$= 5.1 Hz, H-3'), 3.47 (qd, 1H, $J_{5',6'}$= 5.9 Hz, $J_{5',4'}$= 9.1 Hz, H-5'), 3.06 (t, 1H, $J_{3',4'}$= $J_{4',5'}$= 9.1 Hz, H-4'), 2.61 (br s, 1H, OH), 1.91 (dd, 1H, $J_{2ax',1'}$= 4.8 Hz, $J_{2ax',2eq'}$= 12.5 Hz, H-2'ax), 1.83 – 1.75 (m, 2H, H-2'eq and H-1a), 1.43 – 1.20 (m, 21H, H-1b, H-2 - H-11), 0.88 (t, 3H, $J_{12,11}$= 6.3, H-12); $^{13}$C NMR (100.6 MHz, $CDCl_3$) δ 78.8 (C-4'), 72.8 (C-1'), 69.9 (C-3'), 68.3 (C-5'), 36.3 (C-2'), 31.0 (C-1), 29.7, 29.6, 29.4, 26.2, 22.7 (C-2 - C-11), 18.3 (C-6'), 14.1 (C-12); HRMS (*m/z*): Calcd. $[M+H]^+$ 301.2737; Found: 301.2730 (error: 1.3 ppm).

### 3.2. Biological studies

The bactericidal activity was evaluated with **s**trains of *Bacillus cereus* ATCC 14579 and *Staphylococcus aureus* ATCC 25923, obtained from the American Type Culture Collection (Rockville, MD). The bacterial cultures were maintained at lyophilized state or stored at -80 ºC in Müller-Hinton broth (MH) supplemented with 20% glycerol (Sigma-Aldrich). Overnight cultures of bacteria were grown in 50 mL Erlenmeyer flasks, with orbital shake at 200 rpm at 30 ºC. MIC determination was made by a modified micro-dilution method according to CLSI guidelines[18]. Briefly, overnight culture (17 h) of all

bacterial strains were diluted to 0.5 McFarland units in Müller-Hinton broth. In sterile 96-well plates (Sarstedt), 150 μL of culture media were added and then supplemented with the compound in study by serial dilution. Finally, the desired wells were inoculated with 10 μL of a 1/10 dilution of the 0.5 McFarland bacterial suspension. Microplates were incubated at 35 °C, with orbital shaking, taking absorbance readings in all wells at intervals of 10 min, for up to 24 h (Anthos Zenith 3100 Microplate Multimode Detector). The lowest concentration at which no bacterial growth was observed is considered the minimal inhibitory concentration (MIC). Aliquots of 5 μL from every well were transferred to Müller-Hinton Agar (MHA) for determination of minimum bactericidal concentration (MBC). Bacterial growth was assessed at 17 h, and MBC was considered the lowest concentration of compound where no bacterial growth was observed. All measurements are the average of a minimum of 3 independent experiments.

The *In vitro* time-killing curves were obtained as follows: the compounds were added to an exponential phase culture of *B. cereus* in Müller-Hinton containing about $10^6$, $10^7$, and $10^8$ cells *per* mL. Bacteria were then incubated at 35 ºC, and 5 μL aliquots were withdrawn at different intervals and where diluted in a previously premade microplate. The samples were serial diluted in 1/10 of the previous concentration, and then, a 10 μL droplet was transferred to MHA and bacterial growth was evaluated. The number of surviving bacteria, expressed as the number of colony forming units (CFU), were counted after an overnight incubation at 30 ºC. For all the experiments, sample homogeneity and methodology validation were performed by $OD_{600nm}$, as a measure of cellular density, for all sixteen replicates.

The study of bacterial metabolic response to compound **1**, was performed using Phenotype MicroArrays (Biolog). The impact of compound **1** was assessed in the metabolism of 95 different carbon sources by *B. cereus* ATCC 14579 strain. All the solutions used were supplied by the manufacturer. The PM solutions B-F where sterilized in autoclave at 121 ºC during 15 min, and afterwards stored at 4 ºC until use. The PM additive solution was prepared by mixing these solutions (10 mL of solutions B, C, E and F and 30 mL of solution D and water). The bacterial growth media was used according to instructions provided by the manufacturer. The bacterial suspension, for PM Inoculating Fluid, was prepared by centrifuging the overnight pre-inoculum (at 3000 *g*, 4 ºC for 5 min collecting and resuspending the cells in IF-0a, and finally adjusting the bacterial suspension to 0.5 McFarland units. Finally, 100 μL of the PM inoculating fluid, was added to each well of 4 PM1 microplates (Compound **1** at the final concentrations of 16, 8, 4 and 0 μg $mL^{-1}$). The microplates were sealed with parafilm, shielded from light and incubated at 35 ºC with orbital shaking. The compound was added at 1 h of incubation, in order to stabilize and allow bacterial cells to adapt to the new media. The reduction of tetrazolium violet associated to NADH produced by the consumption of each tested carbon source, was monitored by $OD_{590nm}$ at time 0 min, 30 min, 60 min after incubation, and then, hourly for 27 h (Anthos Zenith 3100 Microplate Multimode Detector). The time-dependent series for each tested carbon source was classified by the acquisition periods, corresponding to: 1) initial phase of response to the compound **1** (from 0 to 5 h of incubation); 2) late phase of response (from 16 h to 27 h of incubation). After data normalization, results were divided into five categories depending on the effect shown: 1) highly inhibitory effect, when the difference between the effect of the compound **1** and control was higher than 5σ ($OD_{590nm} > 5σ$); 2) inhibitory effect, when the difference observed was between 5σ and 2σ ($5σ > OD_{590nm} > 2σ$); 3) no effect, when bacterial

growth was observed in the absence of compound ($OD_{590nm} > 2\sigma$) and the difference between absence and presence of compound was less than 2σ; 4) no growth, when value with and without the presence of compound was less than 2σ ($OD_{590nm} < 2\sigma$); 5) potentiation effect, when value without the compound was less than 2σ ($OD_{590nm} < 2\sigma$) and the difference of value between the presence and absence of compound was superior to σ. The hits classified as category 1, 2 or 5, were correlated to the biological pathways described at "Kyoto Encyclopedia of Genes and Genomes" (KEGG) database[19].The biological pathways were then ordered by the number of inhibitory hits *per* pathway. The metabolic reconstruction was performed using graph theory for hierarchical superimposition of the pathways based at the nonparametric Spearman's rank association coefficients (edges) between hits (nodes – according to KO classification system). The SPSS (SPSS Statistics for Windows, SPSS Inc.) software was used for data normalization and determination of nonparametric association coefficients and Cytoscape (Cytoscape, U.S. NIGMS) for the hierarchical representation (Supplementary Fig. 9).

The construction of mutant libraries of *B. cereus* ATCC 14579 was initiated by the preparation of electro-competent cells, by growing the bacterial cells in an Erlenmeyer flask containing tryptic soy broth (TSB, Difco Laboratories), and placed in an incubator at 37 ºC and 200 rpm, until the bacterial suspension reached a maximum OD of 0.45 – 0.46 measured at 600 nm. The suspension was then incubated during 1 h in 5% glycine or DL-threonine and 250 mM sucrose at 37 °C at 200 rpm. Cells were washed five times in electroporation buffer (250 mM sucrose, 1 mM Hepes, 1 mM $MgCl_2$, 10% glycerol, pH 7.0) and concentrated 150-fold.

Electroporation was performed at 25 µF using a Bio-Rad Gene Pulser X-cell apparatus (Bio-Rad laboratories, U.S.). Electroporation was carried out in 1 mm electroporation cuvettes, at 4 ºC, with 50 µL cells combined with 500 ng of plasmid, in order to respect cells/DNA proportions. It was used voltage of 20 $kVcm^{-1}$ with 200 ohms of resistance. After electroporation, cell suspensions were immediately diluted with 1 mL of TSB supplemented with 250 mM sucrose, 5 mM $MgCl_2$, 5 mM $MgSO_4$ and incubated for 2 h at 37 °C, 200 rpm to allow expression of antibiotic resistance markers. Aliquots were spread onto tryptic soy agar (TSA, Difco Laboratories) supplemented with appropriate antibiotic. Transformants harbouring antibiotic resistance were counted following overnight incubation.

For the constrution of *in vivo* mutant library by random insertion of transposon Tn917 in *B. cereus* ATCC 14579, transposon mutagenesis was performed by obtaining a stationary-phase culture of modified *B. cereus* (pLTV1) cultured overnight at 28 °C in Lysogeny broth (LB) containing tetracycline (50 µg *per* mL), erythromycin (1 µg *per* mL), and chloramphenicol (10 µg *per* mL) (Sigma-Aldrich). This culture was diluted 1:800 in prewarmed (43 °C) LB broth that contained erythromycin (1 µg *per* mL) and chloramphenicol (5 µg *per* mL). Then, it was incubated at 43 °C for 24 h with shaking at 200 rpm. The above procedure was repeated one more time. Transposon mutants were subsequently selected on LB agar containing erythromycin (1 µg *per* mL) and chloramphenicol (5 µg *per* mL) and incubated at 37 °C, resulting in two libraries of $10^6$ mutants each.

The validation of insertion site of Tn917 carried by plasmid pLVT1 was performed by southern blotting, in the two pools of mutants. Briefly, chromosomal DNA from both *B. cereus* ATCC 14579 wild

and modified strains, as well as from both generated mutant libraries, were isolated using the CTAB method[20]. After digestion with *Eco*RI the DNA of the mutant libraries and control strains were resolved in an agarose gel 0.8 %. The DNA transfer proceeded overnight, and hybridization was performed at 40 ºC with DNA probe (5.1 kb *Eco*RI restriction fragment of Tn917), labelled with digoxigenin-dUTP, at a concentration of 25 ng *per* mL. The membrane was revealed with NBT-BCIP.

For the transporter systems knockout mutant library, twenty-eight knockout mutants based in the isogenic system *B. subtilis* 168 on main ABC and PTS systems were purchased from National BioResource Project (NIG, Japan): *Bacillus subtilis*. Supplementary Table 5 identifies the individual mutants and respective MIC and MBC values obtained for compound **1**.

### 3.3. Computational Studies

DFT calculations were performed with Gaussian 09 software[21]. The Perdew– Burke–Ernzerhof functional (PBE0), which uses 25% exchange and 75% correlation weighting, was used in all calculations[22]. Geometries were optimized without symmetry constraints and obtained using the 6-311G** basis set for all atoms[23]. Frequency calculations were performed to assess the nature of optimized geometries, and zero imaginary frequency was obtained for all minima. All geometries were optimized in the corresponding media, using the integral equation formalism of the polarizable continuum solvation model (IEFPCM)[24] on the electron density (SMD)[25]. Dielectric constants (bulk relative permittivities) of $\varepsilon$=4.711 (for chloroform) and $\varepsilon$=32.613 (methanol) were used for solvent specification.

All molecular mechanics/molecular dynamics (MM/MD) simulations were performed using the GROMACS software package (version 4.0.7)[26,27]. The GROMOS 54A7 force field[28] was used, which already includes the corrections to phosphatidylcholine parametrization originally implemented in the GROMOS 53A6 force field[29]. For carbohydrates, the GROMOS 56A$_{CARBO}$ force field[30] was used. The modelled glycoside and phospholipid (DMPC) molecules were built using PyMOL[31]. All simulations were started from pre-equilibrated systems solvated with adequate number (see below) of explicit SPC water molecules[32]. A tetragonal simulation box was used, applying periodic boundary conditions in all three dimensions with the minimum image convention, and ensuring that the membrane pore or lipid bilayer system could not see the respective periodic image along the membrane normal. Each simulation replicate was started by applying different sets of initial velocities taken from a Maxwellian distribution at 308 K. The equations of motion were numerically integrated with a time step of 2 fs, with conformations being saved every 10 ps for analysis purposes. Non-bonded interactions were treated using a twin-range method with cutoffs of 8 Å and 14 Å, updating the neighbor pair list every step and every 5 steps, respectively. A reaction field was used to treat electrostatic interactions larger than 14 Å, using a relative dielectric constant of 54.0[33]. All simulations were performed in the isothermal-isobaric (NPT) ensemble. Solute and solvent were separately coupled to external heat baths using the v-rescale algorithm[34] at 308 K with a $\tau_p$ = 0.1 ps. Pressure was kept constant at 1 bar using a Berendsen's semi-isotropic coupling[35], with isothermal compressibility of 4.5 × $10^{-5}$ $bar^{-1}$ and a $\tau_p$ = 5.0 ps (unless stated otherwise). All glycoside

and/or phospholipid bond lengths were constrained using the parallel version of the linear constraint solver algorithm (P-LINCS)[36], while the SETTLE algorithm[37] was used for constraining water molecules.

Initial configurations for the membrane pore stability studies were built starting from pre-equilibrated systems and by removing several molecules to form a large enough cavity for the next steps. In these pore stability studies, we only used compound **1**. The final systems consisted of 234 DMPC, 52 glycoside (GL) in 196 DMPC (~20 %), or 124 GL in 124 DMPC (50 %) solvated with ~12000 water molecules. A three-step simulation procedure was then performed to force water molecules to fill the cavity and subsequently allow glycosides and/or phospholipids to equilibrate and stabilize the pore. First, a 60 ps simulation was done at constant volume, with the solvent temperature increased up to 500.0 K and position restraints on all glycoside atoms with force constants of 1000 kJ $nm^{-2}$ $mol^{-1}$ in the two directions perpendicular do the membrane normal and 10 kJ $nm^{-2}$ $mol^{-1}$ in the other. Phospholipid atoms' positions were restrained using a force constant of 1000 kJ $nm^{-2}$ $mol^{-1}$ in all three directions. The second simulation, performed at constant pressure for ~3 ns, was carried out without position restraints to enable free motion of the lipids, the solvent was cooled down to 308 K and the pressure was kept constant at 1 bar in the bilayer plane direction and at 100 bar in the bilayer normal direction ($\tau_p$ = 0.5 ps), to force the water molecules to stay inside the cavity. The conformations used in the final simulations were selected as the largest pore sizes allowed, before the lipid bilayer sees its periodic image over direction of the membrane normal. This last step, consisted in 30 ns simulations at constant area, 308 K and 1 bar ($\tau_p$ = 2.0 ps, in the direction of the membrane normal), to allow for the lipid positions and pore size equilibrations. The final conformations exhibited pore sizes with ~1800 water molecules. A water molecule is considered inside the pore when the *zz* coordinate of its oxygen atom is between the average *zz* positions, per monolayer, of the 4th methylene of the DMPC tails. Five NPT production replicates were performed (308 K and 1 bar with $\tau_p$ = 2.0 ps), starting from different conformations extracted from the last equilibrated nanoseconds of the constant area simulation. The surface tension simulations were performed by applying a negative lateral pressure in the *xy* plane of the simulation box. The lateral pressure values used were 1, -10, -15, -20, and -30 bar, corresponding to different surface tension values, calculated using Equation 1:

$$ST = L_z (P_z - \langle P_{lat} \rangle) \qquad [1]$$

where $L_z$ is the length of the simulation box in the *zz* dimension, $P_z$ is the pressure along the *zz* dimension, and $P_{lat}$ is the average lateral pressure in the *xy* plane. Since the $L_z$ value is varying during the pore-closing process, the ST values are also fluctuating over time. Therefore, the reported ST values were estimated using $L_z$ from the final segment of each simulation (corresponding to almost closed pores).

For the simulations of glycoside/phospholipid bilayer, systems consisting of GL/DMPC binary mixtures were constructed starting from a pre-equilibrated lipid bilayer containing 256 DMPC molecules and 8821 water molecules, from which a variable number of phospholipids (*n* = 2, 10, 30, 60 or 128) was randomly removed and replaced by equal number of glycoside molecules (GL = compounds **1**, **6**, **7**, DG or OG). Equilibrium configurations of each two-component bilayer system at different molar fractions (GL/DMPC = 0.8%, 3.9%, 11.7%, 23.4% or 50.0%; 75.0% was also tested for OG simulations) were subsequently simulated in triplicate, 100 ns long each. A 100% DMPC bilayer was also simulated, with a

production time of 300 ns (single replicate), as a control for analysis purposes. In all simulations, the last 80 ns of production were used for analysis.

All systems were energy minimized prior to the MM/MD production simulations according to the following three-step procedure: (i) ~ $10^4$ steps with the steepest descent algorithm (without constraints) followed by (ii) ~ $10^4$ steps using the limited-memory Broyden–Fletcher–Goldfarb–Shanno (*l*-BFGS) algorithm (also unconstrained) and (iii) a final minimization using the steepest descent algorithm again, but with all bond lengths constrained. MM/MD simulations were subsequently initialized in three-steps. First, a 50 ps simulation, which was carried out with the positions of all atoms restrained using a force constant of $10^3$ kJ nm$^{-2}$ mol$^{-1}$. In the second step, a 300 ps simulation was performed with all atoms again position-restrained but using a force constant of $10^2$ kJ nm$^{-2}$ mol$^{-1}$ instead. Finally, only the sugar ring and phosphorus atoms were restrained with force constants of $10^2$ kJ nm$^{-2}$ mol$^{-1}$ and $10^3$ kJ nm$^{-2}$ mol$^{-1}$, respectively, for further 100 ps. The equilibrated conformations obtained as described above were used as starting systems for the several production simulations performed.

Data analyses were performed using tools available from the GROMACS software package[26,27] or others developed in-house. Plotted data was averaged over at least three replicates, depending on the system. Ensemble averages were calculated considering only the contribution of equilibrated segments of the trajectories. The total bilayer area was determined from the dimensions of the simulation box along the bilayer plane, averaging over time from all simulations. The degree of phospholipid acyl chain ordering was assessed by calculating the deuterium order parameters, $|S_{\mathrm{CD}}|$, defined as in Equation 2:

$$|S_{\mathrm{CD}}| = \frac{1}{2}\langle 3\cos^2\theta - 1\rangle \qquad [2]$$

where $\theta$ is the angle between the vector along the C-D bond and the bilayer normal, and the angular brackets denote an average over time for the entire ensemble. This property can be determined experimentally by $^2$H NMR spectroscopy and provides a measure of the orientational anisotropy of each C-D bond with respect to a reference axis[38]. The bilayer thickness, often expressed as the headgroup spacing (i.e. the average distance between phospholipid headgroup phosphates), $D_{HH}$, was computed as twice the average distance between the positions (component parallel to the membrane normal) of all phosphorus atoms and the center of mass of the bilayer, averaging over the entire trajectory. Errors were computed as the correlation-corrected standard error in the mean using standard methods based on the time-dependent autocorrelation function of the given property, which was used to estimate the number of independent blocks in the simulations[39]. The correlation time was taken as the value at which the autocorrelation function becomes lower than 0.1.

# 4 | Supplementary Discussion

## 4.1. Conformational analysis of *threo*-pentopyranosides

NMR structure elucidation of the β-L-anomer **8** suggested the adoption of an unusual conformation when the sample was solved in chloroform-*d*. Its anomeric proton signal appeared as a broad triplet with coupling constants $^3J_{1,2eq} \approx {}^3J_{1,2ax}$ = 3.5 Hz, with an order of magnitude common to that of α-anomers. Both stereoisomers **7** and **8** presented similar H-1 and C-1 resonances, with H-1 chemical shifts of δ=4.84 ppm for **7** and δ=4.80 ppm for **8** and those of C-1 appearing at δ=97.6 ppm and δ=98.1 ppm, respectively. In addition, the anomeric proton of the α-glycoside was observed as a broad double doublet with coupling constants $^3J_{1,2ax}$ = 3.3 Hz, $^3J_{1,2eq}$ = 1.7 Hz. Altogether, these results are not in agreement with the expected equatorial orientation of the anomeric substituent for the β-L-glycoside, that usually adopts a $^1C_4$ conformation. Instead, they suggest an unusual $^4C_1$ conformation, featuring all substituents of the pentopyranosyl ring in axial orientation, in chloroform-*d*. As anticipated, NMR spectra of the two stereoisomers acquired in methanol-$d_4$ followed the expected pattern and the typical $^1C_4$ conformation could be unambiguously assigned. While $^1$H NMR data obtained for the α-anomer **7** in chloroform-*d* and methanol-$d_4$ were very similar, the H-1 signal of the β-anomer **8** was now visible at $\delta$ = 4.53 ppm as a double doublet, with $^3J_{1,2eq}$ = 1.7 Hz and the expected *trans*-diaxial coupling constant of H-1 with H-2a ($^3J_{1,2ax}$ = 8.4 Hz). Moreover, the chemical shift of C-1 in methanol-$d_4$ was now found at 101.5 ppm, also supporting the β-configuration in a $^1C_4$ conformation (Supplementary Fig. 2). In the case of enantiomers **17** and **18**, opposing conformational preferences were observed under similar conditions, accordingly.

These results provide evidence that the solvent environment modulates conformer populations of β-L-glycoside **8** in solution. The proposed conformational preferences were further analyzed by DFT calculations (PBE0/6-311G**)[22,23]. The geometries of the two chair conformers ($^4C_1$ and $^1C_4$) of glycosides **7** and **8** were optimized in both chloroform and methanol (IEFPCM/SMD)[24,25], and the relative Gibbs free energies obtained are provided in Supplementary Table 1.

In agreement with experimental data, the considerable energy differences obtained for the α-glycoside **7** support a clear preference for the $^1C_4$ conformer in solution, regardless of the solvent. In contrast, in the case of β-glycoside **8**, the energy of the $^1C_4$ conformer is higher by 1.0 kcal mol$^{-1}$ in chloroform, pinpointing to a greater stability for the $^4C_1$ conformation in this case. This thermodynamic preference may be rationalized by stereoelectronic influences, namely the contribution of the anomeric effect given the low dielectric constant of the media. Furthermore, considering the structural flexibility of **8**, a plausible driving force towards the lower energy calculated for the $^4C_1$ conformer is intramolecular hydrogen bonding involving the 3-hydroxy group and the exocyclic oxygen, as shown in Supplementary Fig. 3. This weak non-covalent interaction (O···O distance of 2.8 Å and O–H···O angle of 143º) may be exacerbated in chloroform which features poor hydrogen bonding capability. In turn, a very negligible energy difference was obtained for the two conformations of **8** in methanol, although experimental data provides unambiguous evidence that only the $^1C_4$ conformer is populated. Accounting for the great ability of the

protic solvent to establish competitive intermolecular hydrogen bonds and further decrease the anomeric effect, we conclude that this result arises from an overestimation of the intramolecular hydrogen bond in methanol, resulting from a limited description of solvation effects by the implicit model used in the calculations.

Our results expose pentopyranoside anomeric chemistry as particularly challenging regarding endo and exo-anomeric effects, resulting in unpredictable inversion of conformation and reaction outcomes when compared to the hexopyranoside analogs, in line with findings reported on other systems, where conformation inversion of xylopyranosides and sulfur exo-anomeric effect played an important role in reaction outcome[40].

## 5 | Supplementary References


1. Martins, A. et al. Tuning the bioactivity of tensioactive deoxy glycosides to structure: antibacterial activity versus selective cholinesterase inhibition rationalized by molecular docking. *Eur. J. Org. Chem.* **8**, 1448–1459 (2013).

2. Petrache, H. I., Dodd, S. W., & Brown, M. F. Area per lipid and acyl length distributions in fluid phosphatidylcholines determined by $^{2}$H NMR spectroscopy. *Biophys. J.* **79**, 3172 (2000).

3. Kučerka, N., Nieh, M.-P. & Katsaras, J. Fluid phase lipid areas and bilayer thicknesses of commonly used phosphatidylcholines as a function of temperature. *BBA-Biomembranes* **1808**, 2761–2771 (2011).

4. Gualerzi, C. O., Brandi, L., Fabbretti, A. & Pon, C. L. Antibiotics: Targets, Mechanisms and Resistance, John Wiley & Sons, Weinheim, Germany (2013).

5. Khattar, Z. A. et al. The dlt operon of *Bacillus cereus* is required for resistance to cationic antimicrobial peptides and for virulence in insects. *J. Bacteriol.* **191**, 7063–7073 (2009).

6. Salvetti, S., Faegri, K., Ghelardi, E., Kolsto, A.-B. & Senesi, S. Global gene expression profile for swarming *Bacillus cereus* bacteria. *Appl. Environ. Microbiol.* **77**, 5149–5156 (2011).

7. Ghorab, M. M., Ismail, Z. H., Abdalla, M. & Radwan, A. A. Synthesis, antimicrobial evaluation and molecular modelling of novel sulfonamides carrying a biologically active quinazoline nucleus. *Arch. Pharm. Res.* **36**, 660–670 (2013).

8. Cunha, W. et al. Evaluation of the antibacterial activity of the methylene chloride extract of *Miconia ligustroides*, isolated triterpene acids, and ursolic acid derivatives. *Pharm. Biol.* **48**, 166–169 (2010).

9. Simm, R. et al. BC4707 is a major facilitator superfamily multidrug resistance transport protein from *Bacillus cereus* implicated in fluoroquinolone tolerance. *PLoS One* **7**, 1–13 (2012).

10. Chaieb, K., Kouidhi, B., Jrah, H., Mahdouani, K. & Bakhrouf, A. Antibacterial activity of thymoquinone, an active principle of *Nigella sativa* and its potency to prevent bacterial biofilm formation. *BMC Complem. Altern. M.* https://doi.org/10.1063/1.3359469 (2011).

11. Vogler, A. J. et al. Molecular analysis of rifampin resistance in *Bacillus anthracis* and *Bacillus cereus*. *Antimicrob. Agents Chemother.* **46**, 511–513 (2002).

12. World Health Organisation: Guidelines for ATC classification and DDD assignment. Methodology Norwegian Institute of Public Health. https://www.whocc.no/atc_ddd_index/ (2018).

13. Chen, H. et al. An efficient method for the synthesis of pyranoid glycals. *Carbohydr. Res.* **431**, 42–46 (2016).

14. Xu, Y. et al. A convenient and efficient synthesis of glycals by zinc nanoparticles. *RSC Adv.* **4**, 46662–46665 (2014).

15. Zhao, J., Wei, S., Ma, X. & Shao, H. A simple and convenient method for the synthesis of pyranoid glycals. *Carbohydr. Res*. **345**, 168–171 (2014).

16. van Steijn, A. M. P., Kamerling, J. P. & Vliegenthart, J. F. G. Synthesis of a spacer-containing repeating unit of the capsular polysaccharide of *Streptococcus pneumoniae* type 23F. *Carbohydr. Res.* **211**, 261–277 (1991).

17. Cachatra, V. et al. Wittig reaction: domino olefination and stereoselectivity DFT study. Synthesis of the Miharamycins' bicyclic sugar moiety. *Org. Lett.* **17**(*22*), 5622–5625 (2015).

18. Clinical and Laboratory Standards Institute. Methods for Dilution Antimicrobial Susceptibility Tests for Bacteria that Grow Aerobically. Approved Standard, 10th Ed., NCCLS document M7–A4 (2015).

19. KEGG (Kyoto Encyclopedia of Genes and Genomes) database, http://www.genome.jp/kegg/ (2018).

20. Mäntynen, V. & Lindström, K. A rapid PCR-based DNA test for enterotoxic *Bacillus cereus. Appl. Environ. Microbiol.* **64**, 1634–1639 (1998).

21. Frisch, M. J. et al. Gaussian 09, Revision A.01. Gaussian, Inc., Wallingford CT (2009).

22. Adamo, C. & Barone, V. Toward reliable density functional methods without adjustable parameters: the PBE0 model. *J. Chem. Phys.* **110**, 6158–6170 (1999).

23. Krishnan, R., Binkley, J. S., Seeger, R. & Pople, J. A. Self-consistent molecular orbital methods. XX. A basis set for correlated wave functions. *J. Chem. Phys.* **72**, 650–654 (1980).

24. Scalmani, G. & Frisch, M. J. Continuous surface charge polarizable continuum models of solvation. I. General formalism, *J. Chem. Phys.* **132**, 114110 (2010).

25. Marenich, A. V., Cramer, C. J. & Truhlar, D. G. Universal solvation model based on solute electron density and on a continuum model of the solvent defined by the bulk dielectric constant and atomic surface tensions. *J. Phys. Chem. B.* **113**, 6378–6396 (2009).

26. van der Spoel, D. et al. GROMACS: fast, flexible, and free. *J. Comput. Chem.* **26**, 1701–1718 (2005).

27. Hess, B., Kutzner, C., van der Spoel, D. & Lindahl, E. GROMACS 4: algorithms for highly efficient, load-balanced, and scalable molecular simulation. *J. Chem. Theory Comput.* **4**, 435–447 (2008).

28. Schmid, N. et al. Definition and testing of the GROMOS force-field versions 54A7 and 54B7. *Eur. Biophys. J.* **40**, 843–856 (2011).

29. Poger, D., van Gunsteren, W. F. & Mark, A. E. A new force field for simulating phosphatidylcholine bilayers. *J. Comput. Chem.* **31**, 1117–1125 (2010).

30. Hansen, H. S. & Hünenberger, P. H. A reoptimized GROMOS force field for hexopyranose-based carbohydrates accounting for the relative free energies of ring conformers, anomers, epimers, hydroxymethyl rotamers, and glycosidic linkage conformers. *J. Comput. Chem.* **32**, 998–1032 (2011).

31. DeLano, W. L. The PyMOL molecular graphics system. DeLano Scientific, Palo Alto, CA, USA. http://www.pymol.org (2002).

32. Hermans, J., Berendsen, H. J. C., van Gunsteren, W. F. & Postma, J. P. M. A consistent empirical potential for water-protein interactions. *Biopolymers* **23**, 1513–1518 (1984).

33. Smith, P. E. & van Gunsteren, W. F. Consistent dielectric properties of the simple point charge and extended simple point charge water models at 277 and 300 K. *J. Chem. Phys.* **100**, 3169–3174 (1994).

34. Bussi, G., Donadio, D. & Parrinello, M. Canonical sampling through velocity rescaling. *J. Chem. Phys.* **126**, 014101–014107 (2007).

35. Berendsen, H. J. C., Postma, J. P. M., van Gunsteren, W. F., DiNola, A. & Haak, J. R. Molecular dynamics with coupling to an external bath. *J. Chem. Phys.* **81**, 3684–3690 (1984).

36. Hess, B. P-LINCS: A parallel linear constraint solver for molecular simulation. *J. Chem. Theory Comput.* **4**, 116–122 (2008).

37. Miyamoto, S. & Kollman, P. A. SETTLE: An analytical version of the SHAKE and RATTLE algorithm for rigid water models. *J. Comput. Chem.* **13**, 952–962 (1992).

38. Vermeer, L. S., de Groot, B. L., Réat, V., Milon, A. & Czaplicki, J. Acyl chain order parameter profiles in phospholipid bilayers: Computation from molecular dynamics simulations and comparison with $^{2}H$ NMR experiments. *Eur. Biophys. J.* **36**, 919–931 (2007).

39. Allen, M. P. & Tildesley, D. J. Computer Simulation of Liquids, Oxford Univ. Press, New York, USA (1987).

40. Crich, D., Mataka, J., Zakharov, L. N., Rheingold, A. L. & Wink, D. J. Stereoselective formation of glycosyl sulfoxides and their subsequent equilibration: ring inversion of an α-xylopyranosyl sulfoxide dependent on the configuration at sulfur. *J. Am. Chem. Soc.* **124**, 6028–6036 (2002).
